# Spatiotemporal co-regulation of IL2 and TCR receptors reveals early antigen-driven synaptic synergy in human CD8^+^ T cells

**DOI:** 10.1038/s41467-026-77110-1

**Published:** 2026-10-07

**Authors:** Jesusa Capera, María Navarro-Pérez, Ashwin K. Jainarayanan, Salvatore Valvo, Lina Chen, Panyu Fei, Sarah Flannery, Roman Fischer, Saso Cemerski, Simon Low, Steven N. Quayle, Raymond J. Moniz, Anish Suri, Michael L. Dustin

**Affiliations:** 1 The Kennedy Institute of Rheumatology, Nuffield Department of Orthopaedics, Rheumatology and Musculoskeletal Sciences, University of Oxford, Oxford, UK; 2 Target Discovery Institute, University of Oxford, Oxford, UK; 3 Cue Biopharma, Boston, MA USA; 4 CAMS Oxford Institute, Chinese Academy of Medical Sciences & Peking Union Medical College, University of Oxford, Oxford, UK

**Keywords:** Interleukins, Immunotherapy, CD8-positive T cells

## Abstract

Interleukin 2 (IL2) promotes T cell proliferation and differentiation, making it a central target in immunotherapies. Following antigen recognition, IL2 receptor (IL2R) signaling polarizes towards the immunological synapse (IS). However, how IL2 function is integrated during earlier antigen-dependent T cell synapses remains unknown. Here, we employ a supported-lipid bilayer system and TIRF microscopy to monitor the IL2R subunits and their spatiotemporal organization relative to T cell receptor (TCR) at the IS of CD8^+^ T cells. We show that TCR and IL2 signaling overlap in space and time and synergize when simultaneously triggered. Using Immuno-STATs, a class of immunotherapeutics in clinical testing that co-deliver IL2 and peptide major histocompatibility complex (pMHC), we demonstrate synergistic TCR and IL2R signaling that drives efficient expansion of antigen-specific CD8^+^ T cells. Together, these findings establish the IS as a platform for coordinated cytokine and antigen receptor signaling, providing a mechanistic framework for more selective antigen-specific immunotherapies.

## Abstract (web summary)

T cells modulate their sensitivity to regulatory molecules, such as IL-2, by dynamically integrating intrinsic receptor signals to ensure efficient immune responses. Here, the authors investigate the spatiotemporal regulation of IL-2 receptor subunit localization at the immunological synapse in relation to the T cell receptor and antigen-driven early signaling pathway initiation, and reveal spatiotemporal pairing and enhanced positive crosstalk, providing a mechanistic explanation for the superior outcome of ImmunoSTAT therapeutics in inducing T cell responses.

## Introduction

Interleukin 2 (IL2) is a 15 kDa cytokine primarily produced by activated CD4^+^ and, to a lesser extent, by activated CD8^+^ T cells. It acts as a potent autocrine/paracrine mitogenic signal and regulates the expansion and differentiation of the activated T cell subsets, crucial for immunity against cancers and infectious diseases^1^. IL2 also plays a critical role in the development and maintenance of regulatory T cells (Treg)^2^. High-dose IL2 is an approved therapy for metastatic melanoma and renal cell carcinoma, with clinical benefit to a fraction of patients^1^. However, despite its efficacy, the broad utility of IL2 for immunotherapy of cancers has been impeded due to toxicities related to indiscriminate activation of immune cells^3^. To overcome these challenges, strategies aim to bias IL2 activity to tumor-specific immune cells, minimizing systemic effects and creating a “therapeutic index” for IL2—a clear separation between the toxic and effective doses. Such approaches include generating IL2 variants with different receptor affinities^4^, protease-sensitive masked IL2 molecules for local tumor-specific effects^5^, and, more recently, directing IL2 to T cells^6,7^. However, none of these approaches selectively target IL2 to tumor-specific T cells, which are the desirable cell population for therapeutic benefit and present in extremely low precursor frequencies within the polyclonal T cell repertoire^8^. To this end, a class of molecules termed Immuno-STATs (IST) was engineered to selectively deliver IL2 to antigen-specific CD8^+^ T cells by combining affinity-reduced IL2 with peptide major histocompatibility complex (pMHC) molecules^9,10^. Preclinical studies have demonstrated that ISTs selectively bind, activate and expand naturally occurring antigen-specific CD8^+^ T cells from peripheral blood mononuclear cells (PBMC) of healthy and cancer patients. These studies also revealed significant anti-tumor activity in vivo along with induction of immunologic memory and a favorable safety profile, with no significant expansion of Tregs observed either in vitro or in vivo^9^. ISTs are currently being tested in phase I clinical trials for human papilloma virus (HPV)-driven recurrent/metastatic head and neck cancer, as well as Wilms’ Tumor 1-positive recurrent/metastatic solid tumors with favorable tolerability (i.e., no major IL2-related toxicities) and encouraging evidence of clinical benefit in late-stage metastatic cancer patients and increased response rates in combination with anti-PD-1 therapy in frontline metastatic patients^11,12^^,^.

ISTs offer a targeted approach to co-engage the IL2 receptor (IL2R) and the T cell receptor (TCR) on tumor-specific T cells, yet how these two signaling pathways integrate at the immunological synapse (IS) to initiate T cell activation remains unexplored, leaving open questions about their combined role in driving effective immune responses. The IL2 receptor consists of three transmembrane subunits, which bind to IL2 with varying affinities. The IL2RA (CD25) forms the low-affinity receptor. The IL2RB (CD122) and IL2RG (CD132) form the intermediate-affinity receptor, and all three form the high-affinity receptor. Only the IL2RB and IL2RG contain signaling cytoplasmic tails, primarily signaling via the Janus Kinase/Signal transducer and activator of transcription (JAK/STAT) pathway^1,13–15^. IL2 binding to IL2RB/IL2RG recruits JAK1 and JAK3, leading to STAT activation and nuclear translocation to regulate gene expression^16,17^. The STAT family is composed of seven members and STAT5A and B are the more potently activated by IL2 receptors^18^. IL2RG, also known as the common cytokine receptor γ chain (γc), is shared by IL4, IL7, IL9, IL15, and IL21 receptors, while IL2RB is shared by IL2 and IL15 receptors^19^. Both CD4^+^ and CD8^+^ T cells transiently express high-affinity IL2 receptors following TCR engagement, peaking within hours post-antigen challenge and declining after 24 h^14^. The strength of IL2 receptor signaling influences CD8^+^ T cell differentiation, with high IL2RA expression driving effector cell proliferation, while lower IL2RA expression promotes memory cell differentiation^1,20,21^. Additionally, IL2 shapes the effector CD8^+^ T cell repertoire, promoting stemness-like features through TCF-1 expression and preventing terminal exhaustion^22,23^.

IL2 functions primarily through paracrine or autocrine signaling following T cell–dendritic cell (DC) interaction, with activated T cells forming large, dynamic clusters sustained by LFA-1 interactions^24,25^. These clusters exhibit directional IL2 secretion and polarization of the IL2 receptor complex towards T-T synapses, enhancing pSTAT5 signaling and IL2 action^26^. In addition, IL2 directly affects CD8^+^ T cell sensitivity to antigenic peptides, with CD4^+^ effector T cells potentially releasing IL2 to CD8^+^ T cells as they synapse with DCs^27^. The T-DC IS can be modeled using supported lipid bilayers (SLBs), which simplifies the IS into a 2D model with concentric compartments for effector function (central), adhesion and transport (peripheral) and signal initiation/costimulation (distal ring/corolla)^28^. F-actin dependent TCR microclusters are sites of TCR signaling^29,30^ and are analogous to microvilli and podosomes in 3D models^31–33^. IL2 roles in the IS between CD4^+^ or CD8^+^ T cells and DCs also involve Tregs, which intercept IL2 to enhance their survival while starving autoreactive T cells, impacting the fitness of these cells^34^. This dual function of IL2 is critical for immunotherapy, where targeted delivery of IL2 must secure a balance between its immunosuppressive and pro-inflammatory effects to maximize therapeutic benefits in specific disease contexts.

In this study, we investigate how IL2 receptor signaling is spatially and functionally integrated with TCR signaling during antigen-specific IS formation. We localize IL2 action within the IS and identify a synergistic relationship between the IL2 and TCR signaling pathways based on their spatiotemporal proximity at the IS. Using Immuno-STATs, we further show that simultaneous delivery of peptide–MHC and IL2 promotes IL2R assembly, enhances its proximity to the TCR, and amplifies both signaling pathways, demonstrating that the IST strategy goes beyond simply *cis*-targeting the IL2R and the TCR by exploiting their natural synergy to promote efficient activation and expansion of antigen-specific CD8⁺ T cells. This selective activation creates a therapeutic index for IL2 by favoring tumor-specific T cell responses while avoiding systemic activation of other immune cells. Together, these findings establish the IS as a platform for coordinated integration of cytokine and antigen receptor signaling and provide a mechanistic framework for the rational design of cytokine-based immunotherapies that exploit spatially constrained signaling to achieve selective and effective activation of disease-specific T cells.

## Results

### Organization of the IL2 receptor subunits at the IS

**Fig. 1:**
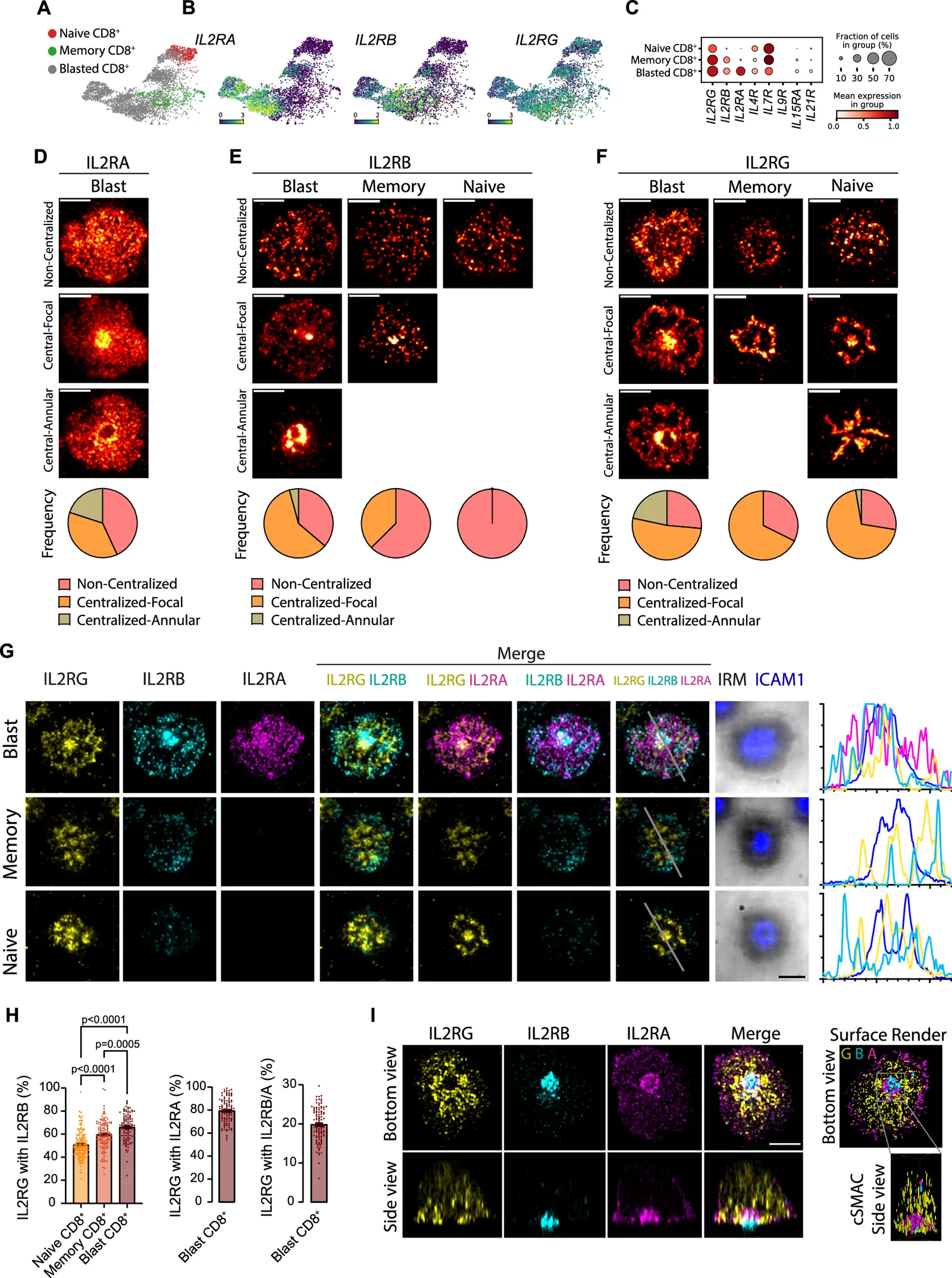
Distribution of the IL2 receptor complex at the IS. **A** UMAP dimensionality reduction embedding of blasted, memory and naive CD8^+^ T cells colored by orthogonally generated clusters labeled by manual cell type annotation. **B** UMAP embedding of scRNAseq dataset of CD8^+^ T cell populations colored by expression profiles of IL2RA, IL2RB and IL2RG. **C** Dot plots depicting mean expression and fraction of cells expressing common-gamma chain interleukin receptor genes. **D**–**F** RA14 CD8^+^ T cells were exposed to Ag-specific SLBs in the presence of 50 U/ml of IL2 to form ISs and fixed after 10 min. Panels show TIRF images of the different distribution phenotypes (Non-centralized, Centralized-Focal and Centralized-Annular) observed for the IL2RA (**D**), IL2RB (**E**) and IL2RG (**F**). Brightness and contrast were adjusted independently for each image to facilitate visualization of signal distribution. IL2RA imaging is omitted for Naive and Memory subsets due to lack of expression. Frequency for each phenotype was determined by visual inspection and is represented as parts of a whole for each T cell state. Data are the mean of three independent donors with >50 cells/donor. **G** TIRF images of blast, memory and naive RA14 CD8^+^ T cells forming ISs with Ag-specific SLBs in the presence of 50 U/ml of IL2 and fixed after 10 min. Histograms (right) show the intensity line profile for the different proteins. Representative images from three independent experiments with similar results are shown. **H** Percentage of the total IL2RG intensity that is within the area covered by IL2RA, IL2RB or within the intersection between the IL2RB and IL2RA. See Supplementary Fig. 2D for a more details. Dots shows the mean ± SE, *n* = 152 (naive), 126 (memory) and 107 (blast) cells, from three independent donors. Kruskal–Wallis test. **I** Representative z-stack Airyscan reconstruction of a RA14 CD8^+^ blast forming a IS on an Ag-specific SLB in the presence of 50 U/ml of IL2. Sample was fixed after 10 min of SLB exposure. The bottom view shows the lowest XY plane at the cell–SLB interface; the side view shows an XZ reconstruction with the SLB at the bottom. Scale bar is 5 μm.

T cell responsiveness to IL2 relies on the expression of IL2 receptor subunits and other interleukin receptors competing for the common-gamma chain^1,35^. Therefore, the expression and distribution of the IL2 receptor subunits was analyzed at the IS of three different states of CD8^+^ T cell differentiation: naive, memory and activated lymphoblast (blast). First, differential expression of the IL2 receptor subunits was confirmed using a publicly available sc-RNAseq dataset. UMAP analysis established a distinct cluster of CD8^+^ T cells with greater heterogeneity in memory cells and blasts (Fig. 1A). *IL2RA* was primarily detected in blasts (Fig. 1B, C). *IL2RB* was well expressed in memory and blast CD8^+^ T cells and was nearly absent in the naive state, while *IL2RG* was well expressed across all states (Fig. 1B, C). To provide further context to understand the differential responses to IL2 across the different T cell states, expression of other interleukin receptor subunits potentially competing for the γ_c_ (IL2RG) was also analyzed. The *IL7R* was mainly expressed in the naive and memory states, with markedly decreased expression in blasts (Fig. 1C).

Next, the distribution of the IL2 receptor subunits was visualized at the IS of antigen (Ag)-specific CD8^+^ T cells. Naive, memory and blast CD8^+^ T cells were electroporated with mRNA encoding the CMV pp65 RA14 TCR and exposed to supported lipid bilayers (SLB) presenting HLA-A201 complexes with the CMV pp65_495-502_ peptide, in addition to ICAM-1, CD80 and CD58, before imaging with total internal reflection fluorescence microscopy (TIRFM), which isolates fluorescence signals within 200 nm of the SLB. The experimental workflow is outlined in Supplementary Fig. 1A. Briefly, Naive and Memory CD8⁺ T cells were cultured without IL2 supplementation until the day of the experiment, whereas blast CD8⁺ T cells were maintained in the presence of IL2. As expected, IL2RA expression was minimal for Naive and Memory and high for blast CD8^+^ T cells (Supplementary Fig. 1B–E). Prior to imaging, cells were washed and resuspended in fresh medium with or without IL2, as indicated for each experimental condition and time point. Electroporation efficiency was 84% ± 1.21 (SEM), as determined by cognate tetramer staining (Supplementary Fig. 1F). IL2 receptor subunits displayed a punctate pattern that could be classified into non-centralized (puncta distributed throughout the synaptic contact without accumulation at the center), centralized-focal (dense clustering of puncta at center) and centralized-annular (a central ring of puncta) (Fig. 1D–F and Supplementary Fig. 2A). IL2RA was centralized in most of the blast ISs (Fig. 1D). IL2RB was mostly centralized in blasts, but non-centralized patterns were more prominent in memory cells and dominant in naive cells (Fig. 1E). IL2RG was mainly centralized in all T cell states and formed a peripheral corolla, which was observed as a secondary feature in all types of CD8^+^ T cell states (Fig. 1F). The centralized (focal and annular) phenotypes of IL2 receptor subunits were more frequent when the IS was established in the presence of IL2 (Supplementary Fig. 2A). Supplementary Fig. 2B provides a quantitative comparison of synaptic intensity for the three IL2 receptor subunits across the T cell subsets, consistent with their respective whole-cell expression levels. IL2 treatment also decreased the abundance of the IL2 receptor subunits at the IS of naive and blast CD8^+^ T cells (Supplementary Fig. 2C).

Simultaneous visualization of the three subunits at the IS showed distinct localizations, with some puncta containing two or all three subunits (Fig. 1G, H). The abundance of the subunits within these clusters was dependent on the T cell differentiation state. For example, blast cells showed the highest percentage of IL2RG with IL2RB or IL2RB/A clusters (potential intermediate or high affinity IL2 receptors, respectively), followed by memory cells (Fig. 1H and Supplementary Fig. 2D). This pattern is directly related to the expression levels of IL2RB and IL2RA and inverse to the expression of other common-gamma chain receptors like IL7R (Fig. 1C). Pairwise correlations were highest in blasts and lower for memory and naive CD8^+^ T cells (Supplementary Fig. 2D).

Three-dimensional imaging of cells forming IS with the SLBs indicated that centrally accumulated subunits were partly above the plane of the SLB, suggesting internalization or release through ectocytosis of extracellular vesicles (EV) or secretion in exosomes^36^. Intracellular IL2RA and IL2RB were accumulated at the cytoplasmic side of the cSMAC, while intracellular IL2RG was present in intracellular compartments scattered throughout the cytoplasm, suggesting different internalization pathways between the IL2 receptor subunits (Fig. 1I).

### IL2/IL2 receptor subunits are released through extracellular vesicles

To further investigate whether IL2 receptor subunits were released in EVs, mass spectrometry (MS) data from EVs released by human CD8^+^ T cells at different differentiation states (naive, memory or total/pooled CD8^+^ T cells) were analyzed. EVs were purified before (resting) and after (activated) treatment with anti-CD3/CD28 Dynabeads^37^. In resting CD8^+^ T cells, only IL2RG was present in EVs, along with EV marker CD81, CD8 and CD3E (Supplementary Fig. 3A). In contrast, EVs from activated CD8^+^ T cells contained IL2, IL2RA and IL2RB, in addition to detectable levels of IL2RG, IL7R and IL9R (Supplementary Fig. 3A). Data was validated by Nano-flow cytometry, which showed that resting EVs were 7.5-times more likely to be IL2RG single positive than activated EVs, whereas activated EVs were 10-times more likely to be single positive for IL2RB than resting EVs. Double positive EVs were rare, suggesting that EV formation operates on the individual receptor subunits rather than on intact complexes (Supplementary Fig. 3B, C). In addition, analysis of intracellular IL2 pathway components within the EV cargo revealed the presence of JAKs and STATs, with STAT5 notably enriched in EVs from activated T cells (Supplementary Fig. 3A).

Next, we evaluated if EVs from activated CD8^+^ T cells, which collectively contain IL2 and all the IL2 signaling components, can activate pSTAT5 in naive CD8^+^ T cells, which have low sensitivity to IL2. Note that size exclusion chromatography used to isolate the EV removes free IL2^37^. Treatment with activated EVs induced a robust increase in pSTAT5 levels compared with untreated controls and showed a tendency toward higher activation than that observed with 50 U/ml of soluble IL2. In addition, IL2-neutralizing antibodies completely inhibited the STAT5 activation through soluble IL2 and reduced the EV-dependent activation of STAT5 (Supplementary Fig. 3D).

Together, these findings suggest that EVs released from activated CD8⁺ T cells can deliver IL2 in a form capable of triggering STAT5 signaling in otherwise IL2-unresponsive naive CD8⁺ T cells. While the observed effect is likely driven primarily by IL2, possible contributions from the transfer of EV-associated IL2 receptor subunits and intracellular signaling molecules cannot be excluded. Further investigation will be required to define the specific EV components responsible for the STAT5 activation observed in naive CD8⁺ T cells.

### Distribution of IL2 receptor activity at the IS

**Fig. 2:**
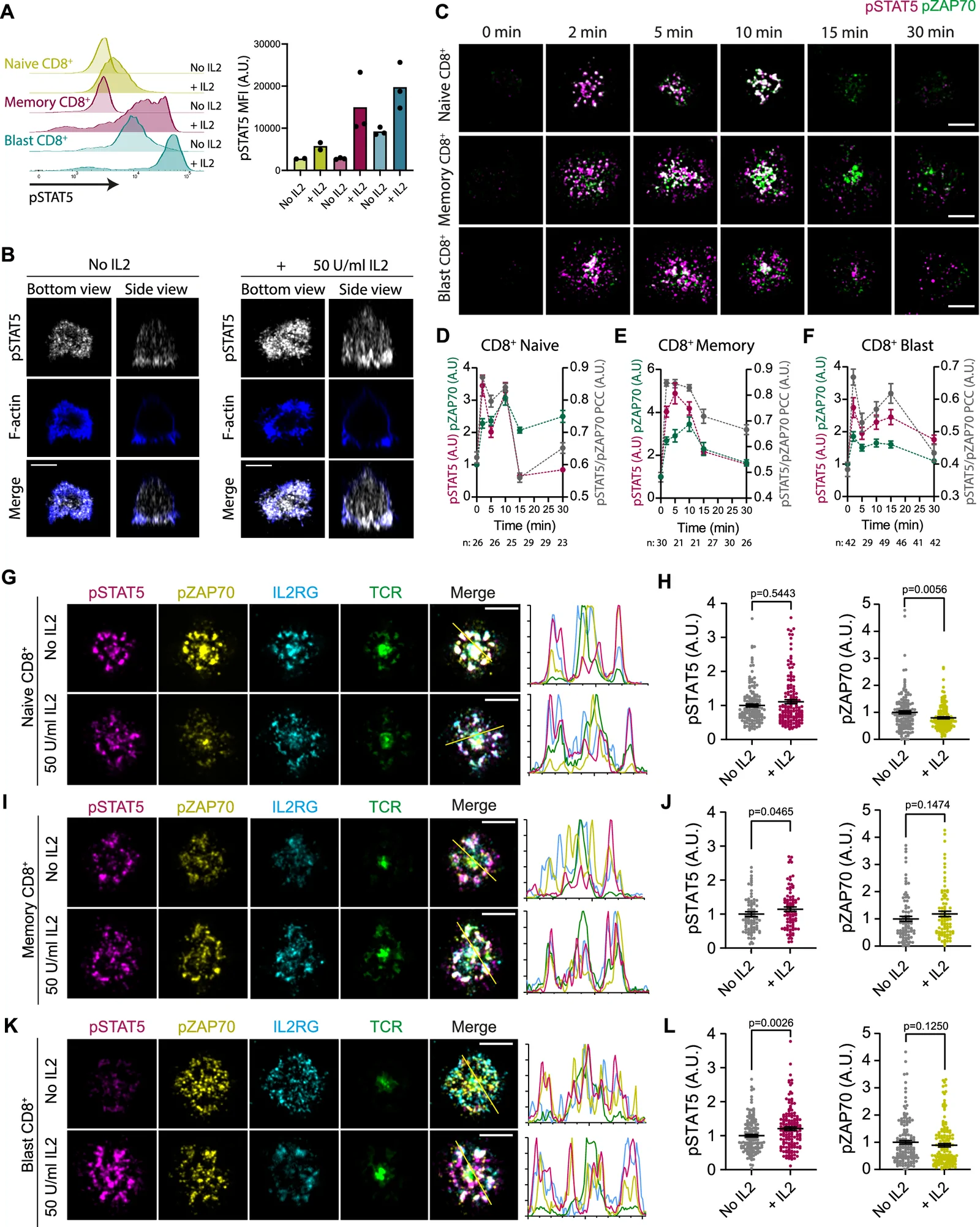
Distribution of IL2 receptor activity at the IS. **A** Histograms of phospho-flow cytometry data showing total levels of pSTAT5 after 10 min incubation in the presence (+IL2) or absence (No IL2) of 50 U/ml of IL2 treatment. Bar plots (right) show the mean ± SE of the pSTAT5 median fluorescence intensity (MFI) from three (blast and memory) or two (naive) independent donors. Gating strategy as in Supplementary Fig. 1B. **B** z-stack Airyscan reconstruction of RA14 CD8^+^ blasts forming a IS on an HLA-A2-p65 peptide bearing SLB in the presence or absence of 50 U/ml of IL2. Sample was fixed after 10 min of SLB exposure. Representative images from two independent experiments with similar results are shown. **C** TIRF images of Naive (top), Memory (middle) and Blast (bottom) CD8^+^ T cells forming an IS with a SLB and fixed after the indicated time-point. 0 min represents cells fixed after 15 min of contact with a SLB without UCHT1 (no IS formation). Representative images from two independent experiments with similar results are shown. **D**–**F** Plots show the mean intensity for pSTAT5 (magenta) and pZAP70 (green) normalized to the 0 min time-point (left *Y* axis) and the Pearson’s Correlation Coefficient (PCC, gray) between pSTAT5 and pZAP70 signals (right *Y* axis) over time for naive (**D**), memory (**E**) and blast (**F**) CD8^+^ T cells. Data are the mean ± SE. **G**–**K** Naive (**G**, **H**), memory (**I**, **J**) or blast (**K**, **L**) RA14 CD8^+^ T cells were exposed to Ag-specific SLBs in the presence or absence of 50 U/ml of IL2 to visualize IS formation. Cells were fixed after 10 min of bilayer exposure and immunostained for pSTAT5 (magenta), pZAP70 (yellow), IL2RG (cyan), TCR (green). On the left, representative TIRF images of ISs. Histograms on the right show the intensity profile across the yellow line shown in the merge images. On the right, dot plots with the mean intensity for pSTAT5 and pZAP70 normalized to the condition without IL2. Data are the mean ± SE, *n* = 150 cells for naive and blast and *n* = 87 cells for memory, from 3 independent donors. Two-tailed Mann–Whitney test. Scale bar is 5 μm.

Responsiveness to IL2 is dependent on the CD8^+^ T cell state of differentiation (Fig. 1)^19^. Upon IL2 treatment, naive CD8^+^ T cells showed limited pSTAT5 compared to the memory or blasted counterparts, the latter showing the highest pSTAT5 levels at the whole cell level (Fig. 2A). In Ag-specific ISs formed by CMV-specific CD8^+^ T cell blasts on SLBs, pSTAT5 signaling was polarized towards the IS, even without soluble IL2 in the media (Fig. 2B). Puncta of pSTAT5 and pZAP70 appeared immediately upon synaptic contact, peaking within 2-15 min and returning near basal levels after 30 min (Fig. 2C–F). In addition, these signaling events initially colocalised at the IS but dissipated at later time points (Fig. 2C–F). Note that in the absence of TCR engagement or exogenous IL2 (0 min timepoint, Fig. 2C), pSTAT5 and pZAP70 signaling is residual at the cell-SLB contact. pSTAT5 puncta, like pZAP70, also overlapped with TCR microclusters (Fig. 2C–F).

Adding IL2 in the media concomitantly to SLB exposure did not affect synaptic pSTAT5 levels in naive CD8^+^ T cells (Fig. 2G, H). However, in both memory (Fig. 2I, J) and blast (Fig. 2K, L) cells, IL2 further boosted the pSTAT5 intensity at the synapse. The pSTAT5 and pZAP70 co-localization in the IS was observed in the IS center and the corolla (Fig. 2G, I, K), both in the presence and in the absence of exogenous IL2. Notably, pSTAT5 accumulated in the active IS and was activated through TCR and co-stimulatory signals, as suggested by the presence of pSTAT5 punta even in the absence of exogenous IL2 or in the naive CD8^+^ T cells, which respond weakly to IL2 (Fig. 2).

To further investigate the effects of IL2 on synapse formation, recruitment of TCR, ICAM-1/LFA1, CD58/CD2 and CD80/CD28 to the synapse was evaluated in naive, memory and blast CD8^+^ T. IL2 did not show major effects on synapse assembly, and the formation of the SMAC compartments was normal. However, in blast CD8^+^ T cells, IL2 enhanced CD2/CD58 interaction in a dose-dependent manner (Supplementary Fig. 4A–F). The synapse contact area was also larger in the presence of IL2 for naive and blast CD8^+^ T cells (Supplementary Fig. 4G).

### Immuno-STATs induce the formation of immunological synapses

**Fig. 3:**
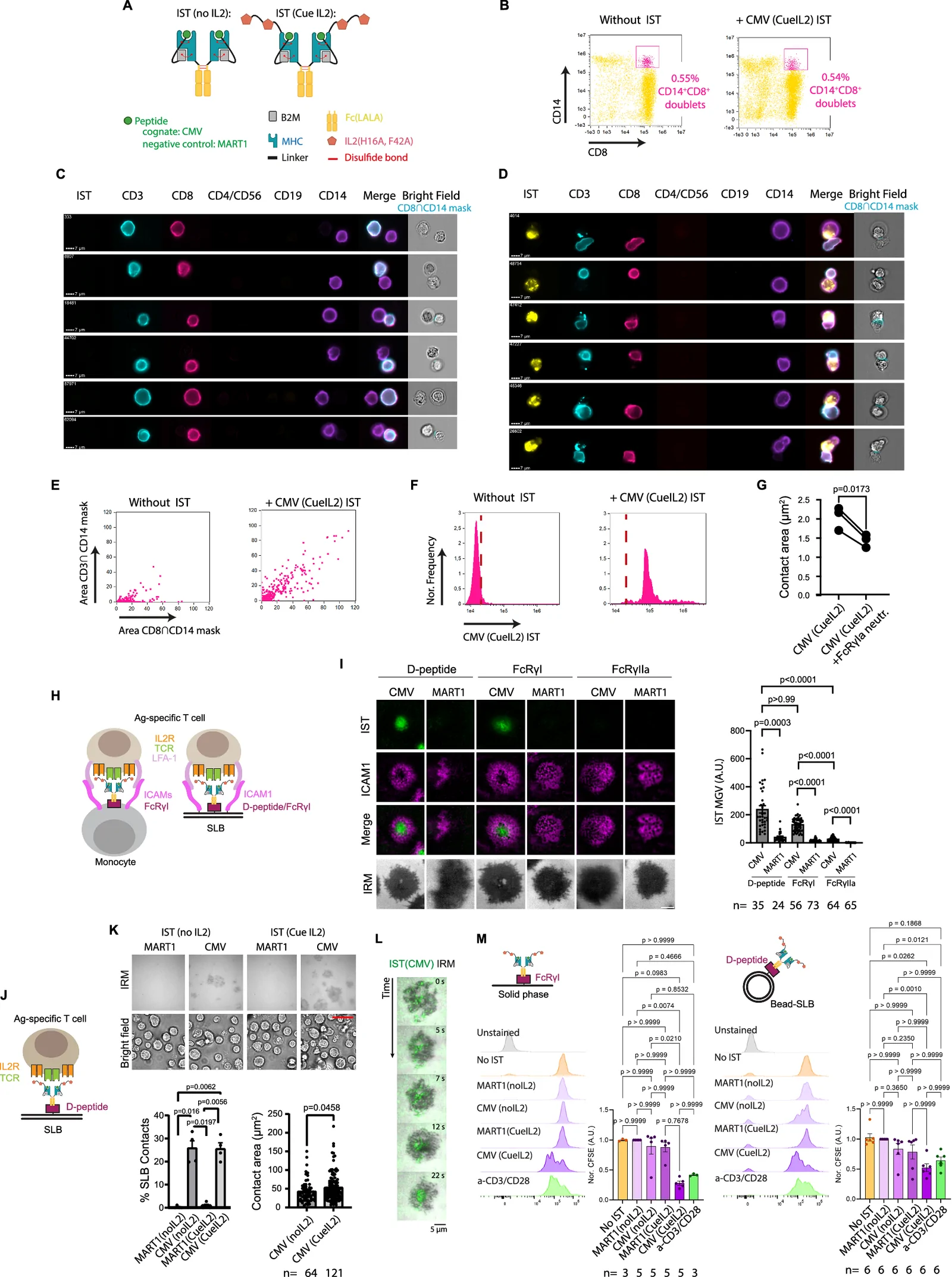
IST induce immunological synapse formation and antigen-dependent proliferation. **A** Cartoon of the IST variants: Two HLA-A*02 complexes with the cognate Ag (CMV pp65) or a non-specific control Ag (MART-1) are connected, or not, to four units of engineered IL2 (Cue IL2). Created in BioRender. Low, S. (2026) https://BioRender.com/1ws9q06). **B** Imaging flow cytometry. Gating strategy for monocyte–T cell doublets from total focused doublets. Percentages are from total focused events. Gating strategy, Supplementary Fig. 5B. CD8^+^ T cell–CD14^+^ monocytes doublets in the absence (**C**) or presence (**D**) of IST treatment. Note the absent or residual contact area in (**C**). Representative images from three independent donors with similar results. Scale bar is 7 μm. **E** Quantification of CD8/CD3 ∩ CD14 mask area defined as the overlap between the two marker pairs. **F** CMV(IL2) IST intensity in CD14^+^CD8^+^ doublet population. **G** Quantification of the CD3 ∩ CD14 mask area in the presence or absence of a FcγRI neutralizing antibody. *n* = 3 independent donors. Two-tailed paired *t*-test (*t* = 7.5, df = 2). **H** Schematic of IST presentation to T cells by monocytes via FcγRI or by SLBs via His-tagged D-peptide or FcγRI and ICAM-1. **I** RA14 CD8^+^ blast T cells on SLBs containing ICAM-1 and the ISTs linked to the SLB through D-peptide, FcRγI or FcRγIIa. Right plot: IST mean gray values. Data are the mean ± SE. Kruskal–Wallis test. Scale bar is 5 μm. **J** Schematic of IST presentation to T cells via His-tagged D-peptide, without additional adhesion molecules. **K** RA14 CD8^+^ blasts were exposed to SLBs containing ISTs variants at SLB-saturating conditions (~2000/µm^2^) alone, linked via D-peptide. Imaging after 15 min. Left plot: Percentage of cells forming contacts (IRM) from all observed (bright field). Data are the mean ± SE of 4 independent donors. One-way matched ANOVA test (*F* = 51.56, DF = 15). Right plot: quantification of the SLB-T cell contact area (IRM). Data are the mean ± SE, Mann–Whitney test. Scale bar is 20 μm. **L** Time-lapse of a RA14 CD8^+^ blast T cell forming contact with a SLB containing only CMV(CueIL2) IST-488. **M** CFSE-based proliferation assay of RA14 CD8^+^ T cells exposed to ISTs. Data are the mean ± SE of independent donors (Kruskal–Wallis test). Gating strategy, Supplementary Fig. 5F.

To further understand the fundamental relationship between IL2 receptor and TCR in the immunological synapse and its therapeutic potential, we characterized IS formation by ISTs. ISTs consist of a human IgG1 Fc domain, with mutations attenuating Fc receptor binding, fused to two copies of an HLA-A2 complex with a covalently tethered peptide. Two copies of human IL2 variant molecules (CueIL2) are then covalently linked in proximity to each HLA-A2 complex. The four CueIL2 domains in the IST have reduced affinity for IL2RA and IL2RB subunits via introduction of H16A and F42A mutations. These IL2 attenuating mutations increase selectivity for target T cells and minimize potential toxicity associated with systemic IL2–driven activation of other IL2R–expressing cells, as previously described^9^ (Fig. 3A).

An IST with HLA-A201: cytomegalovirus pp65_495-502_ peptide (CMV) induces expansion of antigen-specific T cells from PBMCs^10^. To investigate IST-associated IS, we performed imaging flow cytometry. PBMCs spiked with CMV-specific RA14 TCR-expressing CD8^+^ T cells were incubated with or without CMV(CueIL2) IST. Next, PBMC samples were stained with antibodies to identify CD4^+^ and CD8^+^ T cells, NK cells, B cells and monocytes and subjected to imaging flow cytometry (Fig. 3A–F and Supplementary Fig. 5). Treatment with CMV(CueIL2) IST increased the frequency of doublet events overall (Supplementary Fig. 5B). CD3^+^CD8^+^ T cell – CD14^+^ monocyte doublets were observed in the absence or presence of the specific IST (Fig. 3B). However, the area of contact between the doublets was increased in the presence of antigen-specific ISTs (Fig. 3C–E), and the ISTs and CD3 accumulated in monocytes interacting with CD8^+^ T cells (Fig. 3C, D, F). Based on prior observations that the attenuated Fc in IST’s still bound to FcγRI^9^, we hypothesized that the monocytes may present the IST to T cells using FcγRI. To test this hypothesis, the imaging flow cytometry analysis was repeated in the presence of CMV(CueIL2) IST with or without a FcγRI neutralizing antibody. Blockade of the FcγRI significantly reduced the ability of IST to induce CD3^+^CD8^+^ T cells–CD14^+^ monocyte IS, as shown by the reduced contact interface (Fig. 3G).

IST-induced doublets also included CD3^+^CD8^+^ T cell homotypic conjugates. ISTs accumulated at the T-T cell contact, further confirming that these homotypic IS were triggered by the IST (Supplementary Fig. 5B–D). As T cells generally lack FcR, these T-T IS are likely mediated by trans engagement of the RA14 TCR on adjacent transduced T cells by the two HLA-A2-peptide complexes of the same IST (Supplementary Fig. 5E). Thus, we identified two modes of IST function: a classical T-APC IS mediated by FcγRI, and a T-T synapse between adjacent antigen-specific T cells. We chose to study the former as we have previously incorporated FcR into SLB to analyze this type of IS^38^, but we will come back to the potential relevance of both in the discussion.

To further investigate T-APC IS driven by ISTs, we modeled monocyte IST presentation to T cells via Fc receptors using FcγRI or a synthetic *Staphylococcus aureus* protein A-derived peptide (D-peptide) with human IgG binding activity, tethered to the SLB through a His-tag, all presented alongside recombinant ICAM-1, which is one of at least three LFA-1 ligands on human monocytes^39^ (Fig. 3H, I). An His-tagged FcγRII was used as a negative control for IST binding, and a MART1(CueIL2) IST was also studied to demonstrate the specificity of IS formation for the peptide loaded into the HLA-A2 of the IST. Only the CMV(CueIL2) IST presented by either FcγRI or the D-peptide, but not FcγRII, generated classical IS with an ICAM-1 ring and central IST cluster engaging the TCR^40,41^ (Fig. 3I). Given this functional similarity, follow-on studies utilized the chemically defined and inexpensive D-peptide to anchor the IST to SLB.

We next presented the ISTs alone on SLBs in order to evaluate if the attenuated IL2 in the IST is sufficient to mediate cell adhesion in the absence of ICAM-1^42^. To investigate this, we tested both CMV(CueIL2) IST and a CMV IST lacking CueIL2 molecules, without additional adhesion molecules on the SLB (Fig. 3J–L). Additionally, ISTs containing a MART1_26-35_ peptide, with and without CueIL2, served as antigen-negative controls. RA14 CD8^+^ T cell blasts adhered to SLBs presenting a high density of the CMV IST, with or without the presence of CueIL2. Furthermore, no adhesion was observed when the CMV peptide was replaced with the control peptide MART1, demonstrating that the avidity generated by IL2 receptor interactions with CueIL2 alone is not sufficient to mediate cellular adhesion, even at very high density (Fig. 3K). However, co-presentation of CueIL2 on the IST was associated with increased T cell-SLB contact size when the appropriate antigen was presented (Fig. 3K). The SLB-anchored ISTs were recruited to, and clustered to the center of, the cell-SLB contact (Fig. 3L), consistent with prior observations of TCR-pMHC interactions in SLB and supporting that the attenuated IL2-IL2 receptor interactions contribute to the overall avidity but do not promote receptor clustering or adhesion on their own. IST function was further validated by quantifying the proliferative response of RA14 CD8⁺ T cell blasts exposed to IST presented either through a solid-phase format or via a bead-SLB system. In both experimental setups, simultaneous presentation of antigen and CueIL2 (CMV–CueIL2) induced the strongest expansion of RA14 CD8⁺ T cells. In contrast, presentation of antigen alone or CueIL2 alone resulted in only minimal proliferative responses (Fig. 3M).

### Co-delivery of IL2 and antigen enhances IL2 receptor assembly and TCR complex proximity

**Fig. 4:**
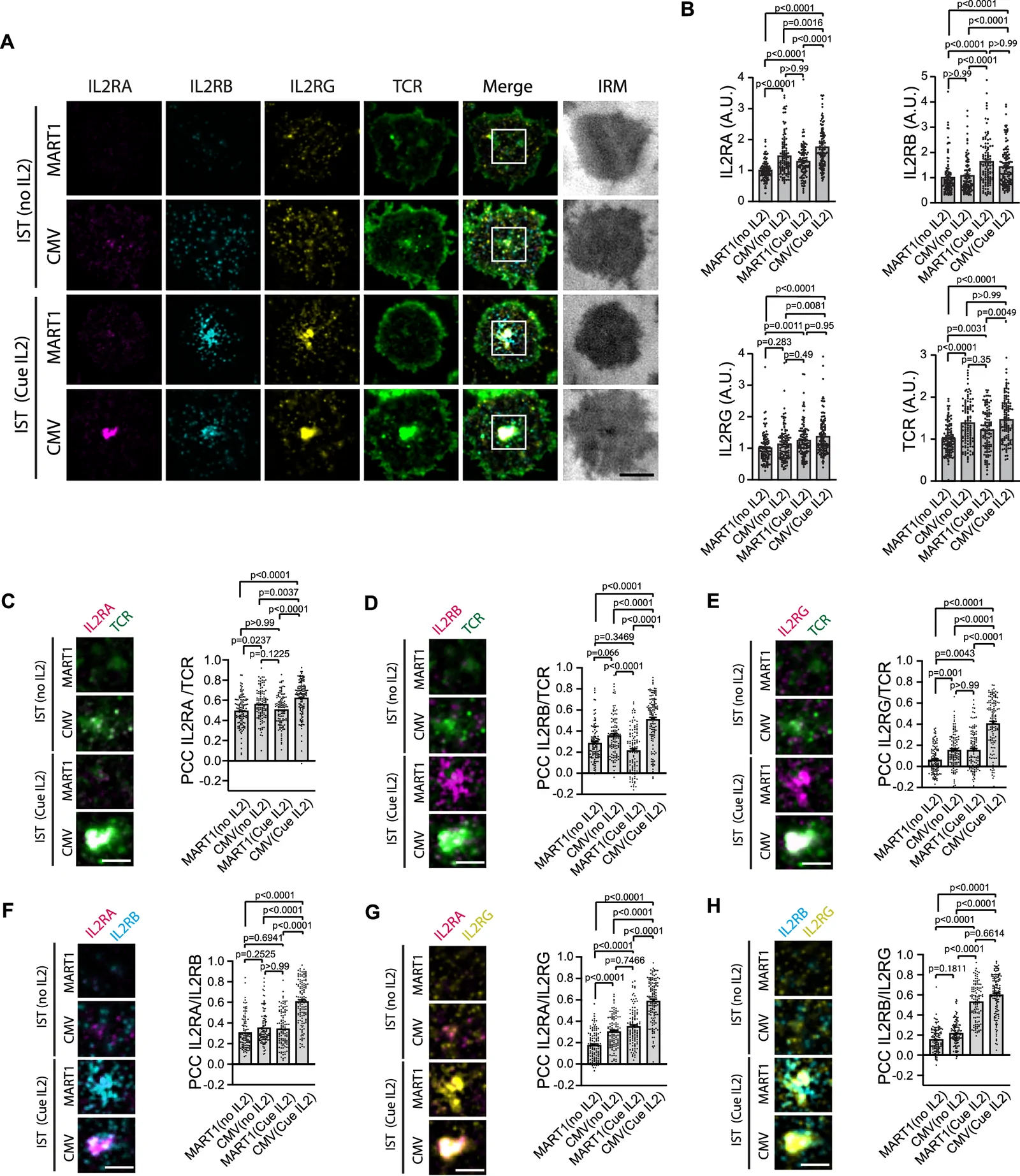
IST induce IL2 receptor assembly and proximity with the TCR at the IS. **A** Representative TIRF images of RA14 CD8^+^ blasts forming IS with SLBs containing the indicated IST with ICAM-1, CD80 and CD58. Cells were fixed after 15 min of SLB exposure and stained for the IL2 receptor subunits (IL2RA, magenta; IL2RB cyan; IL2RG yellow) and the TCR (CD3, green). Scale bar is 5 μm. **B** Quantification of the mean gray values for each protein (IL2RA, IL2RB, IL2RG and TCR) at the IS. Values were normalized to the negative control MART1 (noIL2). Quantification of the Pearson’s correlation coefficient (PCC) between the TCR and the IL2RA (**C**), IL2RB (**D**) and the IL2RG (**E**) and between the IL2 receptor subunits IL2RA and IL2RB (**F**), IL2RA and IL2RG (**G**) and the IL2RB and IL2RG (**H**). Left images show the zoomed in area for the square inset indicated in (**A**). Scale bar is 2.5 μm. Data are the mean ± SE, *n* = 114 (MART1(noIL2)), 110 (CMV(noIL2)), 111 (MART1(CueIL2)) and 127 (CMV(CueIL2)) cells, from three independent donors; Kruskal–Wallis test was used.

The recruitment and distribution of the IL2 receptor complexes and the TCR were visualized in RA14 CD8^+^ T cell blasts forming ISs with SLB containing the different IST constructs (MART1-noIL2; CMV-noIL2; MART1CueIL2; CMVCueIL2) with ICAM-1, CD80 and CD58. The greatest IL2 receptor subunit recruitment occurred with Ag-specific IST constructs containing CueIL2 (Fig. 4A, B). Quantification of subunit recruitment and co-localization revealed additional features of the IST system. Recruitment of IL2RB and IL2RG to the IS was driven by CueIL2, with a secondary contribution from the CMV-peptide. Conversely, recruitment of IL2RA and TCR was dominated by the CMV-peptide with a secondary contribution from CueIL2 (Fig. 4A, B). Surprisingly, CueIL2 increased IL2RA recruitment despite reduced binding affinity of the cytokine due to the F42A mutation. This was supported by increased co-localization of TCR with IL2RA (Fig. 4C), IL2RB (Fig. 4D), and IL2RG (Fig. 4E) in the presence of CMV(CueIL2). Furthermore, the combination of CMV-peptide and CueIL2 in ISTs enhanced co-localization of IL2RA with IL2RB (Fig. 4F) and IL2RG (Fig. 4G), while IL2RB and IL2RG co-localization increased in the presence of CueIL2 regardless of the peptide presented (Fig. 4H).

Presenting the CueIL2 and the CMV peptide-HLA as separate signals in two independent IST structures had little effect on the level of IL2R subunit recruitment but reduced the co-localization of IL2R subunits with each other and the TCR, compared to the full IST (Supplementary Fig. 6A). When the CueIL2 was substituted by nativeIL2 (with increased IL2RA and IL2RB binding) no major changes were observed in terms of receptor recruitment or assembly (Supplementary Fig. 7A–D). While the co-localization data do not demonstrate formation of molecular complexes required for signaling, proximity on this length scale (~300 nm) would be a prerequisite for such complexes. These results suggest that simultaneous presentation of IL2 and pMHC ligands at the IS potentiates IL2 receptor assembly and proximity to the TCR complex.

### Simultaneous delivery of IL2 and pMHC boosts IL2 receptor and TCR signaling

**Fig. 5:**
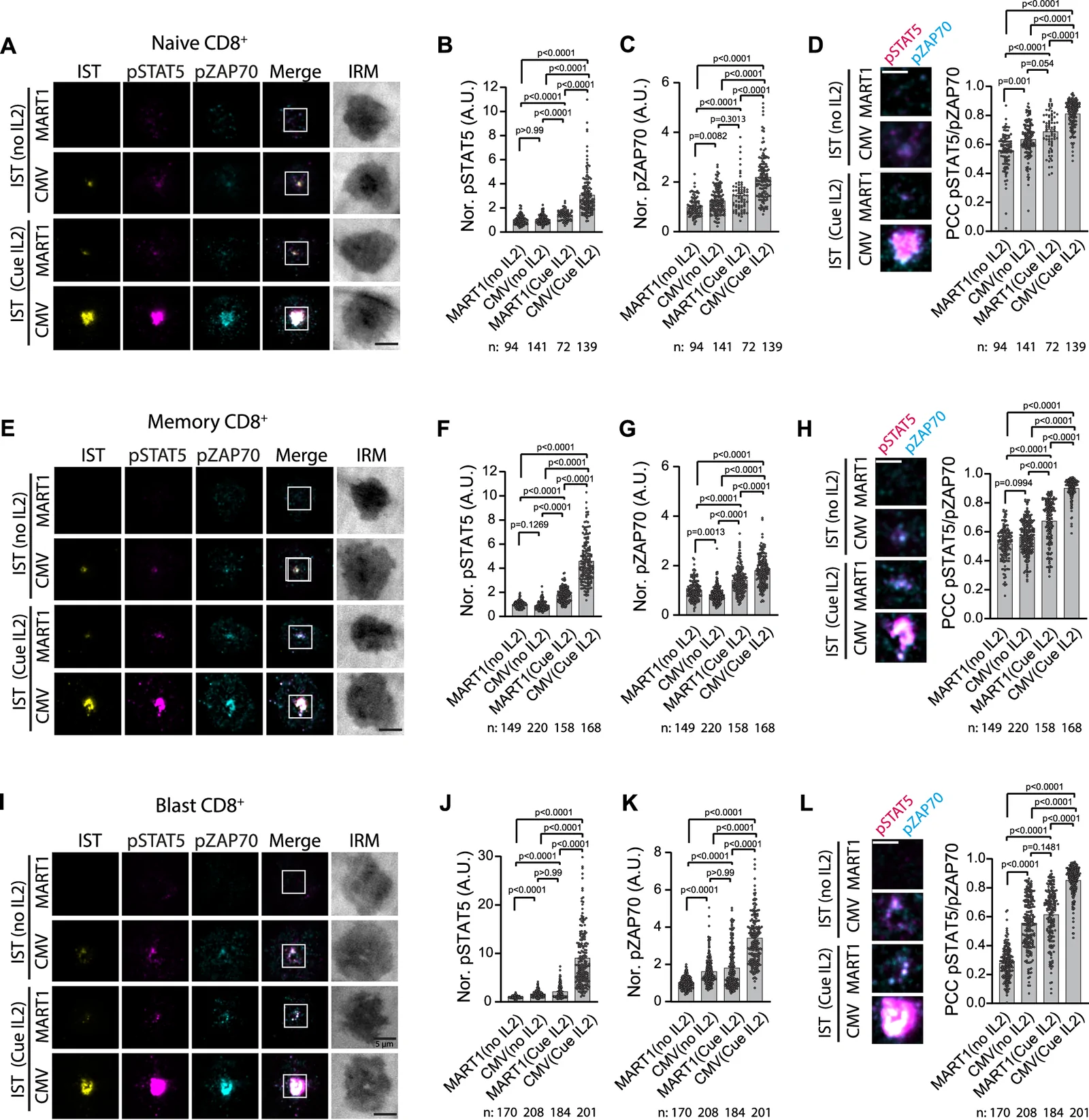
Simultaneous delivery of pMHC and IL2 signals promotes IL2 receptor assembly and TCR proximity to boost signaling. RA14 CD8^+^ Naive (**A**–**D**), Memory (**E**–**H**) and Blasts (**I**–**L**) T cells forming ISs with SLBs containing the indicated variant of IST AF488 (yellow), ICAM-1, CD80 and CD58. Cells were fixed after 15 min of SLB exposure and stained for pSTAT5 (magenta) and pZAP70 (cyan). Middle plots show the quantification of the mean gray values for pSTAT5 and pZAP70 at the IS for Naive (**B**, **C**), Memory (**F**, **G**) and Blast (**J**, **K**). Values were normalized to the negative control MART1(noIL2). Right plots show the Pearson’s correlation coefficient (PCC) between pSTAT5 and pZAP70 for Naive (**D**), Memory (**H**) and Blast (**L**). Images in **D**, **H** and **L** show the zoomed-in area for the square insets indicated in (**A**, **E**, **I**), respectively. Data are the mean ± SE. Cell numbers (*n*) for MART1(noIL2), CMV(noIL2), MART1(CueIL2) and CMV(CueIL2) were: naive, 94, 141, 72 and 139; memory, 149, 220, 158 and 168; and blast, 170, 208, 184 and 201, respectively; from three independent blood donors. Kruskal–Wallis test was used. CMV cognate antigen, MART1 negative control antigen. Scale bar is 5 μm for (**A**, **E**, **I**), and 2 μm for (**D**, **H**, **L**).

IL2 receptor signaling (pSTAT5) and TCR signaling (pZAP70) were visualized at the IS of RA14 naive, memory, and blasted CD8 + T cells exposed to SLB with the IST alongside ICAM-1, CD80 and CD58. In all T cell states, there was a significant and large increase in pSTAT5 and pZAP70 when the IST presented both the CMV-peptide and CueIL2 (Fig. 5). Surprisingly, the MART1-peptide with CueIL2 (MART1CueIL2) was often ranked second for both pSTAT5 and pZAP70, suggesting a synergy of the CueIL2 with the co-stimulatory signals on the SLB. Spatial correlation between pSTAT5 and pZAP70 was also highest with CMV CueIL2 IST, with MART1CueIL2 IST ranking second (Fig. 5D, H, L). This reciprocal synergy between IL2R and TCR signaling underscores the functional impact of ISTs on enhancing T cell activation.

When CueIL2 was presented on a separate molecule (CMVnoIL2 + FcCueIL2) pSTAT5 was unaffected, but pZAP70 was significantly lower compared to CMVCueIL2 IST, on which both CMV-peptide and CueIL2 were co-presented through the same molecule (Supplementary Fig. 6B). MART1(noIL2)+FcIL2(Cue) induced both pSTAT5 and pZAP70 signaling to levels similar to CMV(noIL2)+FcIL2(Cue), which were higher than the MART1(CueIL2) molecule. These results further indicate that the IL2 signal is sufficient to induce pZAP70 activation, in addition to pSTAT5. Furthermore, they also suggest that co-presentation of IL2 and peptide-HLA on the same molecule can limit IL2 action when the presented Ag is irrelevant to the T cell. No differences in pSTAT5 or pZAP70 were observed when the IST presented CueIL2 or a Native (wild-type) IL2 (Supplementary Fig. 7E–G).

## Discussion

IL2 receptor subunit expression varies across T cell subsets, fine-tuning IL2 sensitivity. Competing interleukin receptor subunits add regulatory complexity. We have confirmed expression levels of these receptor subunits in naive, memory and blasted CD8^+^ T cells and visualized their expression and distribution at the IS.

While polarization of IL2 release and signaling towards antigen-independent T-T cell contacts has been described^26,43,44^, none of these studies looked at the precise distribution of the IL2 receptor subunits in relation to the TCR clusters, resulting in an incomplete understanding of how IL2 signaling is organized during T cell activation. We found that IL2 receptor subunits display punctate patterns within the IS, and that these clusters can be configured homogenously across the interface, centralized in the IS or in the distal ring/corolla, likely reflecting different stages of dynamic recruitment and removal of the IL2 receptor to and from the IS, respectively. The central accumulation might be associated to increased cluster formation of IL2 receptors^45^, which likely involves their endocytosis or ectocytosis, as recently described for the TCR^36^, and was more prominent in the presence of IL2 signaling. Previous studies show that IL2/IL2 receptor complexes are rapidly internalized upon IL2 binding, down-regulating IL2 receptor signaling responses and IL2 availability. In a human IL2-dependent T lymphoma cell line, IL2 enters cells by endocytosis in a complex containing all subunits, but IL2RA is recycled back to the plasma membrane, while IL2RB and IL2RG are targeted to late endosomes/lysosomes and degraded^46,47^. Imaging of the IL2 receptor subunits by TIRFM revealed some puncta containing all subunits, but the majority were non-overlapping, suggesting that only a small percentage of the IL2 receptor subunits are forming a functional complex, even in the presence of IL2. This may be due to competition with other receptor subunits, such as the case described for the IL7R outcompeting the IL2RB/A for IL2RG at the IS of autophagy-deficient T cells^35^ and the ability of IL7 to suppress responses to IL4 and IL21 in naive T cells due to competition for limited IL2RG^19^.

IL2, IL2 receptor subunits and associated intracellular signaling components (JAKs and STATs) were also identified in EV released by CD8^+^ T cells. While IL2, IL2RA and IL2RB were enriched in EVs from activated CD8^+^ T cells, IL2RG was enriched in EVs from resting CD8^+^ T cells. EV were mostly single positive for IL2R subunits, suggesting sorting operates on individual subunits post-dissociation. Along these lines, IL2RG contains a PEST sequence that could represent one such sorting sequence^48^. EVs from activated T cells can induce pSTAT5 in naive CD8^+^ T cells, indicating a mode of T-T collaboration beyond soluble cytokines. EVs could act on neighboring cells through two main mechanisms: fusion with the plasma membrane^49^, leading to an immediate increase in surface expression of the transmembrane cargo (such as IL2 receptor subunits) and/or internalization^50^, where EV cargo would be incorporated into the recipient cell. In addition to these mechanisms, EVs may also enhance IL2 signaling by *trans*-presenting an IL2/IL2RA complex on their surface, similar to APC-mediated *trans*-presentation of IL2^51^ or IL15^52^.

In addition to characterizing the distribution of the IL2 receptor at the IS, we visualized its activity via STAT5 signaling. Blast cells showed the highest sensitivity to IL2, yet STAT5 signaling was evident at the IS across all T cell states. As previously described for T-T cell contacts, STAT5 signaling was polarized towards the synaptic cleft, mirroring the IL2 receptor complex^26^. Formation of the IS induced pSTAT5 in microclusters overlapping with TCR-mediated pZAP70 microclusters, indicating a spatiotemporal link between TCR and IL2 receptor signaling. TCR microclusters exclude CD45^31^, which is a promiscuous transmembrane tyrosine phosphatase that has been shown to target JAK kinases and inhibit cytokine receptor signaling^53^. This suggests that TCR engagement and IS formation can trigger pSTAT5 signaling. In fact, a crosstalk between the TCR and the IL2 receptor signaling pathways has been previously suggested^27,54^. The IS acts as the signaling platform where receptors and effectors are brought together in space and time, favoring the physical proximity between pathways and, therefore, enhancing any crosstalk between shared adapters and/or kinases to amplify signaling. The observed crosstalk between the IL2 and the TCR signaling pathways is supported by several previous mechanistic studies. On the one hand, the YxxL motif in the ITAM domains of CD3 chains is compatible with the STAT5-binding motif YxxL(I/V), suggesting a potential docking site for STAT5 in the TCR complex. Moreover, studies have shown that both STAT5 and JAK3 can associate with the TCR complex in a LCK-dependent manner^55,56^. In a recent study, we have demonstrated the LCK-dependent activation of another STAT family member, STAT3, at the synapse of CD4^+^ T^57^. On the other hand, LCK has been shown to interact with the IL2RB via specific sites on its catalytic domain, and IL2 can stimulate LCK activation in T lymphocytes^58,59^. These interactions offer a plausible mechanistic explanation for the TCR/IL2R synergistic crosstalk described here.

To further explore the spatiotemporal relationship between the IL2 and TCR pathways and the therapeutic relevance of simultaneously delivering both signals, we visualized and analyzed ISs induced by ISTs. Image stream experiments under conditions leading to expansion of specific T cells in PBMCs revealed presentation of the IST by monocytes. T cell-monocyte contacts were enriched in the presence of the IST, as suggested by a larger membrane contact between the two cells and the engulfment of the IST together with CD3 molecules by monocytes in contact with CD8^+^ T cells. T cell stimulation can lead to budding of TCR toward the APC by ectocytosis in both CD4^+^ and CD8^+^ T cells^36^, which would account for the appearance of IST and CD3 in the monocytes. Fc mutations (L234A; L235A) in ISTs attenuate, but do not eliminate, common fragment receptor (FcγRI) binding of the IST. The affinity of FcγRI for the mutated Fc in ISTs is similar to the affinity of FcγRII for wild-type human IgG^9^, which is functional in synapses^60^. While monocytes are the major FcγRI ^+^ APC in the blood, the DC subset 5 (DC5) also expresses FcγRI and is able to prime T cells in lymphoid tissues and the tumor microenvironment^61^. This mechanism may enhance activation of rare antigen-specific T cells by IST. At the lymph nodes, CD8^+^ T cell activation occurs in two phases: an initial priming by antigen-presenting DC followed by long-term re-engagement with DC in specific subfollicular niches. There, high-affinity CD8⁺ T cell clones receive paracrine IL2 from CD4⁺ T cells, supporting their proliferation and differentiation^62^. By delivering both antigen and IL2 in a single molecular complex, ISTs have the potential to merge these two phases into a single step, driving more rapid and efficient expansion of tumor-specific CD8⁺ T cell clones. We also observed that IST can induce T-T synapses, which requires that both T cells are specific for the same pMHC. Such encounters would be more frequent after expansion of antigen-specific T cells following the T-APC IS. The T-T synapses may play a role in further T cell expansion driven by antigen and IL2, forming a bridge between T cells.

As demonstrated in the SLB system, ISTs simultaneously recruited and activated the TCR and the IL2 receptor subunits at the IS. Remarkably, Ag presentation was enough to trigger the assembly of functional high-affinity IL2 receptors and IL2 presentation boosted TCR signaling to the IS, even in the absence of Ag. Additionally, TCR engagement increased the proximity between the IL2 receptor and the TCR complex itself, further suggesting that the synergy between these pathways relies on their spatial proximity, thereby explaining the maximal activity achieved by ISTs where these signals are covalently linked. This suggests that IST act in *cis* mode, meaning that the same IST molecule recruits both IL2 receptor and TCR, rather than a scenario where one IST molecule binds solely to either the TCR or the IL2 receptor within the same cell. Indeed, recent studies highlight the importance of *cis*-targeted IL2 variants in enhancing T cell immunotherapy, although in these instances PD-1 or CD8 receptors were used to localize IL2 to T cells^22,23,63^. In contrast, *cis*-targeting of IL2 through TCR specificity offers superior selectivity, as it selectively engages disease-relevant T cells. In cancer patients, most T cells expressing PD-1 or CD8 are likely not specific to tumor antigens. Importantly, this synergistic effect was largely preserved despite attenuation of IL2 binding affinity, indicating that the functional outcome depends more on spatial co-localization than on maximal cytokine potency. These findings support a model in which ISTs are engineered to couple signal 1 (pMHC–TCR) with signal 3 (IL2) in a single molecule, thereby promoting a localized and self-reinforcing signaling cascade. Within this framework, IL2 attenuation serves primarily to improve safety and selectivity, while preserving the *cis*-driven synergy that underpins therapeutic activity. Together, this highlights a key design principle of ISTs: the integration of antigen specificity with spatially constrained cytokine signaling to selectively amplify responses in tumor-specific T cells.

The synaptic synergy observed between IL2 and the TCR may extend to other common γ-chain cytokines. Although cytokines such as IL7, IL15 and IL21 share IL2RG, differences in receptor composition and STAT signaling bias could result in related but functionally distinct effects at the IS. Thus, other γc-family cytokines may similarly engage spatially coordinated crosstalk with the TCR while shaping T cell differentiation in unique ways. For example, could IL7 survival signals be integrated with self TCR-pMHC recognition in naive T cells that depend upon these signals^64^.

In vivo immune activation through cytokines or surface receptors in response to antigenic challenge is a highly regulated process. Our data provide key insights into the interplay between TCR and IL2 signaling at the IS. The extension of this biology via ISTs further highlights the importance of TCR engagement in the context of IL2 signals. Harnessing the TCR specificity is an elegant approach to selectively activate disease-specific T cells, which allows for a natural mechanism for the generation of a therapeutic index for IL2. This contrasts with other approaches where different variants of IL2 are being pursued with little to no consideration towards disease-specific T cells. An extension of the learnings here could be deployed to further our understanding of additional T cell-activating cytokines (IL7, IL15, IL21, etc.) and cell surface receptors (CD28, 41BB, etc). To note, many prior clinical approaches in oncology have attempted to harness these potent activation pathways by deploying systemic cytokine administration and/or receptor-agonist antibodies. Most of such studies have had challenges with poor tolerability, toxicities and minimal clinical benefit. Our mechanistic data with ISTs may provide an avenue to selectively drug the anti-tumor T cell repertoire to enhance clinical benefit while preserving patient safety. Indeed, to this end, the emerging clinical data with CUE-101 and CUE-102, which are two different ISTs currently in clinical development of solid cancers, exhibit preliminary evidence of clinical benefit and favorable tolerability^11,12^.

In conclusion, our work demonstrates that cis-targeting attenuated IL2 and pMHC molecules, not only effectively overcomes the challenge of selectively delivering IL2 to antigen-specific T cells, but also represents a strategy to amplify the inherent synergy between these signaling pathways at the single-cell level. This discovery represents a promising leap forward in precision immunotherapy, with far-reaching implications for targeted cancer treatment.

## Methods

### Isolation of primary human CD8^+^ T cells

Primary human T cells were isolated from leukoreduction system (LRS) chambers, which were supplied by the UK National Health Service Blood and Transplant and sourced from healthy, anonymous donors, under written consent. The use of these chambers was authorized by the NHS Health Research Authority, National Research Ethics Service (REC reference 11/H0711/7). Information on donor sex and gender was not available, as samples were fully anonymized prior to acquisition; accordingly, these variables were not included in the study design. Total CD8^+^ T cells were purified using the negative selection kits RosetteSep Human CD8^+^ T Cell Enrichment Cocktail (StemCell Technologies, #15021). To obtain primary naive T cells (CD8⁺CD45RA⁺CCR7⁺CD45RO⁻CD57⁻CD56⁻) or memory T cells (CD8⁺CD45RA⁻CD45RO⁺), enriched CD8^+^ T cells were further purified using EasySep Human Naive CD8^+^ T Cell or EasySep Human Memory CD8^+^ T Cell Isolation Kits (StemCell Technologies, #19258 and #19159, respectively). Cells were cultured at 37 °C, 5% CO_2_, in RPMI 1640 medium (Life Technologies, #31870-025) supplemented with 10% FCS, 4 mM L-glutamine, 1% Penicillin-Streptomycin (Gibco), 1% Non-Essential Amino Acids Solution (Thermo Fisher Scientific, #11140-050), 10 mM HEPES (Life Technologies, #15630-056). Naive and Memory CD8^+^ T cells were used without any additional pre-activation step. To generate blast CD8^+^ T cells, total CD8^+^ T cells were cultured in the presence of anti-human CD3/CD28 Dynabeads (Gibco, #11132D) at a ratio of one bead per cell and in the presence of 50 U/ml of IL2. Dynabeads were removed by magnetic separation after 3 days, and cells were kept in culture in the presence of IL2 until day 7.

### Expression plasmids and mRNA electroporation

The sequences for the CMV ppp65-specific *RA14 TCR* were provided by Cue Biopharma. *RA14 TCRA* and *TCRB* constructs were synthesized using GeneArt services from Thermo Fisher and subcloned into a pGEM-GFP-64A plasmid between HindIII and KpnI. A Kozak sequence was added, and codon optimization for human expression was applied. Both *RA14 TCRA* and *TCRB* constructs included a cysteine modification, as previously described in ref. ^65^. For mRNA synthesis, plasmids were linearized and in vitro RNA transcription and in vitro poly-adenylation were performed using the mMESSAGE mMACHINE T7 ULTRA Transcription kit (Thermo Fisher Scientific, #AMB1345-5), following manufacturer’s instructions. For electroporation, cells were washed three times in Opti-MEM media (LifeTechnologies, #31985-047) and resuspended at 2–2.5 × 10^6^ cells in 100 μl and mixed with 5 µg of each mRNA stock (RA14 TCRA, RA14TCRB and CD3ζ). Samples were then transferred to a Gene Pulser/Micropulser electroporation cuvette, 0.2 cm gap (BIO-RAD, #45-0125) and pulsed at 300 V for 2 ms in an ECM 830 Square Wave Electroporation System (BTX). Cells were immediately transferred to supplemented RPMI and cultured at 37 °C, 5% CO_2_ before usage after 18–24 h. Blast CD8^+^ T cells were maintained in the presence of 25 U/ml of IL2, whereas no IL2 was added to the culture medium of electroporated Naive or Memory T cells. On the day of the experiment, cells were washed and resuspended in fresh medium with or without IL2, as specified for each condition and time point. Transfection was confirmed by flow cytometry using cognate tetramer staining.

### Preparation of supported lipid bilayer and immunocytochemistry

To prepare support lipid bilayer (SLB), glass coverslips (Nexterion, #1472315) were plasma cleaned and mounted onto six-channel chambers (Ibidi, #80608). Small unilamellar liposomes were prepared by extrusion using 100 mol% 1,2-dioleoyl-sn-glycero-3-phosphocholine (Avanti Polar Lipids Inc., #4235-95-4) or 75% 1,2-dioleoyl-sn-glycero-3-phosphocholine and 25% 1,2-dioleoyl-sn-glycero-3-[(N-(5-amino-1-carboxypentyl) iminodiacetic acid) succinyl]-Ni (Avanti Polar Lipids Inc., #790404 C) at a final lipid concentration of 0.4 mM. Channels in Ibidi chamber were covered with a 1:1 mixture of these two liposome preparations, blocked and washed. SLBs were then incubated with the indicated mix of His-tagged proteins to achieve the desired density of molecules on the SLB: ICAM-1 (200 molecules/μm^2^), CD80 (200 molecules/μm^2^), CD58 (200 molecules/μm^2^), CMV pp65-HLA-A2 (100 molecules/μm^2^). When indicated, UCHT1 (30 molecules/μm^2^) was used instead of peptide HLA-A2 complexes. His-tagged proteins were produced in-house and, when required, conjugated with the AlexaFluor (AF) dyes of interest (405, 488, 568, or 647). For experiments with ISTs, SLBs were incubated with 200 nM of His-tag D-Peptide derived from Protein A (Bankpeptide, Hefei, China)^66^ for 20 min and then the protein mix was added with the indicated IST variant and FcCueIL2 at 30 nM. When indicated, in-house purified His-tagged FcRγI (CD64) or FcRγIIA (CD32A) at 200 nM were used instead of D-peptide. Unless otherwise specified, SLB experiments with IST also included ICAM-1, CD80 and CD58 His-tagged proteins. To generate the ISs, cells were exposed to the bilayers at 37 °C for the indicated amount of time, fixed with 4% PFA and washed. For immunocytochemistry, cells were permeabilized, blocked and incubated with the primary antibody of interest (anti-pSTAT5 AF647, BD Biosciences AB_399882 at a 1:5 dilution; anti-pZAP70 CST at a 1:100 dilution; anti-pZAP70 PE BD Biosciences AB_396919 at a 1:5 dilution; anti-IL2RA FITC clone 2A3 BD Biosciences AB_400334 at a 1:5 dilution, anti-IL2RB APC clone Mik-β3, BD Biosciences AB_2869796 at a 1:5 dilution; anti-IL2RG, Thermo Fisher AB_2856707 at a 1:100 dilution; anti-CD3 PE, BioLegend AB_2562047 at a 1:100 dilution) overnight at 4 °C. When required, samples were further incubated with an AF-conjugated secondary antibody for 1 h at RT used at a 1:200 dilution.

### TIRFM, Airyscan confocal microscopy, Opera Phoenix high content imaging and image analysis

TIRF imaging was performed on an Olympus IX83 inverted microscope with a Mitico TIRF module. The instrument was equipped with an Olympus UAPON 150x TIRF 1.45 NA objective, 405 nm, 488 nm, 568 nm and 640 nm laser lines and Photomertrics Evolve delta EMCCD camera. Some experiments were performed with an Olympus UPLAPO 100x OHR 1.5 NA ojective and a Photometrics Prime 95B sCMOS camera. Results from the two systems were equivalent except that the Prime 95B provided >3-fold larger field of view. Interference reflection microscopy (IRM) was used to visualize the contact between the T cell and the SLB. For live imaging experiments, SLBs were transferred to a pre-heated incubator on top of the TIRF microscope and cells were added in the well. To minimize imaging bias during TIRF acquisition, the field of view was selected and focused based solely on the IRM channel, which enabled unbiased identification of cell–bilayer contacts prior to examining fluorescent channels. Data on the uniformity of TIRF illumination as a basis for quantification of fluorescence signals within and across experiments is available^67^. The error across the field was within 20% and difference in single molecule fluorescence values across months were not statistically significant. Airyscan imaging was performed using a Zeiss 980 LSM using a 63x oil 1.40 NA objective. Images were visualized using ZenBlue v3.3.89.0000 Software or Fiji v2.14.0 (ImageJ). For High Throughput Imaging, SLBs were prepared on 96-well plates and imaged with the opera Phoenix High Content Imaging System. Imaging was performed at RT. Image analysis was performed using Fiji v2.14.0 (ImageJ), RRID:SCR_002285. Pearson correlation coefficients (PCC) were calculated using the JACoP plugin. To quantify the mean gray value per cell, synaptic contacts were segmented using the IRM image. To compare IST mean gray values, a correction factor was applied to the image to account for the different fluorophore-to-protein ratio of each IST variant.

### Flow cytometry

To assess levels of intracellular phosphorylated proteins (pSTAT5), cells were treated with 50 U/ml of IL2 for 15 min at 37 °C. When indicated, cells were treated with IL2 neutralizing antibodies at 20 µg/ml (clone MQ1-17H12, Biolegend AB_315088) or isotype control, as previously described^68^. Next, cells were fixed in 2% PFA for 15 min, washed and gently resuspended in ice-cold 90% methanol and incubated for a minimum of 3 h at −20 °C. Next, cells were washed and incubated with the corresponding antibody (anti-pSTAT5 AF647, BD Biosciences AB_399882) in 0.5% BSA PBS solution for 1 h, washed again, and analyzed. To confirm RA14 TCR electroporation, cells were stained with MHC tetramer (Tetramer/APC – HLA-A*02:01 CMV pp65 [NLVPMVATV]; MBL) for 30 min at 4 °C, washed thoroughly, and fixed with 0.5% PFA in PBS, according to the manufacturer’s instructions. For staining of CD25 and naïve and memory markers, cells were fixed in 4% PFA for 15 min, washed and incubated with the corresponding antibody (anti-CD25 AF488, clone BC96, Biolegend 302615; anti-CD45RA APC/Cy7, clone HI100, Biolegend 304127; anti-CD45RO BV421, clone UCHL1, Biolegend 304223) in 0.5% BSA PBS solution for 1 h at a 1:20 dilution, washed again, and analyzed. Samples were acquired using the LSR II Flow Cytometer (BD Biosciences). Data were analyzed using FlowJo (version 10.7.1, RRID:SCR_008520).

### Proliferation assay on beads-SLB and solid phase substrate

CD8^+^ T cells were labeled with 0.5 μM CellTrace CFSE (Thermo Fisher Scientific, #C34554) at room temperature for 5 min and washed twice with complete medium. Bead-supported lipid bilayers (BSLB) were prepared as previously described^69^. Briefly, silica beads (5.0 µm diameter, Bangs Laboratories, Inc., #SS05003) were washed with PBS and incubated with a 1:1 mixture of small unilamellar liposomes composed of 100 mol% 1,2-dioleoyl-sn-glycero-3-phosphocholine (Avanti Polar Lipids Inc., #4235-95-4) and 25% 1,2-dioleoyl-sn-glycero-3-[(N-(5-amino-1-carboxypentyl) iminodiacetic acid)succinyl]-Ni (Avanti Polar Lipids Inc., #790404 C), prepared by extrusion at a final lipid concentration of 0.4 mM. Beads were blocked, washed, and incubated with 200 nM of His-tagged D-Peptide derived from Protein A (Bankpeptide, Hefei, China) for 20 min, followed by incubation with each IST variant at 100 nM for an additional 20 min. Beads were then washed and incubated with CFSE-stained cells at 37 °C and 5% CO_2_ for 96 h. For solid-phase substrate experiments, polymer µ-Slide 18-Well chambered coverslips (Ibidi) were coated with poly-L-lysine (Sigma-Aldrich, #P8920) and incubated with 1 μg/mL ICAM-1 and 1 μg/mL FcγRI at 37 °C for 3 h. Coverslips were washed 3 times and incubated with 100 nM of each IST at room temperature for 20 min. After 3 washes, CFSE-labeled cells were incubated at 37 °C and 5% CO_2_ for 96 h. Finally, cells were recovered using ice-cold PBS, fixed with 4% PFA, and acquired using a Northern Lights spectral flow cytometer (Cytek Biosciences). Data were analyzed using FlowJo (version 10.7.1, RRID:SCR_008520).

### Single-cell RNA sequencing

The raw scRNA-seq data was downloaded from Gene Expression omnibus (GEO) under accession number GSE126030. The article corresponding to the repository illustrates that all the T cells were obtained from human PBMCs. These T cells were activated in the presence of anti-CD3/anti-CD28 beads for 48 h and compared to the resting T cells which were cultured in media alone. The fastq files obtained from GEO were processed using the Cell Ranger (v3.1.0) counts pipeline. The sequencing reads were aligned to Human GRCh38-3.0.0 and the cell count matrices were obtained. These filtered gene–cell matrices were then read into the Seurat v4.0 pipeline implemented in R for downstream analysis. We followed the standard Seurat pre-processing workflow with QC-based cell filtration, data normalization and scaling. We further identify major cell types with highly variable genes and performed Jackstraw analysis to isolate significant principal components. The UMAP embeddings were then computed using the isolated PCs and known marker genes were used to characterize cell types.

### Isolation and purification of extracellular vesicles and proteomic analysis

Cells were incubated in serum-free medium for 48 h followed by collection of the medium. The media supernatant was spun at 300 × *g* to remove cells, followed by a 4000 × *g* centrifugation to discard cell debris. The supernatant was concentrated using 100 kDa molecular weight cut-off Amicon centrifugal filter units (Merck Millipore, Billerica, Massachusetts, USA) to 2 ml and subsequently loaded on a Sepharose 4 Fast Flow resin column (10 mm × 300 mm; GE Healthcare, Little Chalfont, UK), connected to the ÄKTA pure system (GE Healthcare) and eluted at 0.5 ml/min flow rate using PBS as the eluent. Chromatogram was recorded using absorbance at 280 nm. A total of 0.5 ml fractions were collected, and EV-containing fractions were pooled and concentrated to 2 ml with 10 kDa molecular weight cut-off Amicon centrifugal filter units (Merck Millipore). For the proteomic analysis, EV samples (750 μL) were reduced with 5 μL of 10 mM tris(2-carboxyethyl)phosphine and alkylated with 50 mM of iodoacetamide for 30 min each, then acidified with 12% phosphoric acid 10:1 vol:vol, and then transferred to S-trap columns. Then, samples were precipitated using 1:8 vol:vol dilution of each sample in 90% methanol in 100 mM Triethylammonium bicarbonate and digested with trypsin (Promega, #V1115) overnight at 37 °C. The samples were then run on a LC-MS system comprised of an Evosep One and Bruker timsTOF Pro. For proteomic analyses the raw files were searched against the reviewed Uniprot Homo sapiens database (retrieved 2,01,80,131) using MaxQuant version 1.6.10.43. The intensities were baselined using NTA concentration and average-based normalization was carried out to obtain the enriched set of proteins in each EV sample.

### Nano flow cytometry

The Flow NanoAnalyzer (NanoFCM Co., LTD) was used to perform Nano flow cytometry analysis according to the manufacturer’s instructions. To construct a calibration curve and enable the conversion of side scatter intensities to particle size, a Silica Nanospheres Cocktail (S16MExo, NanoFCM) was employed as the size standard. In addition, a concentration standard (200 nm PS QC beads, NanoFCM) was used to calculate particle concentrations. A 488 nm laser at 25/40 mW, 10% ss decay was utilized, and the detectors were equipped with 525/40 (AF488) and 580/40 (PE) bandpass filters. Antibodies were allowed to bind for 30 min on ice, followed by removal of unbound antibodies through centrifugation for 1 h at 100,000 × *g* at 4 °C. Labeled vesicles were then resuspended in 50 µL of PBS. Sample and label-specific signals were defined using buffer alone (PBS), unstained samples, samples stained with isotype controls, and auto-thresholding.

### Imaging flow cytometry (ImageStream)

CD8^+^ T cells were purified from a PBMC sample. Some PBMCs were kept frozen while purified CD8^+^ T cells were blasted as usual. On day 7, blast CD8^+^ T cells were electroporated with the RA14 TCR mRNA 24 h prior to co-culture experiment. For co-culture, 2 million electroporated RA14 CD8^+^ T cells were added on 4 million of freshly thawed PBMCs from the same donor and incubated for 2 h at 37 °C in a small volume to favor cell contacts. When indicated, CMV (CueIL2) IST-AF488 1 µM was added to the media. When indicated, a CD64 (FcγRI) neutralizing antibody was added (50 µg/ml, clone 10.1 Biolegend AB_314486), as previously described^70^. Next, cells were fixed with 2% PFA for 10 min, washed and blocked with TrueStain buffer (Biolegend) for 10 min. Finally, samples were stained with the antibody cocktail for ImageStream analysis (UCHT1-PE, Biolegend AB_2562047; CD19-AF594, Biolegend AB_2563233; CD14-BV421, Biolegend AB_2561342; CD8-AF700, Biolegend AB_2562789; CD4-APC/Fire750, Biolegend AB_2716182; CD56-APC/Fire750, Biolegend AB_2572104) using a 1:20 dilution for all antibodies except for UCHT1, which was used at a 1:100. Data were acquired on an ImageStream MK II (Amnis) with INSPIRE V4 software and analyzed using IDEAS 6.2 software. Briefly, in-focus images were used to gate doublets based on the area and the aspect ratio of the bright-field images. Subsequent gating was carried out using the specific marker staining. Doublets were further gated to identify possible CD8^+^ T cell conjugates with CD14^+^ monocytes (doublets double positive for CD8^+^ and CD14^+^ markers). Percentages of CD14^+^CD8^+^ doublets were quantified from the total focused events. High CD3^+^CD8^+^ cells were considered possible T-T cell doublets. For IS analysis, CD8, CD3 and CD14 markers were used to generate a mask for each cell type. Then, a new mask consisting of the overlapping area between the CD8 or CD3 and the CD14 channels was created for each image to identify the contact area between T cells and monocytes within the gated CD8+CD14+ population.

### Statistical analysis

All statistical tests were performed using GraphPad Prism 9 software, RRID:SCR_002798. Mann–Whitney test, Kruskal–Wallis test and paired *t*-test were used for statistical analysis and *p* < 0.05 was considered statistically significant.

### Reporting summary

Further information on research design is available in the Nature Portfolio Reporting Summary linked to this article.

## Supplementary information

Supplementary Information

Transparent Peer Review file

Reporting Summary

## Source data

Source data

## Data Availability

The mass spectrometry proteomics data generated in this study have been deposited in the PRIDE partner repository of the ProteomeXchange Consortium under accession code PXD070470. The raw scRNA-seq data were downloaded from the Gene Expression Omnibus (GEO) under accession number GSE126030. The imaging data are available under restricted access due to their large file size; access can be obtained by contacting the authors. All data are included in the Supplementary Information or available from the authors, as are unique reagents used in this Article. The raw numbers for charts and graphs are available in the Source Data file whenever possible Source data are provided with this paper.
