# Supplementary material for: Spatiotemporal co-regulation of IL2 and TCR receptors reveals early antigen-driven synaptic synergy in human CD8+ T cells
Source: Nat Commun. 2026 Oct 7;17:11431. doi: 10.1038/s41467-026-77110-1 (PMC13737045; doi:10.1038/s41467-026-77110-1)
Supplement: Supplementary file 1 [file 41467_2026_77110_MOESM1_ESM.pdf]

SUPPLEMENTARY INFORMATION

Spatiotemporal co-regulation of IL2 and TCR receptors reveals early antigen-driven synaptic synergy in human CD8$^+$ T cells

Jesusa Capera [1], María Navarro-Pérez [1], Ashwin K Jainarayanan [1], Salvatore Valvo [1], Lina Chen [1], Panyu Fei [1], Sarah Flannery [2], Roman Fischer [2], Saso Cemerski [3], Simon Low [3], Steven N Quayle [3], Raymond J Moniz [3], Anish Suri [3,*], Michael L Dustin [1,4*]

[1] The Kennedy Institute of Rheumatology, Nuffield Department of Orthopaedics, Rheumatology and Musculoskeletal Sciences, University of Oxford, Oxford, UK

[2] Target Discovery Institute, University of Oxford, Oxford, UK

[3] Cue Biopharma, Boston, MA, USA

[4] CAMS Oxford Institute, Chinese Academy of Medical Sciences & Peking Union Medical College, University of Oxford, Oxford, UK

* *Corresponding authors:*

Anish Suri:
Address: Cue Biopharma, 40 Guest Street, Boston, MA 02135, United States
Email: asuri@cuebio.com

Michael L Dustin:
Address: Kennedy Institute of Rheumatology, University of Oxford, Roosevelt Drive, Oxford OX3 7FY, United Kingdom
Email: michael.dustin@kennedy.ox.ac.uk
ORCID ID: 0000-0003-4983-6389

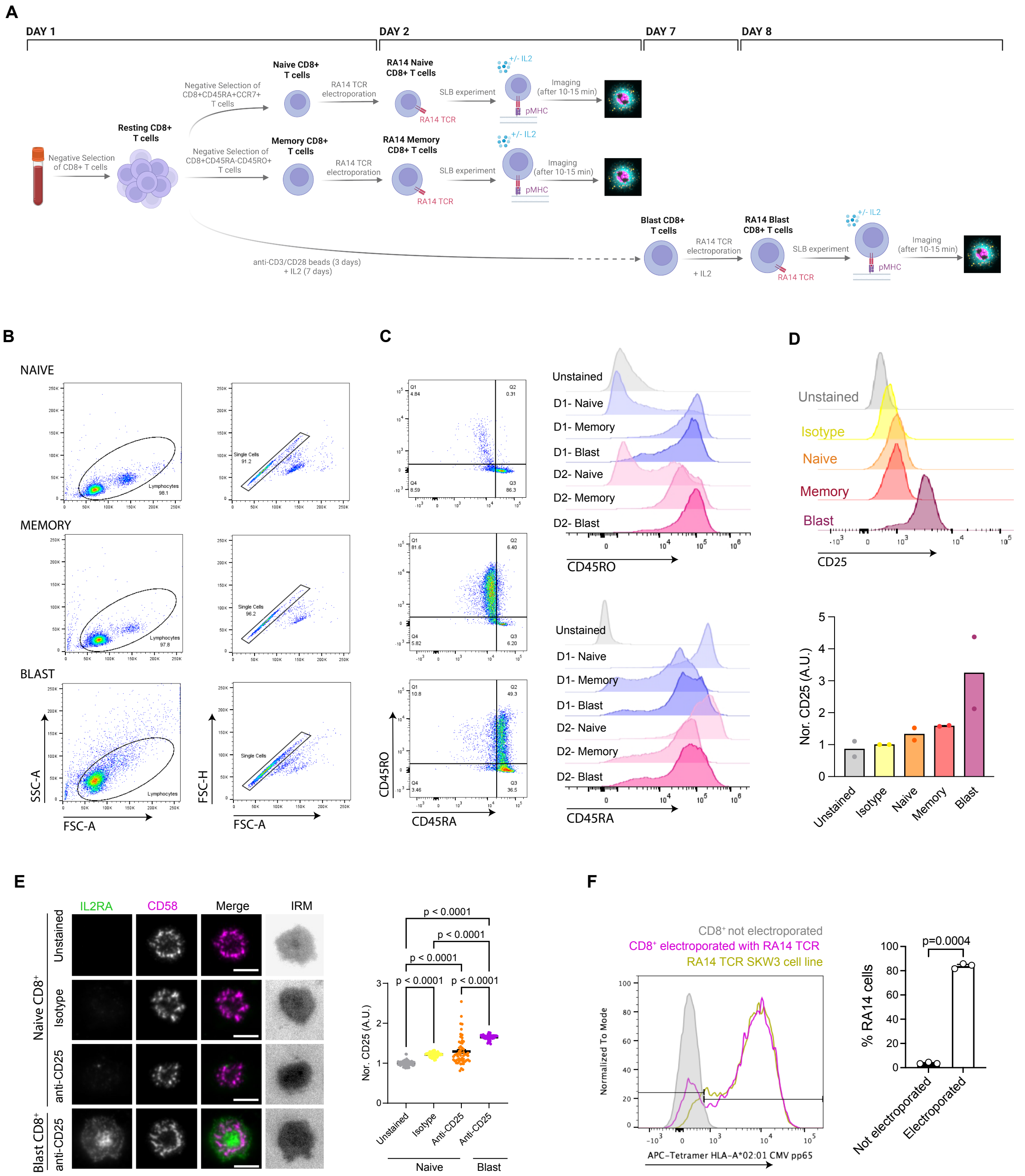

Supplementary figure 1: *Experimental workflow and phenotyping.* **(A)** Schematic diagram showing the experimental workflow used in this work. Created in BioRender. Capera, J. (2026) https://BioRender.com/tyi2aok. Briefly, total CD8$^+$ T cells were purified from leukoreduction system (LRS) chambers and further separated into Naive CD8$^+$ T cells (CD8$^+$CD45RA$^+$CCR7$^+$CD45RO$^-$CD57$^-$CD56$^-$) and Memory CD8$^+$ T cells (CD8$^+$CD45RA$^-$CD45RO$^+$) using immunomagnetic negative selection kits (STEMCELL Technologies). These subsets were then electroporated with RA14 TCR mRNAs and cultured without any pre-activation step (no cytokines or Dynabeads were added). Cells were used for experiments 18–24 h post-electroporation. For activated CD8$^+$ T cells (Blast), total CD8$^+$ cells were stimulated with anti-CD3/CD28 Dynabeads for 3 days and maintained with 50 U/ml IL-2 until day 7, when they were electroporated with RA14 mRNAs. These cells were likewise used 18–24 h post-electroporation. **(B)** Flow cytometry dot plots (from two

independent experiments with similar results) showing the gating strategy used to identify single lymphocytes in Supplementary figure 1C-F, Supplementary figure 3D and Figure 2A. **(C)** Left, CD45RA and CD45RO staining used to define the three $CD8^+$ T cell subsets. Right, histograms for CD45RA and CD45RO expression for two independent donors (D1, D2). **(D)** Representative histograms showing CD25 expression across the three $CD8^+$ T cell subsets (from two independent experiments with similar results), together with quantification of the median CD25 signal normalized to the corresponding isotype control. **(E)** Representative TIRF microscopy images of $CD8^+$ T cells fixed after 10 min of interaction with supported lipid bilayers (SLBs). IL2RA was immunolabeled using anti-CD25 AF488 (clone BC96). Mean fluorescence intensity was quantified and normalized to the average signal measured in the unstained (no antibody) control. Data is the mean +/- SE, n= 88 (unstained), 68 (isotype), 57 (anti-CD25 naive) and 52 (anti-CD25 blast); one-way ANOVA (F=170.1). Scale bar is 5 μm. **(F)** Representative histograms showing RA14 tetramer staining of $CD8^+$ T cells electroporated with RA14 TCR mRNAs or left non-electroporated. SKW3 cells transduced with RA14 TCR were used as positive controls. The plot shows quantification of the percentage of $RA14^+$ cells. Data is the mean +/- SE, n = 3 donors; paired t-test, t = 51.57, df = 2. Gating strategy as in (B).

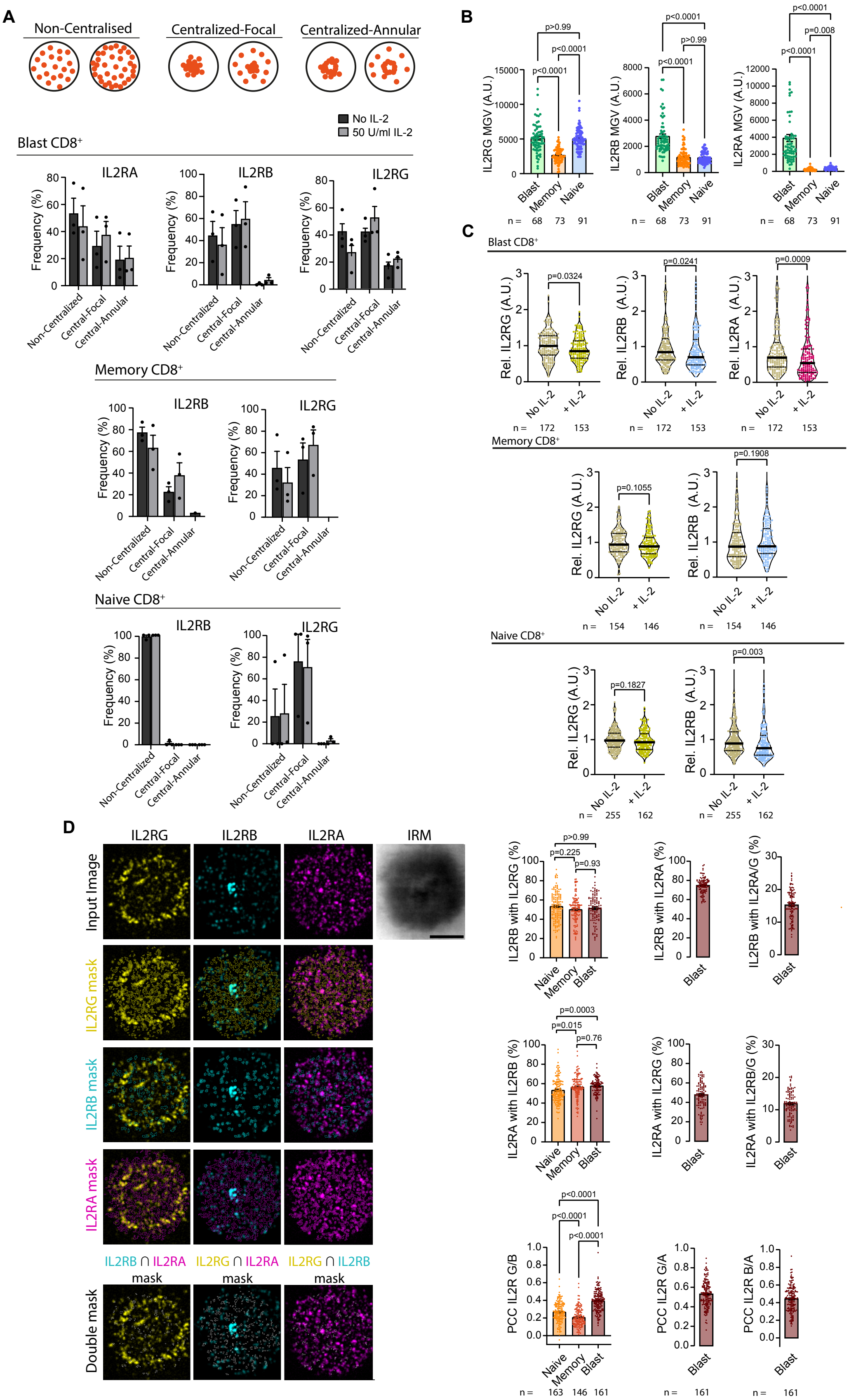

Supplementary figure 2: *Synaptic assembly of the IL2 receptor complex.* RA14 CD8+ T cells were exposed to Ag-specific SLBs in the presence or absence of 50 U/ml of IL2 to form ISs and fixed after 10 min. Samples were then permeabilized and immunostained against the IL2 receptor subunits. **(A)** Top,

schematic illustration of the classification criteria to classify the IL2 receptor distribution into non-centralized (left), centralized-focal (middle) or centralized-annular (right). Histograms show the frequency for each phenotype as quantified by visual inspection. Data is the mean +/- SE of three independent donors with >50 cells/donor. Data shown is an extension of the data in Fig. 1D-F. **(B)** Plots show the quantification of mean intensity for IL2RG, IL2RB and IL2RA at the IS in the presence of 50 U/ml of IL2. Histograms show the mean +/- SE; Kruskal-Wallis test. **(C)** Synapses were let to form by Blast, Memory or Naive RA14 $CD8^+$ T cells in the presence or absence of 50 U/ml of IL2 for 10 min before fixation. Violin plots show the quantification of the mean intensity values for each IL2 receptor subunit at the IS normalized to the condition without IL2 (No IL2). Data shows the median and quartiles, from 3 independent donors; Mann-Whitney test. **(D)** TIRF images of a RA14 $CD8^+$ blast T cell forming a synaptic contact with the SLB. Each IL2 receptor subunit image (input image) was segmented into a binary mask to detect the subunit clusters. Further masks were created from the intersection ($\cap$) between the masks of each pair of subunits (double mask). The interference reflection microscopy (IRM) image was used identify the whole cell contact. Next, the mean intensity value was measured within each mask. Top and middle plots show the percentage of the total IL2RB or IL2RA intensity (within the IRM area) that is within the other indicated IL2 receptor subunit or within the intersection between the two indicated masks. Bottom plots show the quantification of the Pearson's Correlation Coefficient (PCC) for each pair of IL2 receptor subunit at the IS. Histogram shows the mean +/- SE, from three independent donors; Kruskal-Wallis test. Scale bar is 5 µm.

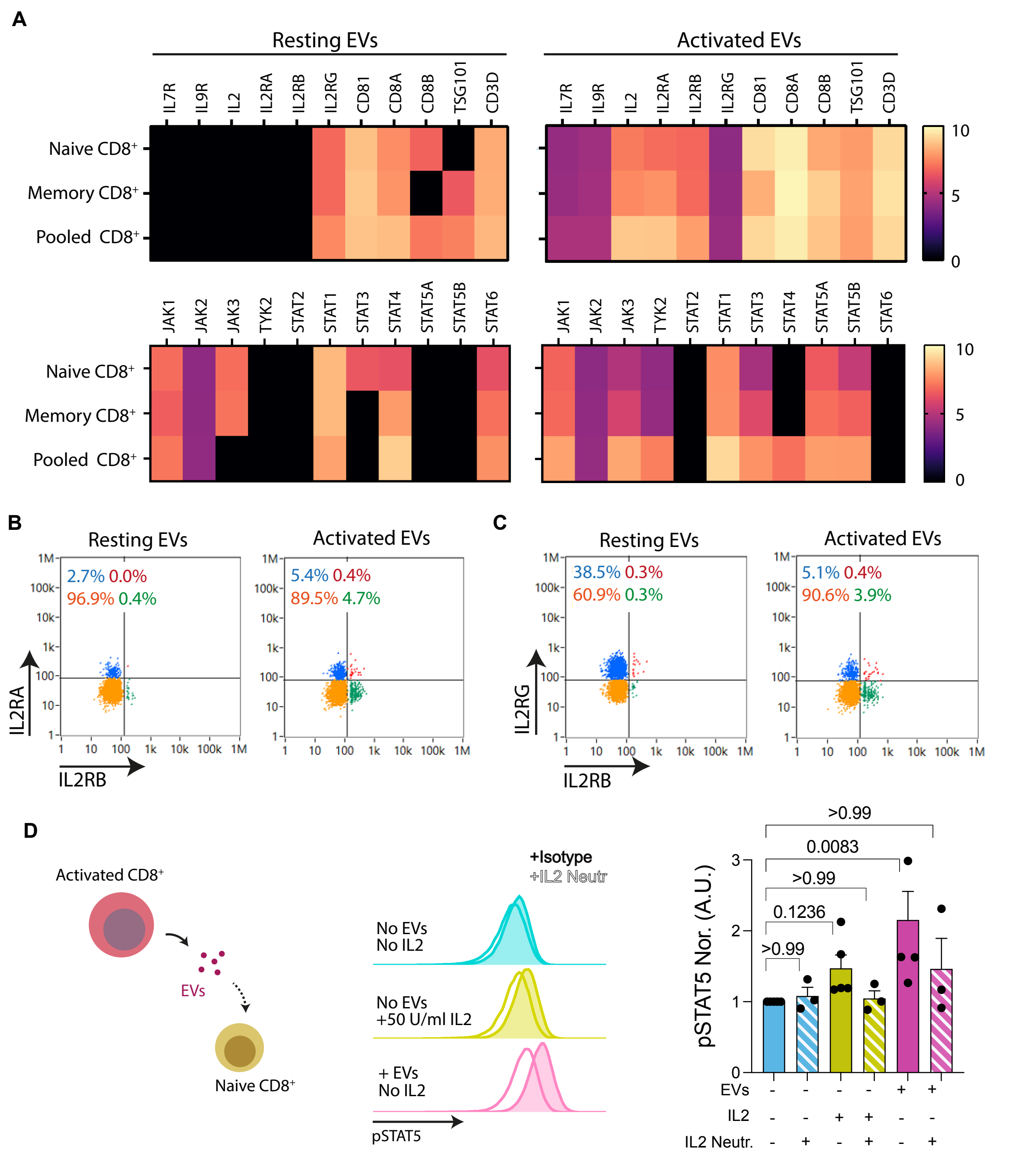

Supplementary figure 3: *Release of the IL2* receptor *subunits in EVs is dependent on the activation state of the CD8*$^+$ *T cells.* **(A)** Heatmap showing the normalized expression of proteins identified in EVs from resting and activated CD8$^+$ T cells (naive, memory and total/pooled CD8$^+$ differentiation states). All members of the common-gamma chain cytokine receptors and interleukins were interrogated, as well as JAK and STAT family proteins, together with other activation markers and EVs markers. The heatmap shows all species detected. For activation, cells were incubated with anti-CD3/CD28 Dynabeads for 48 hours. **(B)** Nano-FCM analysis showing the scatter plot for IL2RA (y-axis) and IL2RB (x-axis) positive EVs from resting and activated pooled CD8$^+$ T cell. Marked in red are the double positives (IL2RA and IL2RB), blue exclusively represents the IL2RA positive EVs (negative for IL2RB), green exclusively represents IL2RB positive EVs (negative for IL2RA), and the double negatives are marked in yellow. Similarly, **(C)** shows the scatter plot for IL2RG (y-axis) and IL2RB (x-axis) positive EVs from resting and activated CD8$^+$ T cell. Marked in red are the double positives (IL2RG and IL2RB), blue exclusively represents the IL2RG positive EVs (negative for IL2RB), green exclusively represents

IL2RB positive EVs (negative for IL2RA), and the double negatives are marked in yellow. **(D)** Naive $CD8^+$ T cells were exposed to EVs purified from activated $CD8^+$ T cells. After 18 hours with or without EVs incubation, Naive $CD8^+$ T cells were treated or not with 50 U/ml of IL2 for 15 min and analyzed by phospho-flow cytometry to quantify total pSTAT5 levels. An IL2 neutralizing antibody or isotype control was added 18 hours before the IL2 treatment. Histograms show representative pSTAT5 staining. Solid filled histograms show samples treated with IL2 neutralizing antibody and line-only histograms show samples treated with isotype controls. Right bar plots show the average median intensity for pSTAT5 normalized to the untreated control. Data represent mean ± SE; n=5 for isotype controls and n=3 for IL2–neutralized samples (independent blood donors), Krustal-Wallis test. Gating strategy as in Supplementary figure 1B.

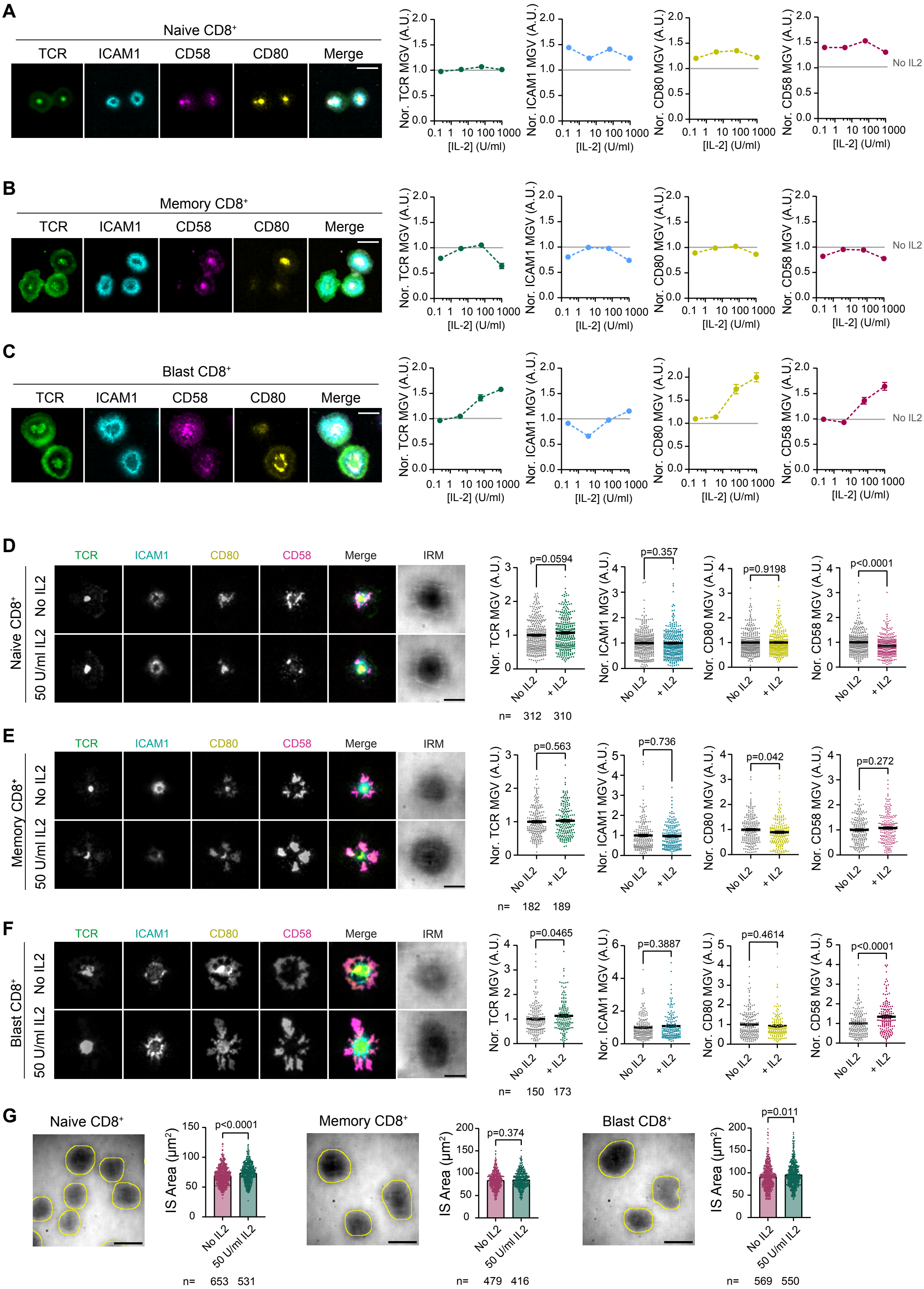

Supplementary figure 4: *IL2 effects on synapse formation.* Blast, Memory and Naive RA14 CD8$^+$ T cells were exposed to antigenic pMHC SLBs to form ISs. SLBs also contained His-tagged ICAM1-AF405, CD80-AF568, and CD58-AF647. When indicated, IL2 was simultaneously added. Samples were fixed

after 15 min of SLB and IL2 exposure and immunostained against the TCR (anti-CD3 AF488). **(A-C)** Samples were prepared in a 96-multi well plate containing the SLBs and incubated with increasing concentrations of IL2 in the media, simultaneously to SLB exposure. Synapses were then imaged using an Opera Phenix high content confocal system. Images on the left are representative examples. Bright field images (not shown) were used to segment the cells. Right plots show the mean intensities (MGV) for the TCR (green), ICAM1 (cyan), CD80 (yellow) and CD58 (magenta) normalized to the control (grey line, no IL2 in the media). Data is the mean +/- SE; N and p-values shown in source data. Scale bar is 5 μm**. (D-F)** Samples were also prepared for TIRFM imaging. Cells were fixed after 15 min of SLB exposure in the presence or absence of 50 U/ml of IL2 in the media. Images on the left are representative examples. Dot plots on the right show the quantification of the mean intensity values (MGV) at the synapse for the indicated proteins (TCR, green; ICAM1, cyan; CD80, yellow; and CD58, magenta) normalized to the control (no IL2). Data is the mean +/- SE, Mann-Whitney test. Scale bar is 5 μm**. (G)** Naive, memory or blasted RA14 $CD8^+$ T cells were exposed to antigenic pMHC SLBs to form ISs. When indicated, 50 U/ml of IL2 were simultaneously added. Samples were fixed after 10-15 min of SLB and IL2 exposure and imaged with IRM. The image was used to segment the cell contact with the SLB (IS area) and the area of the contact was measured. Data is the mean +/- SE, Mann-Whitney test. Scale bar is 10 μm**.**

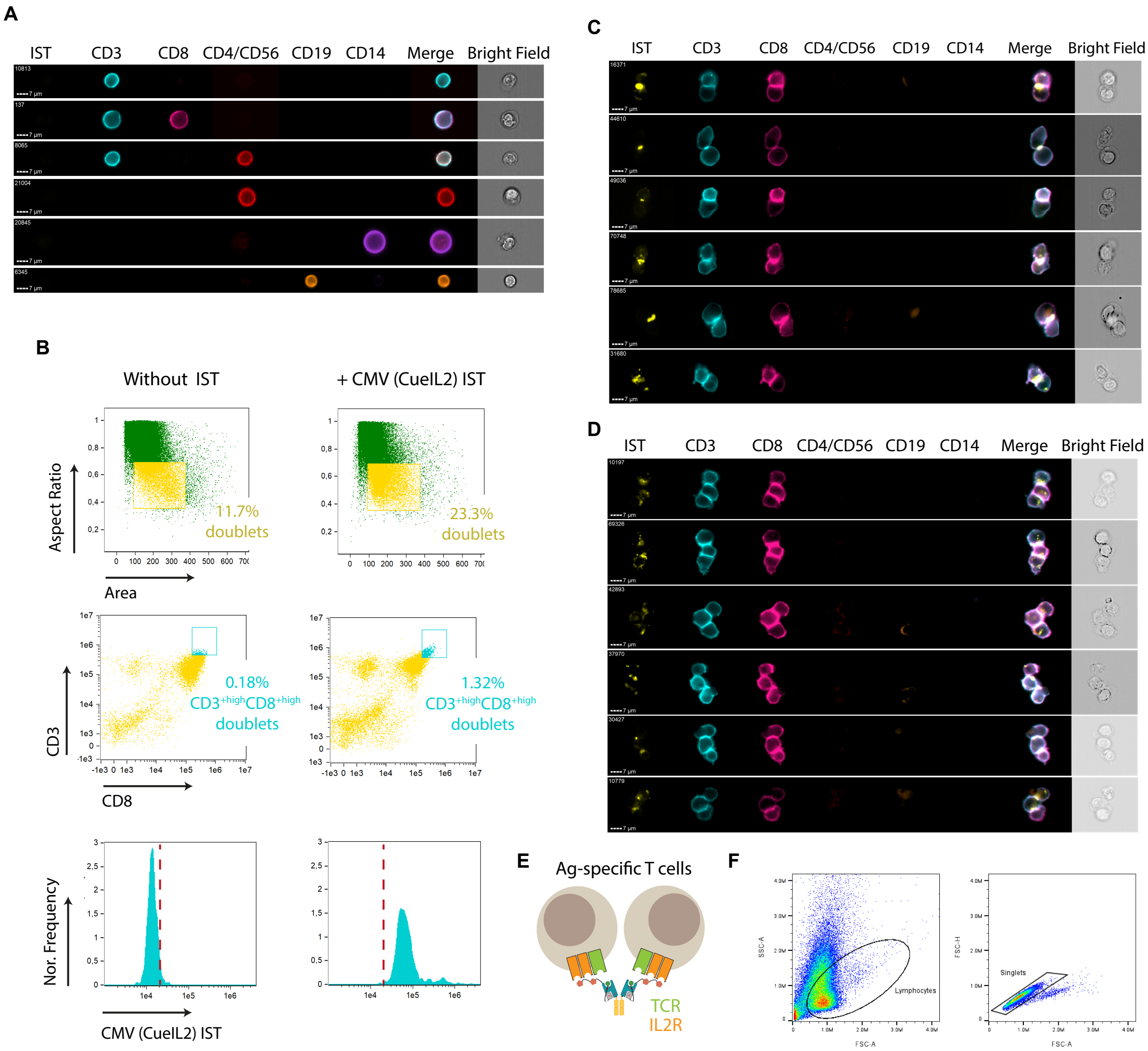

Supplementary figure 5: *Imaging Flow Cytometry* **(A)** Representative images of cell singlets stained with the different surface markers used for ImageStream analysis. Scale bar is 7 µm. **(B)** Gating strategy for Figure 3B. Doublets were further gated to identify possible $CD8^+$ T-T cell conjugates (Dot plots, high values for CD3 and CD8 markers, cyan population). Percentages of $CD3^{+high}CD8^{+high}$ doublets were quantified from the total focused events. Histograms show the intensity for CMV IL2 IST for the cyan population (possible $CD8^+$ T-T cell conjugates). **(C)** Representative examples of $CD8^+$ T-T cell conjugates identified. Note the accumulation of IST at the contact between the two T cells. Data is representative from three independent donors with similar results. Scale bar is 7 µm. **(D)** Representative examples of $CD8^+$ T cell multifocal clusters. Note the accumulation of IST at the contact between any two T cells. Scale bar is 7 µm**.** Data is representative from three independent donors with similar results. **(E)** Putative model for IST-mediated homotypic synapses between two antigen-specific $CD8^+$ T cells. **(F)** Gating strategy for figure 3M.

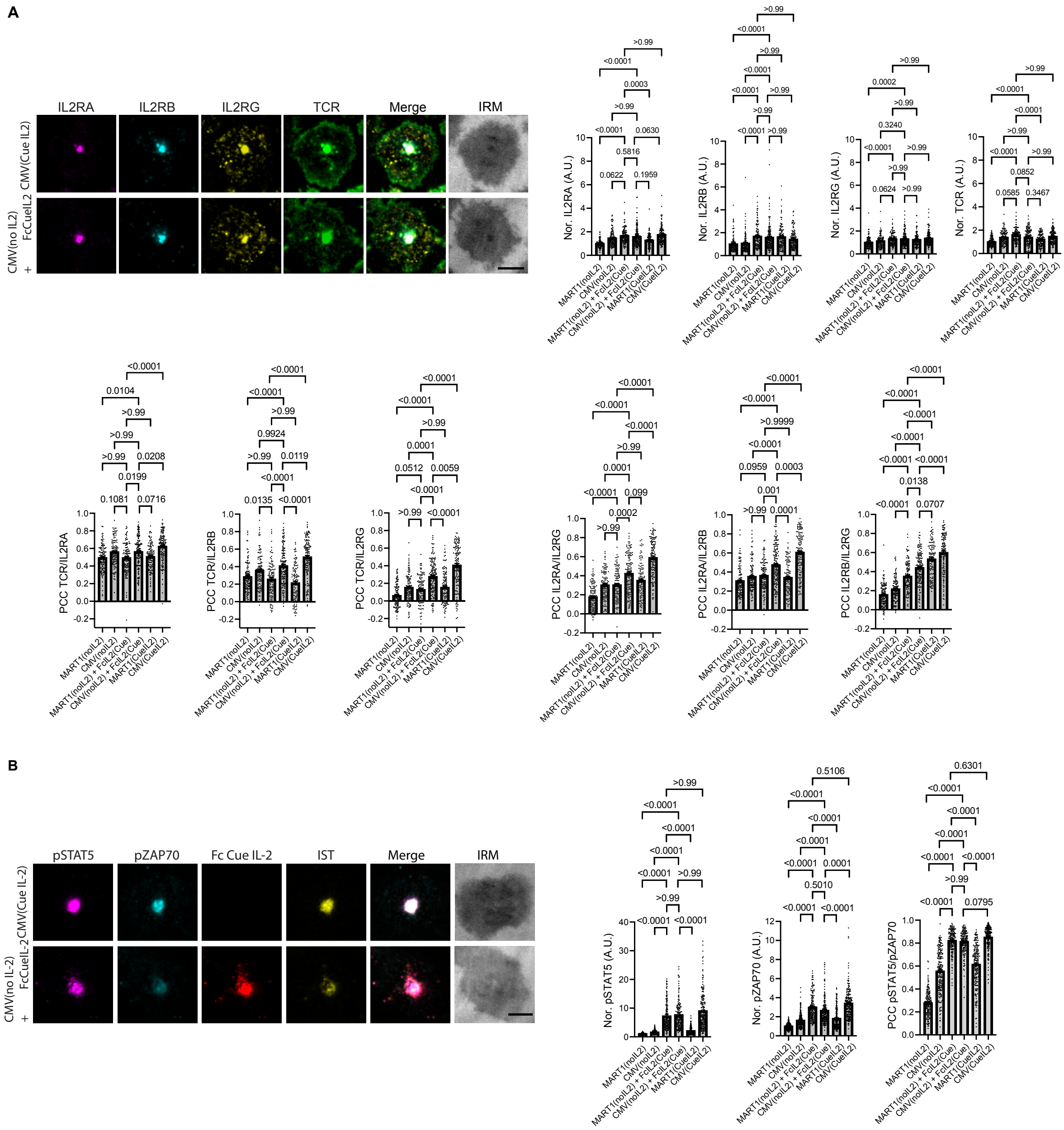

Supplementary figure 6: *Molecular link between IL2 and pMHC signals promotes IL2* receptor *assembly and TCR proximity and boosts signaling of both receptors.* **(A)** Representative TIRF images of RA14 CD8+ Blast T cell forming a IS with a SLB containing cognate ISTs (CMV), ICAM-1, CD80 and CD58. The CueIL2 molecule was part of the IST structure (CMV CueIL2) or presented as a separate Fc molecule (FcCueIL2) together with an IST without IL2 (CMV noIL2). Cells were fixed at 15 min after SLB exposure and immunolabelled against the three subunits of the IL2 receptor (IL2RA, magenta; IL2RB, cyan; IL2RG yellow) and the TCR (green). Plots show quantification of the mean grey value for the IL2 receptor subunits and the TCR at the IS (data is normalized to the control (MART1 noIL2)) and the Pearson's correlation coefficient (PCC) between all pairs of IL2 receptor subunits and the TCR. Data is the mean +/- SE, n= 114 (MART1(noIL2)), 110 (CMV(noIL2)), 112 (MART1(noIL2)+FcCueIL2)), 142 (CMV(noIL2)+FcCueIL2)), 111 (MART1(CueIL2)) and 127 (CMV(CueIL2)) cells, from 3 independent donors; Kruskal-Wallis test was used. **(B)** Representative TIRF images of RA14 CD8+ Blast T cells forming an IS with a SLB containing cognate ISTs AF488 (CMV, in yellow), ICAM-1, CD80 and CD58 and, when indicated, the FcCueIL2 AF568 (red). Cells were fixed at 15 min after SLB exposure and stained

against pSTAT5 (magenta) and pZAP70 (cyan). Plots show quantification of the mean grey value for pSTAT5 and pZAP70 at the IS (data is normalized to the control (MART1 noIL2)) and Pearson's correlation coefficient (PCC) between pSTAT5 and pZAP70. Data is the mean +/- SE, n= 170 (MART1(noIL2)), 208 (CMV(noIL2)), 167 (MART1(noIL2)+FcCueIL2)), 166 (CMV(noIL2)+FcCueIL2)), 184 (MART1(CueIL2)) and 201 (CMV(CueIL2)) cells, from 3 independent donors; Kruskal-Wallis test was used. Only pairwise comparisons with a FcCueIL2 condition are shown. Dataset is an extension of Fig. 5. Scale bar is 5 µm.

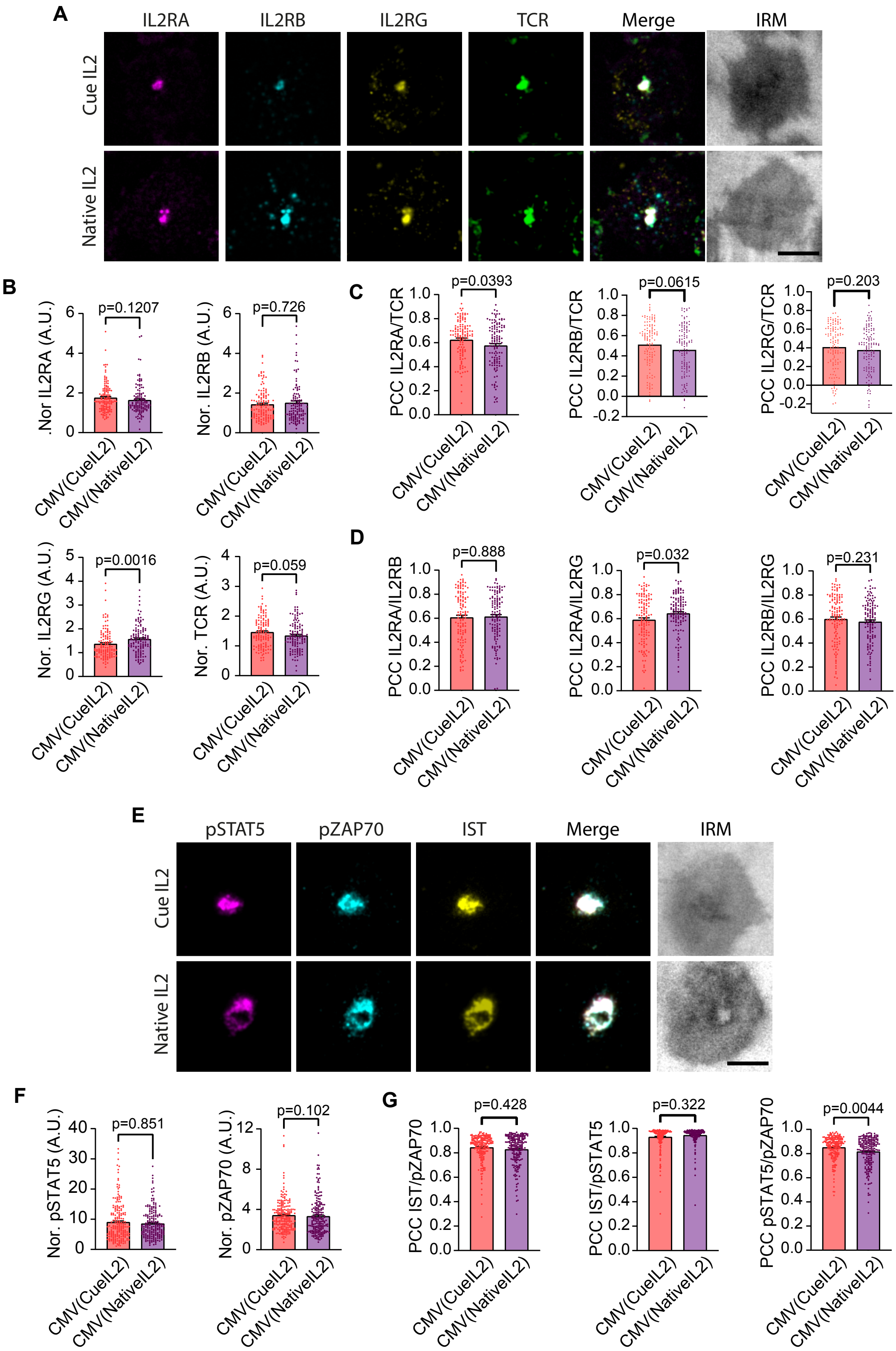

Supplementary figure 7: *IL2/IL2RA binding is not required for IL2 receptor assembly and activity at the IS.* **(A)** Representative TIRF images of RA14 CD8⁺ Blast T cells forming an IS with a SLB containing cognate ISTs (CMV) with ICAM-1, CD80 and CD58. The IST molecule contained either CueIL2 or NativeIL2. Cells were fixed at 15 min after SLB exposure and stained against the three subunits of the

IL2 receptor (IL2RA, magenta; IL2RB, cyan; IL2RG yellow) and the TCR (green). **(B)** Quantification of the mean grey value for the IL2 receptor subunits and the TCR at the IS normalized to the control (MART1 noIL2, data shown in Fig5). Data is the mean +/- SE, n=127 cells for CMV(CueIL2) and n= 124 for CMV(NativeIL2), from 3 independent donors, Mann-Whitney test. **(C)** Pearson's correlation coefficient (PCC) between the IL2 receptor subunits and the TCR and **(D)** between each pair of IL2 receptor subunits. Data is the mean +/- SE, n=125 cells from 3 independent donors; *pv<0.05; Mann-Whitney test. **(E)** Representative TIRF images of RA14 $CD8^+$ Blast T cells forming a IS with a SLB containing cognate ISTs (CMV, yellow). The IST molecule contained either CueIL2 or NativeIL2. Cells were fixed at 15 min after SLB exposure and stained against pSTAT5 (magenta) and pZAP70 (cyan). **(F)** Quantification of the mean grey value for pSTAT5 and pZAP70 at the IS normalized to the negative control (MART1 noIL2, data shown in Fig5). Data is the mean +/- SE, n=201 cells for CMV(CueIL2) and n= 205 for CMV(NativeIL2), from 3 independent donors, Mann-Whitney test. **(G)** Pearson's correlation coefficient (PCC) between the indicated IL2 receptor subunits and the TCR. Data is the mean +/- SE, n=201 cells for CMV(CueIL2) and n= 205 for CMV(NativeIL2), from 3 independent donors, Mann-Whitney test. Scale bar is 5 µm**.**
