# Supplementary material for: Spatiotemporal co-regulation of IL2 and TCR receptors reveals early antigen-driven synaptic synergy in human CD8+ T cells
Source: Nat Commun. 2026 Oct 7;17:11431. doi: 10.1038/s41467-026-77110-1 (PMC13737045; doi:10.1038/s41467-026-77110-1)

nature portfolio

Peer Review File

Spatiotemporal co-regulation of IL2 and TCR receptors reveals early antigen-driven synaptic synergy in CD8+ T cells

Corresponding Author: Dr Jesusa Capera

This file contains all reviewer reports in order by version, followed by all author rebuttals in order by version.

Version 0:

Reviewer comments:

Reviewer #1

(Remarks to the Author)
This is an interesting study that explores the cell surface localization of all IL-2R subunits for CD8+ T cells subsets in response to IL-2 and TCR stimulation. Notably they identify recruitment and removal of IL-2R subunit to the immunological synapse. Moreover, the high affinity IL-2R is co-localized with the TCR where they act to amplify each other's signals. This is an important finding as it provides a new context on how these two important signaling modules act in concert when T cells become activated in response to antigen. Although IL-2 has been previously detected in extracellular vesicles, they now find that some extracellular vesicles from recently activated CD8+ T cells contain IL-2 and IL-2R subunits and show that these are capable of inducing an IL-2R signaling in CD8 T cells. Overall, the study is well done, and the findings are clear. There are only several minor points to clarify.

1) The studies related to Figure 1 would be easier to follow if a diagram was included to indicate the scheme of these experiments. It is not so clear from the text and methods whether resting or memory cells are directly transfected without activation. The time when the transfected cells are analyzed and how they are identified after stimulation with the SLBs should be noted. Some of this is in the legend, but it would help with the clarity of the presentation.

2) What is the transfection frequency? How are the antigen-specific cells identified? This should be explicitly stated in the text.

3) It seems that the findings on the EV are a distraction from the main point of the manuscript. The functional experiments (Fig. 2) with the EV that contain IL-2 and all IL-2R subunits could simply be explained by the presence of IL-2. Fig. 2D, which lacks statistical analysis, provides no clear data that IL-2R or STAT5 in the same vesicle as IL-2 contribute to activation of IL-2R signaling in CD8+ T cells. Any conclusions in the results and the discussion should acknowledge this point.

4) What was the rationale of doing most experiments with an attenuated IL-2 CMV(CueIL2)? This should be discussed when this was first described near line 218.

5) Signaling is misspelled in line 374.

Reviewer #2

(Remarks to the Author)
Capera et al describe observations pertaining to the IL2R units localization and potential interactions with TCR complexes in CD8+ T cells. The authors find that the IL2 receptor subunits are differentially organized on CD8 T cell membranes and across CD8 subsets/activation states. They observe that the three receptor subunits are separately organized in EVs that are released from T cells. Interestingly, the EVs can induce pSTAT5 signaling greater than exogenous IL-2 when provided to naïve CD8s, likely due to a combination of IL2 receptor and signaling components that are found in the released EVs from activated CD8s. The authors demonstrate through impressive TIRF imaging that IL2 receptor and TCR signaling (pSTAT5 and Zap70 respectively) are spatiotemporally paired, likely inducing crosstalk that would be in line with findings of previous reports.

The authors then apply these observations toward providing a potential mechanism for why ISTs, a new biologic therapeutic platform, in trials may be superior therapeutic options to potently induce T cell responses. They propose that the combined cis-acting CueIL2 IST design exploits and potentiates the crosstalk and colocalization of TCR with IL2 receptor signaling to provide antigen specific augmentation of T cell activation signals. Of note, they find consistently that irrelevant peptide in ISTs with CueIL2 does lead to noticeable Zap70 and signaling.

Overall, the authors have made an impressive effort to dissect this observed correlation between TCR and IL2 receptor organization in CD8 T cells, providing additional support for the translation and promise of IST therapeutics. I greatly appreciated the extensive discussion, particularly when referencing past studies showing cytokine independent JAK/STAT signaling via interactions with TCRs. The authors findings add nuance to our understanding of the earliest signaling events in how additional signals may influence antigen recognition and support the further development of ISTs. This manuscript seems suitable for publications with modest changes.

Critiques and Suggestions:

1. Title and Abstract:
The paper relies largely on observations of signaling pathway initiation (i.e. pSTAT5 and Zap70 being colocalized). A title change would be worthwhile to emphasize that these investigators have only studied the very early signaling measure. Alternatively, can the authors do a simple proliferation experiment to demonstrate that the colocalization (or absence of colocalization and strength of TIRF signal) does indeed influence functional T cell outcomes. The authors could provide basic some functional readouts that show how the presence or absence of colocalization (and the degree of signal found via TIRF) influences the downstream T cell activation program (cytokine production, proliferation, etc.). This is perhaps especially important in the context of the IST CueIL2 MART1 experiments where there are marked levels of ZAP70 being observed even in the absence of TCR engagement. The Zap70 signal seen by IL-2 signaling without TCR engagement is likely distinct from the CMV CUEIL2 signal and might provide insight into off-target events that may be associated with these therapeutics. Given that this paper is a bit of an advertisement for these drugs, it would seem worthwhile to address this at very least in text.

2. The apparent CD25 on "naïve" T cells:
In Figure 1D-end, it is not clear the CD25 status of the T cells. Were these antigen-experienced (or importantly CD25 positive) by the time they were treated? One might believe that naïve means to antigens before hitting the SLB but then one wouldn't expect to have any IL2RA present. Flow plots of CD25 levels in each of the populations in D are required to understand this for each population, especially in the context of Figure 1A-C which would seem to say (and historical evidence, cited by these authors would support) that naïve cells don't express CD25 (unless they are Treg). The naming of populations may need to be adjusted or accommodated between 1-C and the rest of the work.

Similarly line 214: The synapse contact area was also larger in the presence of IL2 for naive and blast CD8+ T cells (Fig. S2G). Given that the vast majority of published flow data shows no shift for CD25 antibodies on truly naïve cells, this result begs the same question as above. This needs either clean explanation (are the authors saying that CD25 IS present in naïve cells at the protein level?) or perhaps rethinking.

3. The use of TIRF as a quantitative measure of recruitment:
Figure 1C which purports to demonstrate reductions in CD25 intensity is not controlled for the difference in illumination conditions and depth of TIRF field between experiments. This is clearly highly variable as these practitioners likely know and so either that should be normalized in some way (difficult to do but could involve a standard bead that drops to the TIRF field) or eliminated or perhaps simply done by flow. The result seems to follow some kind of expectations but that does not mean it is solid data or should be treated as such, without being properly controlled.

Figure 3HJL suffer from the same issue as 1C, regarding capturing AU from different physical slides/setups unless some normalization has been applied. The differences are modest in any case. Is this conclusion necessary for the results of the work? I don't think so, so the easiest out is to either supplementalize it and note the caveats raised here OR simply omit it. I know it was some work to make these measurements but we also know as Mike will that they aren't very clean numbers between coverslips, technically (unless some normalization method is being applied at acquisition?). Figure 3DEF should be fine as what is being plotted is the same field/cell over time (noting of course that bleaching could be one reason for the dropoff and represent some uncertainty in the Y-axis, although bleaching is simply not likely to occur in the single steps that are seen here so the conclusions as stated seem robust).

And this is critically important for Figure 6 in which Cue is meant to show higher recruitment, a key result that the company no doubts is interested in seeing (see notes too about functional meaning and item 1 re: title/abstract level conclusions). Here it looks like quite a few cells had to be viewed to get the stats up, which is probably consistent with this technical issue.

4. What is the nature of the IL2 in the SLBs?
Many cytokines (see e.g. Alton-Bonnet for gIFN) and chemokines immobilize to membranes. Is there IL2 already on the SLB at the time of this biology? The setup for the SLB in Figure 1 needs a bit more detail to understand this. Text line ~127 is quite silent on this issue as is the Figure legend. Given the topic of the paper, providing this information here is an important omission (they do so later but don't address whether cytokine might be presented on the SLB. The authors have some recognition as per Figure S1A but that text surrounding that is not supported by the figures, which actually shows no difference between IL2 and no IL2: line 142: "The centralized (focal and annular) phenotypes of IL2 receptor subunits were

more frequent when the IS was established in the presence of IL2 (Fig. S1A)."

5. Receptors being dissociated versus never associated and TIRF depth issues:
Figure 1I is interesting insofar as different patterns are seen for different subunits. Can the authors convince us/themselves that the gamma (being apparently more inside) isn't just a consequence of the fluorophore being used (either being brighter overall or being of a wavelength that results in the TIRF field being deeper?). The discussion provides a precedence for this happening and for differential localizations of various subunits but still would be nice to have the technical explanation invalidated.

Figure 3B/C convincingly shows the main biological point; that pSTAT5 co-localizes with ZAP70 in microvillar contacts in early times, though segregates later. This is enhanced by addition of IL-2 but again does not require it. The text around this appears consistent with the data.

6. Small item of interpretation:
Line 206 the IS activated pSTAT5: since we don't know that STAT5 doesn't activate elsewhere (or is pre-activated) and trafficked to the synapse, phrasing might better be: pSTAT5was localized in the active IS

Just as a note:
The second figure relating to the release of EVs feels less central to the primary focus on IL2R cross talk with TCR that the other figures and story is framed around. While it is definitely interesting to consider the receptor subunit organization in these EVs, these findings may be better suited in extended/supplementary figures so the central manuscript focuses on IL2 receptor localization on the membrane and synapse to set up the justification for exploring how ISTs impact T cells (and why they may be superior to past IL2 trials).

And for discussion, perhaps:
Have the authors considered how other cytokines may also feed into this type of synergistic pathway with TCRs? The discussion brings up the trans presentation of IL-15 by APCs but given the sharing of IL2RG across various cytokines that shape T cell differentiation trajectories, there may be subtle differences induced by other cytokines that may be interesting to explore in the discussion. However, adding extra experiments for this would be unnecessary for publication and out of the scope of this paper.

Reviewer #3

(Remarks to the Author)
I co-reviewed this manuscript with one of the reviewers who provided the listed reports. This is part of the Nature Communications initiative to facilitate training in peer review and to provide appropriate recognition for Early Career Researchers who co-review manuscripts.

Version 1:

Reviewer comments:

Reviewer #1

(Remarks to the Author)
No new comments

Reviewer #4

(Remarks to the Author)
The authors have carefully addressed each of the remarks raised by Reviewer 2.

I just want to point to a couple of issues with the new data added to the revised manuscript:

Related to point 1 raised by Reviewer 2:

New Fig3M: the numbers at the bottom of the figure seem to refer to the number of replicates. This is not needed since the individual replicates can be seen from the dots on the figure (actually 6 and not 5 replicates for the bead SLB condition). What should however be indicated in the legend is what those replicates are (technical replicates? Individual donors? other?).

New Fig3M: the grey labels of the representative histograms are a bit dim. Maybe black labels would ease the reading.

Related to point 2 raised by Reviewer 2:

New FigS1B: blasts start expressing CD45RO while maintaining CD45RA. Is that expected? CD45RA signal seems to be aberrantly high (actually still very positive signal in memory cells). Can the authors review their flow cytometry analysis and ajust it if applicable?

Open Access This Peer Review File is licensed under a Creative Commons Attribution 4.0 International License, which permits use, sharing, adaptation, distribution and reproduction in any medium or format, as long as you give appropriate credit to the original author(s) and the source, provide a link to the Creative Commons license, and indicate if changes were made.
In cases where reviewers are anonymous, credit should be given to 'Anonymous Referee' and the source.
The images or other third party material in this Peer Review File are included in the article's Creative Commons license, unless indicated otherwise in a credit line to the material. If material is not included in the article's Creative Commons license and your intended use is not permitted by statutory regulation or exceeds the permitted use, you will need to obtain permission directly from the copyright holder.
To view a copy of this license, visit https://creativecommons.org/licenses/by/4.0/

RESPONSE TO REVIEWERS' COMMENTS

Reviewer #1 (Remarks to the Author):

This is an interesting study that explores the cell surface localization of all IL-2R subunits for CD8+ T cells subsets in response to IL-2 and TCR stimulation. Notably they identify recruitment and removal of IL-2R subunit to the immunological synapse. Moreover, the high affinity IL-2R is co-localized with the TCR where they act to amplify each other's signals. This is an important finding as it provides a new context on how these two important signaling modules act in concert when T cells become activated in response to antigen. Although IL-2 has been previously detected in extracellular vesicles, they now find that some extracellular vesicles from recently activated CD8+ T cells contain IL-2 and IL-2R subunits and show that these are capable of inducing an IL-2R signaling in CD8 T cells. Overall, the study is well done, and the findings are clear. There are only several minor points to clarify.

1) The studies related to Figure 1 would be easier to follow if a diagram was included to indicate the scheme of these experiments. It is not so clear from the text and methods whether resting or memory cells are directly transfected without activation. The time when the transfected cells are analyzed and how they are identified after stimulation with the SLBs should be noted. Some of this is in the legend, but it would help with the clarity of the presentation.

We thank the reviewer for this helpful suggestion, which has improved the clarity of our manuscript. As recommended, we have now included a schematic diagram illustrating the experimental workflow (new Fig. S1A, see below). In addition, we have revised the Materials and Methods section to provide further detail (lines 489, 495 and 514).

Briefly, total $CD8^+$ T cells were purified from leukoreduction system (LRS) chambers and further separated into naive$CD8^+$ T cells ($CD8^+CD45RA^+CCR7^+CD45RO^-CD57^-CD56^-$) and memory $CD8^+$ T cells ($CD8^+CD45RA^-CD45RO^+$) using immunomagnetic negative selection kits (STEMCELL Technologies™). These subsets were then electroporated with RA14 TCR mRNAs and cultured without any pre-activation step (no cytokines or Dynabeads were added). Cells were used for experiments 18–24 h post-electroporation.

For activated $CD8^+$ T cells (Blast), total $CD8^+$ cells were stimulated with anti-CD3/CD28 Dynabeads for 3 days and maintained with 50 U/ml IL-2 until day 7, when they were electroporated with RA14 mRNAs. These cells were likewise used 18–24 h post-electroporation. Successful transfection was confirmed by cognate tetramer staining (new Fig. S1D).

To minimize imaging bias during TIRF acquisition, the field of view was selected and focused based solely on the interference reflection microscopy (IRM) channel, which enabled unbiased identification of cell–bilayer contacts prior to examining fluorescent channels.

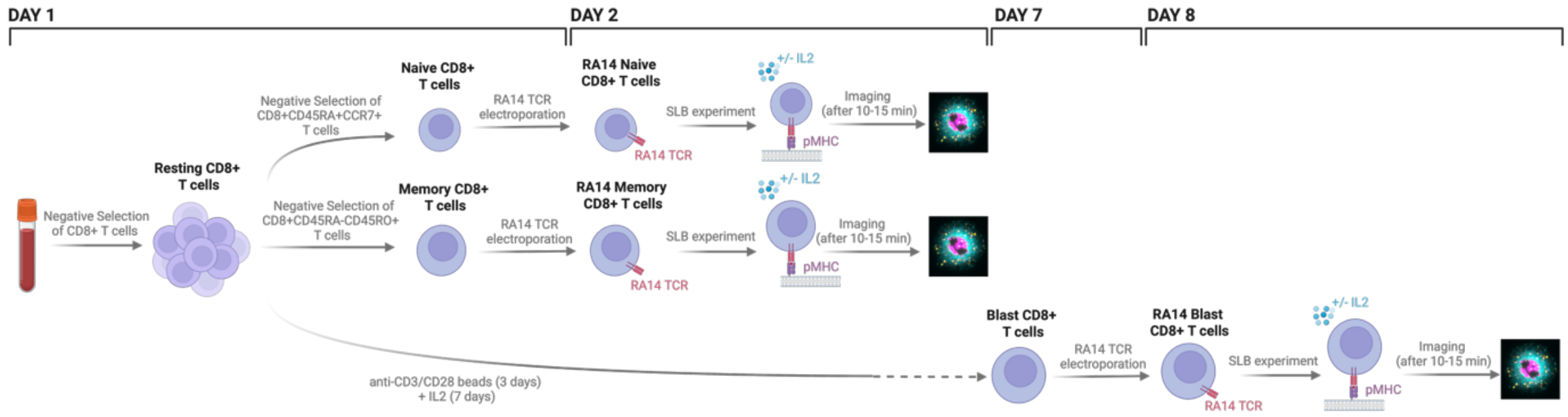

2) What is the transfection frequency? How are the antigen-specific cells identified? This should be explicitly stated in the text.

Thank you for raising this important point. Antigen-specific RA14⁺ T cells were identified by cognate tetramer staining (APC–HLA-A*02:01 CMV pp65 tetramer). As shown in the plot below, the transfection efficiency was consistently high across multiple blood donors, averaging 84% ± 1.21 (SE). The frequency of RA14⁺ cells and the surface intensity of RA14 TCR were comparable to those observed in the positive control SKW3 cell line stably expressing RA14 TCR. Non-electroporated CD8⁺ T cells from the same donors were used as a negative control. These data have been incorporated into the Results section (new Fig. S1E), and the Materials and Methods have been updated to describe the tetramer staining protocol in detail.

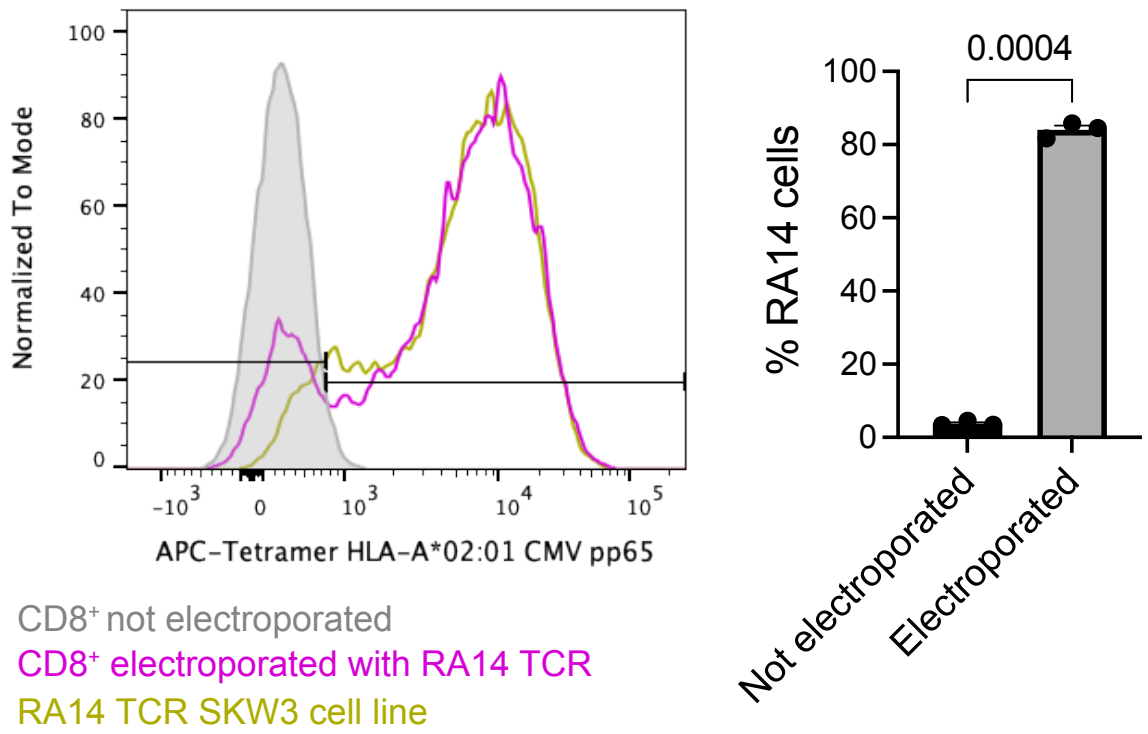

3) It seems that the findings on the EV are a distraction from the main point of the manuscript. The functional experiments (Fig. 2) with the EV that contain IL-2 and all IL-2R subunits could simply be explained by the presence of IL-2. Fig. 2D, which lacks statistical analysis, provides no clear data that IL-2R or STAT5 in the same vesicle as IL-2 contribute to activation of IL-2R signaling in CD8+ T cells. Any conclusions in the results and the discussion should acknowledge this point.

We thank the reviewer for this constructive comment. We agree that the observed STAT5 activation can be largely attributed to the presence of EV-associated IL2. In line with this, we have revised the Results section (lines 188-198) to clarify this interpretation and to emphasize that further studies will be required to precisely identify which EV cargo components are responsible for the STAT5 activation observed in naive CD8⁺ T cells.

In addition, following the reviewer's comment and to maintain a clear focus on the main findings of the manuscript, we have moved the previous Figure 2 to the supplementary

materials (new Fig. S3). This adjustment allows the EV-related data to remain accessible while ensuring that the central narrative of the study remains focused.

4) What was the rationale of doing most experiments with an attenuated IL-2 CMV(CueIL2)? This should be discussed when this was first described near line 218.

Thank you for your question. The attenuated IL2 variant was used to enhance the Immuno-STAT selectivity for target T cells while minimizing potential toxicity associated with systemic IL2-driven activation of other IL2R-expressing cells, as previously described in *Quayle et al., 2020 (Clin Cancer Res)*. We have now included this clarification in the Results section (line 235) and discussion (lines 441-448).

5) Signaling is misspelled in line 374.

Thank you; the misspelling has been corrected.

Reviewer #2 (Remarks to the Author):

Capera et al describe observations pertaining to the IL2R units localization and potential interactions with TCR complexes in CD8+ T cells. The authors find that the IL2 receptor subunits are differentially organized on CD8 T cell membranes and across CD8 subsets/activation states. They observe that the three receptor subunits are separately organized in EVs that are released from T cells. Interestingly, the EVs can induce pSTAT5 signaling greater than exogenous IL-2 when provided to naiveCD8s, likely due to a combination of IL2 receptor and signaling components that are found in the released EVs from activated CD8s. The authors demonstrate through impressive TIRF imaging that IL2 receptor and TCR signaling (pSTAT5 and Zap70 respectively) are spatiotemporally paired, likely inducing crosstalk that would be in line with findings of previous reports.

The authors then apply these observations toward providing a potential mechanism for why ISTs, a new biologic therapeutic platform, in trials may be superior therapeutic options to potently induce T cell responses. They propose that the combined cis-acting CueIL2 IST design exploits and potentiates the crosstalk and colocalization of TCR with IL2 receptor signaling to provide antigen specific augmentation of T cell activation signals. Of note, they find consistently that irrelevant peptide in ISTs with CueIL2 does lead to noticeable Zap70 and signaling.

Overall, the authors have made an impressive effort to dissect this observed correlation between TCR and IL2 receptor organization in CD8 T cells, providing additional support for the translation and promise of IST therapeutics. I greatly appreciated the extensive discussion, particularly when referencing past studies showing cytokine independent JAK/STAT signaling via interactions with TCRs. The authors findings add nuance to our understanding of the earliest signaling events in how additional signals may influence antigen recognition and support the further development of ISTs. This manuscript seems suitable for publications with modest changes.

Critiques and Suggestions:

1. Title and Abstract:
The paper relies largely on observations of signaling pathway initiation (i.e. pSTAT5 and Zap70 being colocalized). A title change would be worthwhile to emphasize that these investigators have only studied the very early signaling measure. Alternatively, can the authors do a simple proliferation experiment to demonstrate that the colocalization (or absence of colocalization and strength of TIRF signal) does indeed influence functional T cell outcomes. The authors could provide basic some functional readouts that show how the presence or absence of colocalization (and the degree of signal found via TIRF) influences the downstream T cell activation program (cytokine production, proliferation, etc.). This is perhaps especially important in the context of the IST CueIL2 MART1 experiments where there are marked levels of ZAP70 being observed even in the absence of TCR engagement.

The Zap70 signal seen by IL-2 signaling without TCR engagement is likely distinct from the CMV CUEIL2 signal and might provide insight into off-target events that may be associated with these therapeutics. Given that this paper is a bit of an advertisement for these drugs, it would seem worthwhile to address this at very least in text.

We thank the reviewer for this important comment and agree that directly linking early synaptic signaling events to downstream functional outcomes strengthens the study. To address this point, we performed CFSE-based proliferation assays using both a bead–supported lipid bilayer (bead–SLB) and a solid-phase system to present ISTs. These new data have been incorporated into the revised manuscript (Figure 3M and line 286).

In these assays, CMV(CueIL2) induced the strongest proliferative response. Presentation of cognate antigen without IL2 (CMV(noIL2)) or CueIL2 linked to an irrelevant antigen (MART1(CueIL2)) resulted in minimal proliferation, and MART1(noIL2) showed no detectable expansion. The magnitude of proliferation consistently mirrored the degree of pSTAT5 and pZAP70 colocalization observed at the immunological synapse, with CMV(CueIL2) exhibiting both the strongest spatial signaling synergy and the greatest antigen-specific expansion.

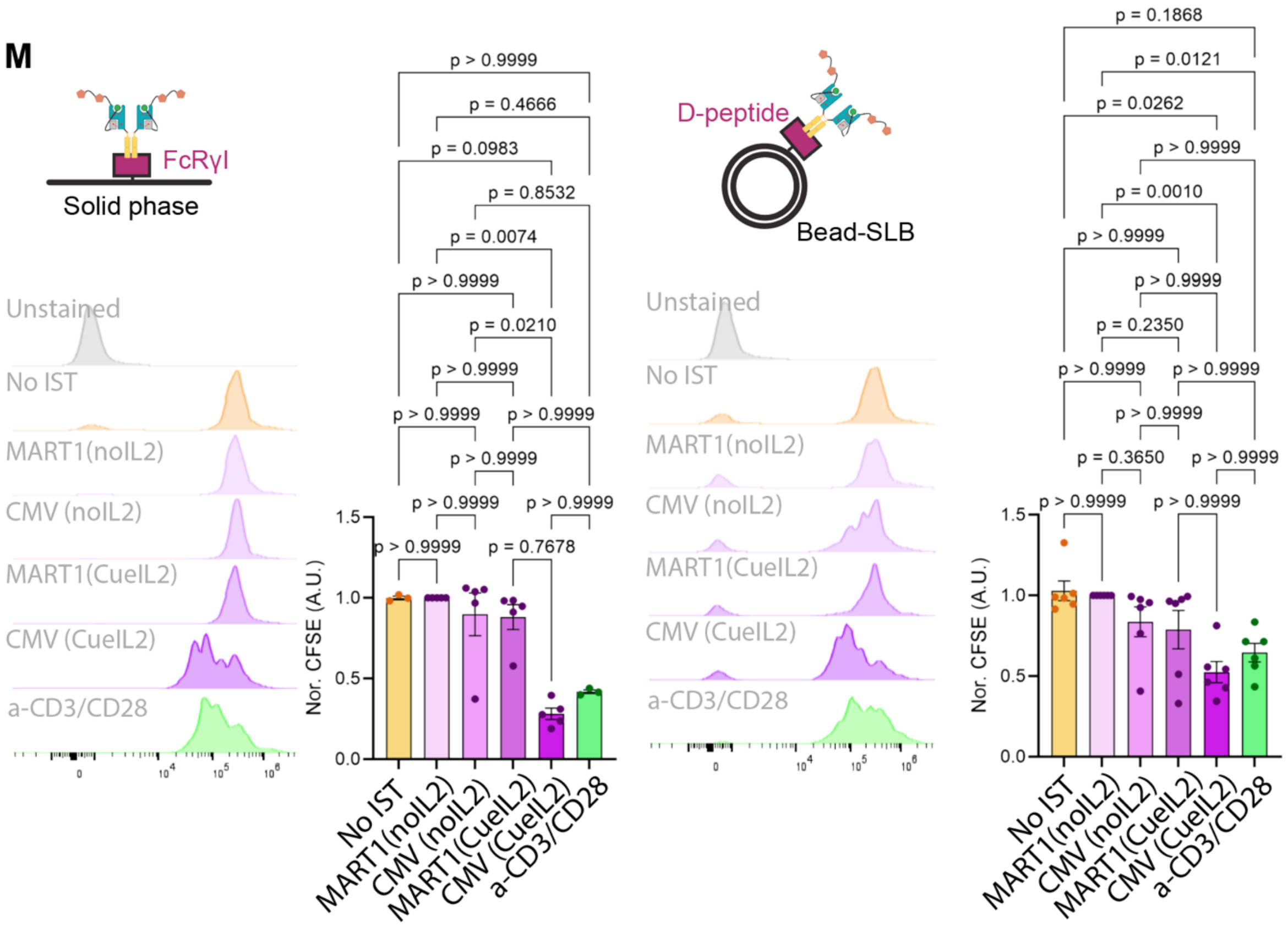

In addition, previously published *in vitro* and *in vivo* studies demonstrate selective activation, antigen-specific expansion, durable immunological memory, and anti-tumor activity of ISTs, together with a favorable safety profile and minimal off-target activity *(Quayle et al., Clin. Cancer Res., 2020).*

Therefore, the new functional data reinforce prior evidence that coordinated TCR–IL2R engagement by ISTs drives selective and biologically relevant T cell responses, and that the early spatial signaling patterns observed by TIRF microscopy translate into controlled, antigen-specific functional outcomes rather than nonspecific activation.

2. The apparent CD25 on "naïve" T cells:
In Figure 1D-end, it is not clear the CD25 status of the T cells. Were these antigen-experienced (or importantly CD25 positive) by the time they were treated? One might believe that naivemeans to antigens before hitting the SLB but then one wouldn't expect to have any IL2RA present. Flow plots of CD25 levels in each of the populations in D are required to understand this for each population, especially in the context of Figure 1A-C which would seem to say (and historical evidence, cited by these authors would support) that naivecells don't express CD25 (unless they are Treg). The naming of populations may need to be adjusted or accommodated between 1-C and the rest of the work.

We thank the reviewer for raising this important point regarding the CD25 status of the T cell populations used in our experiments. We agree that clear characterization of IL2RA (CD25) expression is essential for proper interpretation of these data.

To clarify the nature of the $CD8^+$ T cell subsets, we have added a schematic of the experimental workflow (new Fig. S1A). Briefly, $CD8^+$ T cells were purified by negative selection to obtain two resting populations: $CD8^+CD45RA^+CCR7^+$ cells (referred to as naive$CD8^+$ T cells) and $CD8^+CD45RA^-CD45RO^+$ cells (referred to as memory $CD8^+$ T cells). These cells were used within 24–48 hours of isolation and were not exposed to antigen stimulation or in vitro activation prior to the experiments. In contrast, blast $CD8^+$ T cells were generated by stimulating pooled resting $CD8^+$ T cells with anti-CD3/CD28 Dynabeads in the presence of IL2 for 7 days.

We have included additional phenotypic validation of these populations. New Fig. S1B shows representative CD45RA/CD45RO expression profiles. In addition, as suggested by the reviewer, we now provide flow cytometric analysis of CD25 expression across all subsets (Fig. S1C). Consistent with established literature, both naiveand memory $CD8^+$ T cells exhibit minimal CD25 expression, whereas blast $CD8^+$ T cells show robust CD25 upregulation.

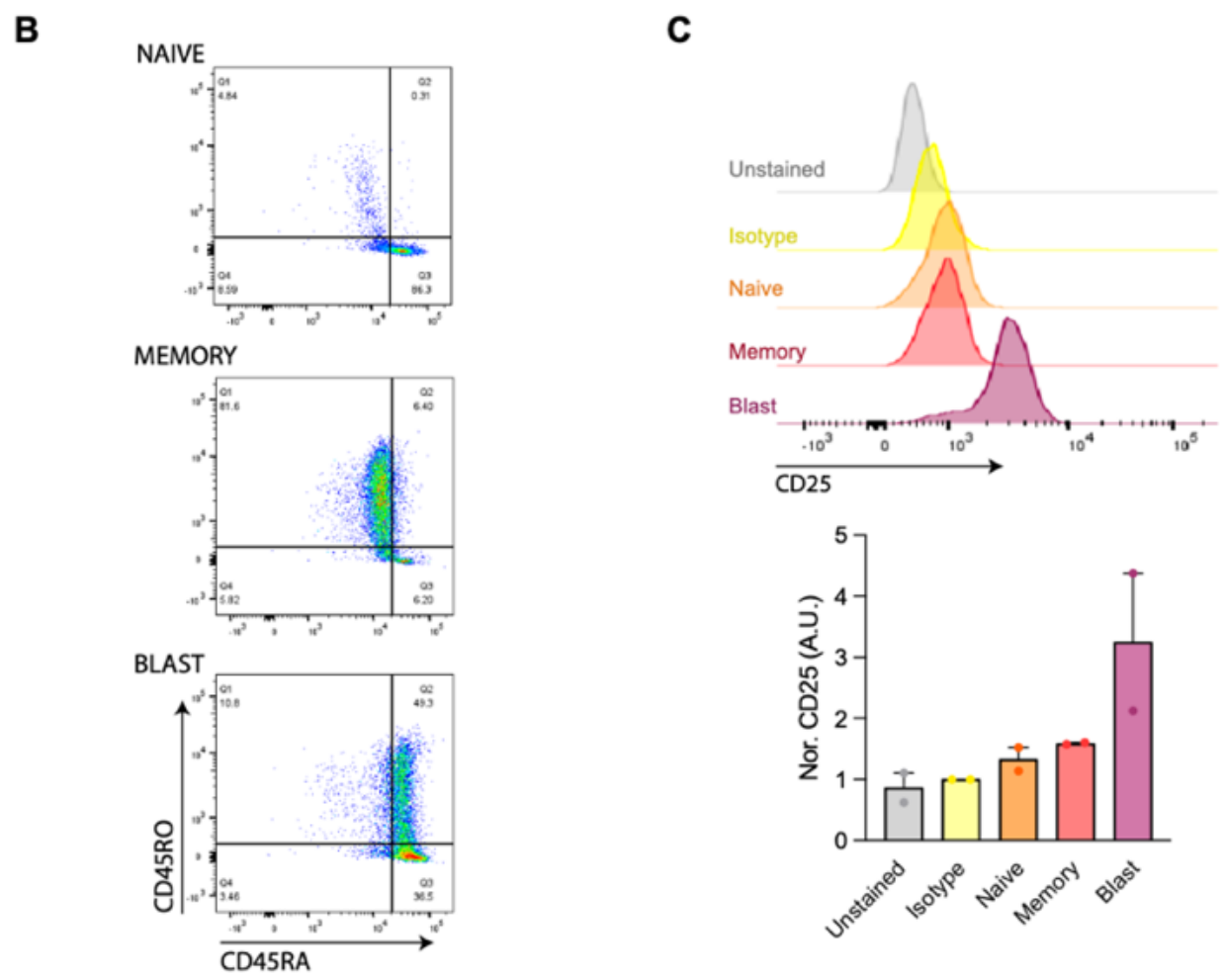

To further validate these observations in the TIRFM context, we repeated SLB experiments including isotype controls for the anti-CD25 antibody used (clone BC96; Fig. S1D, see below). In these experiments, CD25 signal in naïve CD8+ T cells was comparable to isotype control, while a clear and specific signal was detected in blast CD8+ T cells. These results are consistent with our scRNA-seq analysis (Fig. 1C) and with quantitative measurements of CD25 intensity at the immune synapse (Fig. S2B).

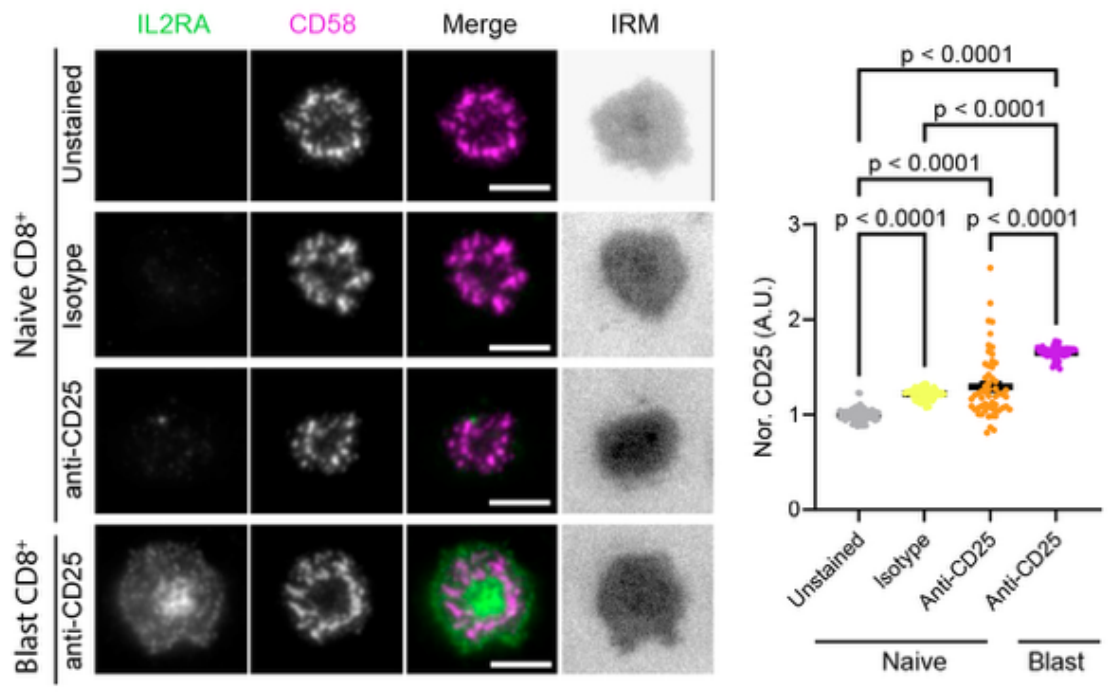

Based on these additional data, we conclude that the CD25 signal observed in naive and memory CD8+ T cells in the original Fig. 1D is very low and should be interpreted with caution within the limitations of antibody staining. To avoid overinterpretation, we have revised the manuscript accordingly and removed or reinterpret any analyses on IL2RA signal in these populations (including Fig. 1D, Fig.1H and Fig. S2A, S2C–D).

Finally, we would like to clarify the intent of the imaging shown in Fig. 1D–F. These panels were designed to illustrate the spatial organization of IL-2 receptor subunits at the immune synapse, rather than to compare absolute expression levels between cell types. To facilitate visualization of spatial patterns, image intensities were adjusted independently for each panel, which is a common practice for highlighting subcellular distribution. To improve clarity, we have now explicitly stated this in the figure legend. In addition, we have updated Fig. 1G so that all images are displayed using identical intensity scaling, allowing direct comparison of signal levels across cell populations.

Together, these additions and revisions clarify that resting naiveand memory CD8+ T cells in our system do not significantly express CD25, in agreement with prior literature, and ensure that the imaging data are interpreted appropriately.

Similarly line 214: The synapse contact area was also larger in the presence of IL2 for naive and blast CD8+ T cells (Fig. S2G). Given that the vast majority of published flow data shows no shift for CD25 antibodies on truly naivecells, this result begs the same question as above. This needs either clean explanation (are the authors saying that CD25 IS present in naivecells at the protein level?) or perhaps rethinking.

Naive CD8+ T cells are still capable of responding to IL2 through the intermediate-affinity IL2 receptor, composed of IL2RB (CD122) and the common γ chain (IL2RG/CD132), which

are expressed on these cells. As shown in Fig. 2A, naive$CD8^+$ T cells exhibit a detectable, although markedly weaker, pSTAT5 response to IL2 compared to memory or blast $CD8^+$ T cells. This observation is consistent with signaling through the intermediate-affinity IL2 receptor complex. We therefore interpret the modest increase in synapse contact area observed in the presence of IL2 (Fig. S4G) as a consequence of signaling mediated by IL2RB/G rather than by CD25.

Together, these data indicate that naive$CD8^+$ T cells retain the ability to respond to IL2 via the intermediate-affinity receptor, while maintaining minimal CD25 expression, consistent with prior reports.

3. The use of TIRF as a quantitative measure of recruitment:
Figure 1C which purports to demonstrate reductions in CD25 intensity is not controlled for the difference in illumination conditions and depth of TIRF field between experiments. This is clearly highly variable as these practitioners likely know and so either that should be normalized in some way (difficult to do but could involve a standard bead that drops to the TIRF field) or eliminated or perhaps simply done by flow. The result seems to follow some kind of expectations but that does not mean it is solid data or should be treated as such, without being properly controlled.
Figure 3HJL suffer from the same issue as 1C, regarding capturing AU from different physical slides/setups unless some normalization has been applied. The differences are modest in any case. Is this conclusion necessary for the results of the work? I don't think so, so the easiest out is to either supplementalize it and note the caveats raised here OR simply omit it. I know it was some work to make these measurements but we also know as Mike will that they aren't very clean numbers between coverslips, technically (unless some normalization method is being applied at acquisition?).

Figure 3DEF should be fine as what is being plotted is the same field/cell over time (noting of course that bleaching could be one reason for the dropoff and represent some uncertainty in the Y-axis, although bleaching is simply not likely to occur in the single steps that are seen here so the conclusions as stated seem robust).
And this is critically important for Figure 6 in which Cue is meant to show higher recruitment, a key result that the company no doubts is interested in seeing (see notes too about functional meaning and item 1 re: title/abstract level conclusions). Here it looks like quite a few cells had to be viewed to get the stats up, which is probably consistent with this technical issue.

For the concerns about the 2D uniformity of Total Internal Reflection Fluorescence Microscopy (TIRFM)

To address these concerns, we performed TIRF imaging across four fluorescence channels: 405 nm, 488 nm, 561 nm, and 647 nm. We prepared SLBs containing 1% of each fluorescent DOPE variant (Atto 390, Atto 488, Atto 565, and Atto 647) and 96% DOPC. These SLBs

were imaged using TIRFM under laser powers and camera exposure times comparable to those used in our experiments (Fig. 1a).

We quantified the relative mean fluorescence intensities (MFI) along both X and Y line profiles within 60 µm × 60 µm imaging regions. Across all channels, the intensity profiles exhibited bell-shaped distributions, with higher relative MFI at the center and lower values toward the edges (Fig. 1b). The standard deviations for all channels were below 0.2, indicating that the measured intensities can be approximated as the mean value with 20% variance (Fig. 1c). These data demonstrate that the 2D illumination uniformity of our TIRFM system is excellent, owing to optimal alignment of the TIRF illuminator, objective, camera, and other optical components. Therefore, our TIRFM setup enables the acquisition of imaging data with low variability, which is essential for accurate quantitative analysis

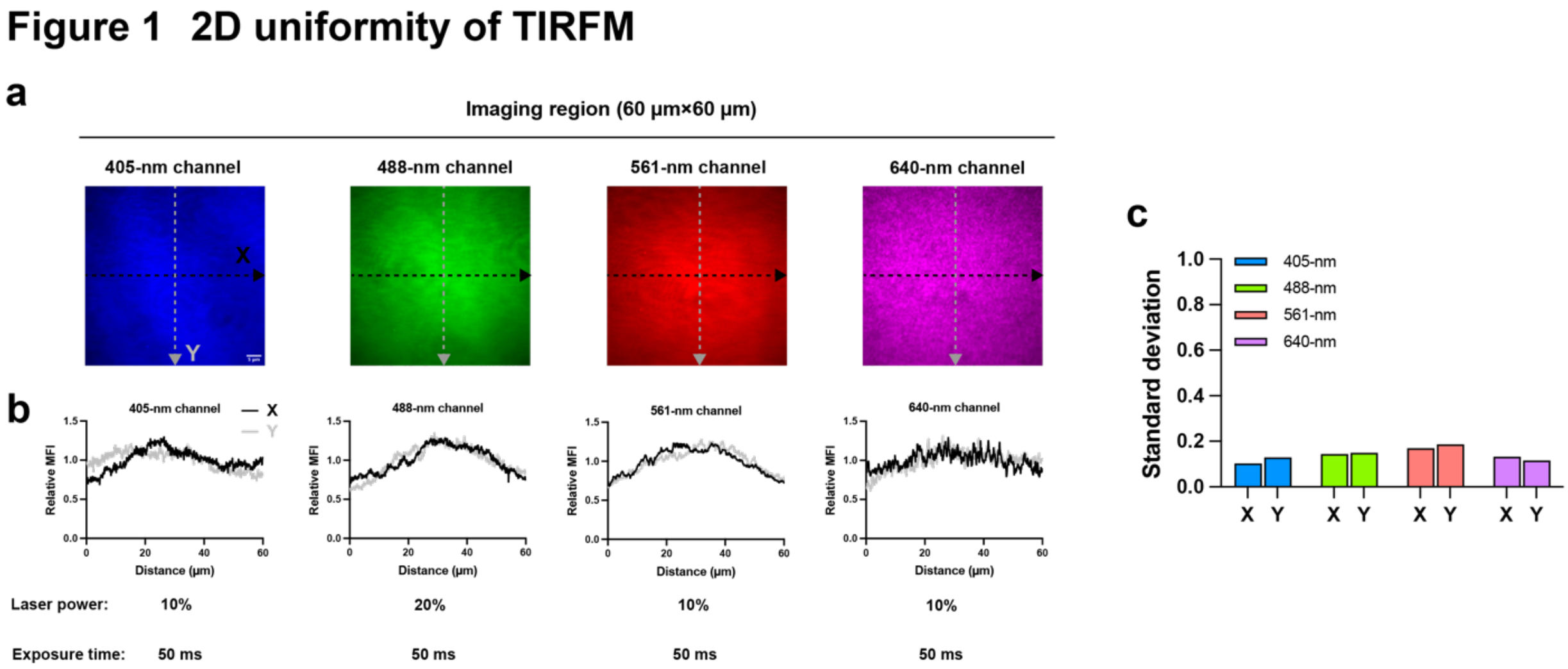

Fig. 1 | 2D uniformity of Total Internal Reflection Fluorescence Microscopy (TIRFM). a, Images of the supported lipid bilayers (SLBs) composed of 1% of each fluorescent DOPE and 99% DOPC, captured using fluorescence channels at 405 nm, 488 nm, 561 nm, and 647 nm. X and Y plot-profile positions are marked by dashed arrows. **b**, Mean fluorescence intensities (MFI) along the plot profiles obtained under laser powers with 50-ms exposure times; fluorescence intensities are relative to the average. **c**, Standard deviations of relative MFI in all channels.

For the concerns about the stability of TIRFM

To address these concerns, we took advantage of the capability of TIRFM to visualize single molecules. We diluted UCHT-1 Fab AF647 protein molecules and allowed them to adsorb onto the coverslip. With appropriate dilution, we were able to isolate and image individual UCHT-1 Fab AF647 molecules, and then quantify their mean fluorescence intensities (Fig. 2a). We retrieved UCHT-1 Fab AF647 proteins from the −80 °C freezer and designated these samples as “fresh”, we performed single-molecule quantification experiments using the “fresh” samples. We then stored the same protein at 4 °C for two months. We performed

single-molecule quantification experiments using “old” samples under identical experimental conditions. We did not detect significant differences in the mean intensities between single “old” and single “fresh” UCHT-1 Fab AF647 molecules. These findings indicate that both the protein samples and our TIRFM system remain stable over extended periods, allowing us to generate highly reproducible results (Fig. 2b). This experiment indicates stability of the fluorescently tagged antibody, and the imaging system, over time.

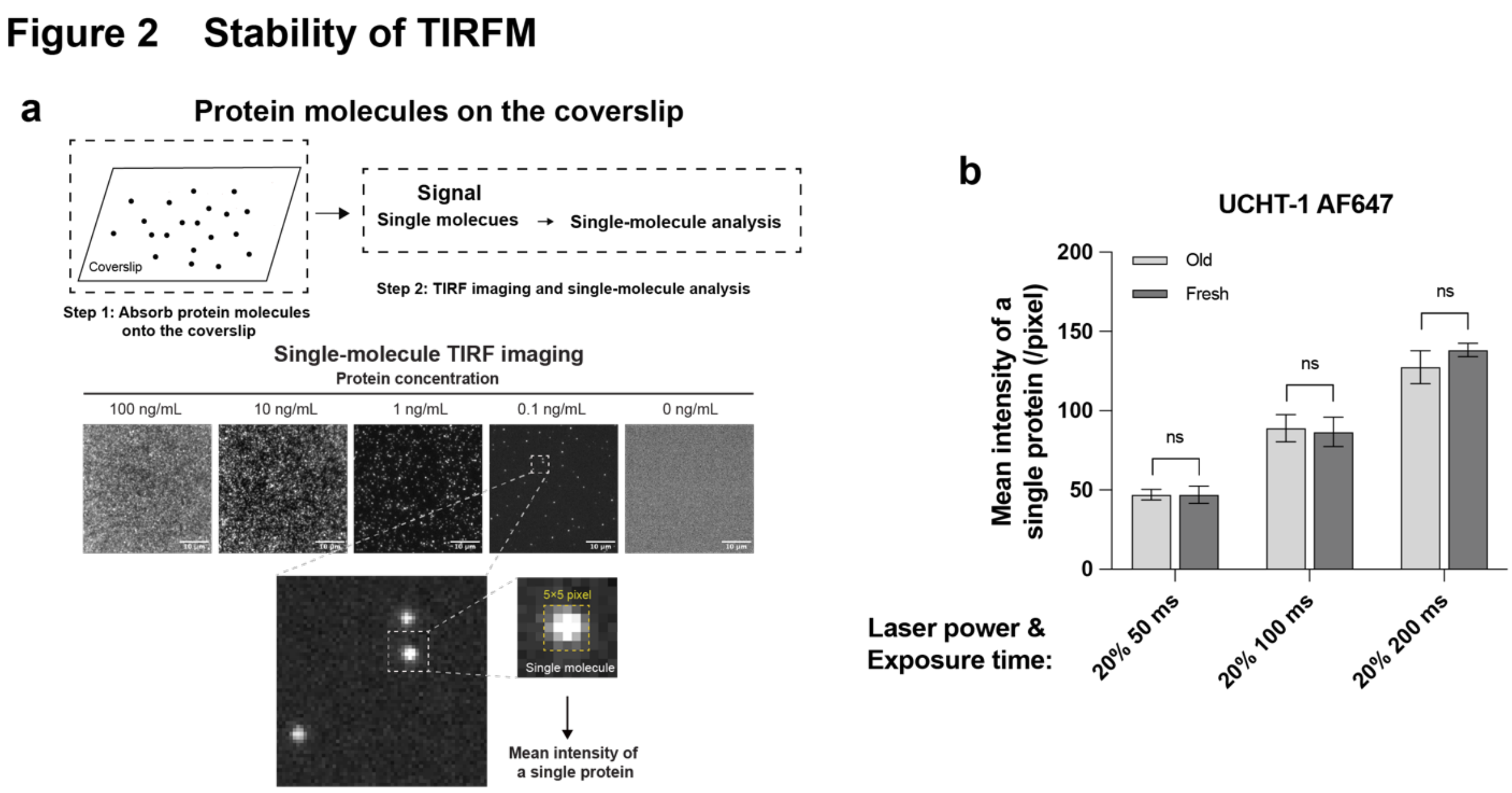

Fig. 2 | Stability of TIRFM. a, Schematic illustrating the method used to isolate and identify single molecules. **b**, Comparison of the mean fluorescence intensity of single UCHT-1 Fab AF647 molecules measured at two different time points. “Fresh” samples were obtained from UCHT-1 Fab AF647 proteins immediately after thawing from the −80 °C freezer. The same thawed protein stock was then stored at 4 °C for two months, after which “old” samples were measured under identical experimental conditions. Data represent mean ± SEM from 16 individual single molecules. Statistical significance was assessed using an unpaired two-tailed Student’s *t*-test: ns indicates no significant difference.

For the concerns about the uniformity of TIRFM in depth

Reviewers may also be concerned about the axial (3D) uniformity of TIRFM. To evaluate this, we compared the mean fluorescence intensity of UCHT-1 Fab AF488 protein molecules located either on the coverslip surface or on the supported lipid bilayer (SLB). Because the thickness of the plasma membrane or planar lipid bilayer is widely known to be ~4-5 nm, this comparison allowed us to assess whether TIRF single-molecule signals decay measurably over this depth range.

For the molecules on the coverslip, we adsorbed UCHT-1 Fab AF488 proteins directly onto the coverslip surface and quantified their mean fluorescence intensities (Fig. 3a, c). For the molecules on the SLB, we diluted UCHT-1 Fab AF488 proteins with UCHT-1 Fab AF647 proteins at a 1:2000 ratio on the SLB (total UCHT-1 density: 36 molecules/μm^2), together with ICAM-1 (177 molecules/μm^2). Upon contacting the SLB, T cell receptors (TCRs) on

CD8⁺ T cells cluster UCHT-1 toward the central region of the T cell–SLB interface. Because of the low density of UCHT-1 Fab AF488 molecules used, we were able to identify and measure the fluorescence intensities of single molecules (Fig. 3b, c). A comparison of the mean fluorescence intensities of single UCHT-1 Fab AF488 molecules on the coverslip versus on the SLB revealed no significant differences (Fig. 3c). These results indicate that TIRFM measurements are homogeneous across the ~10-nm axial range.

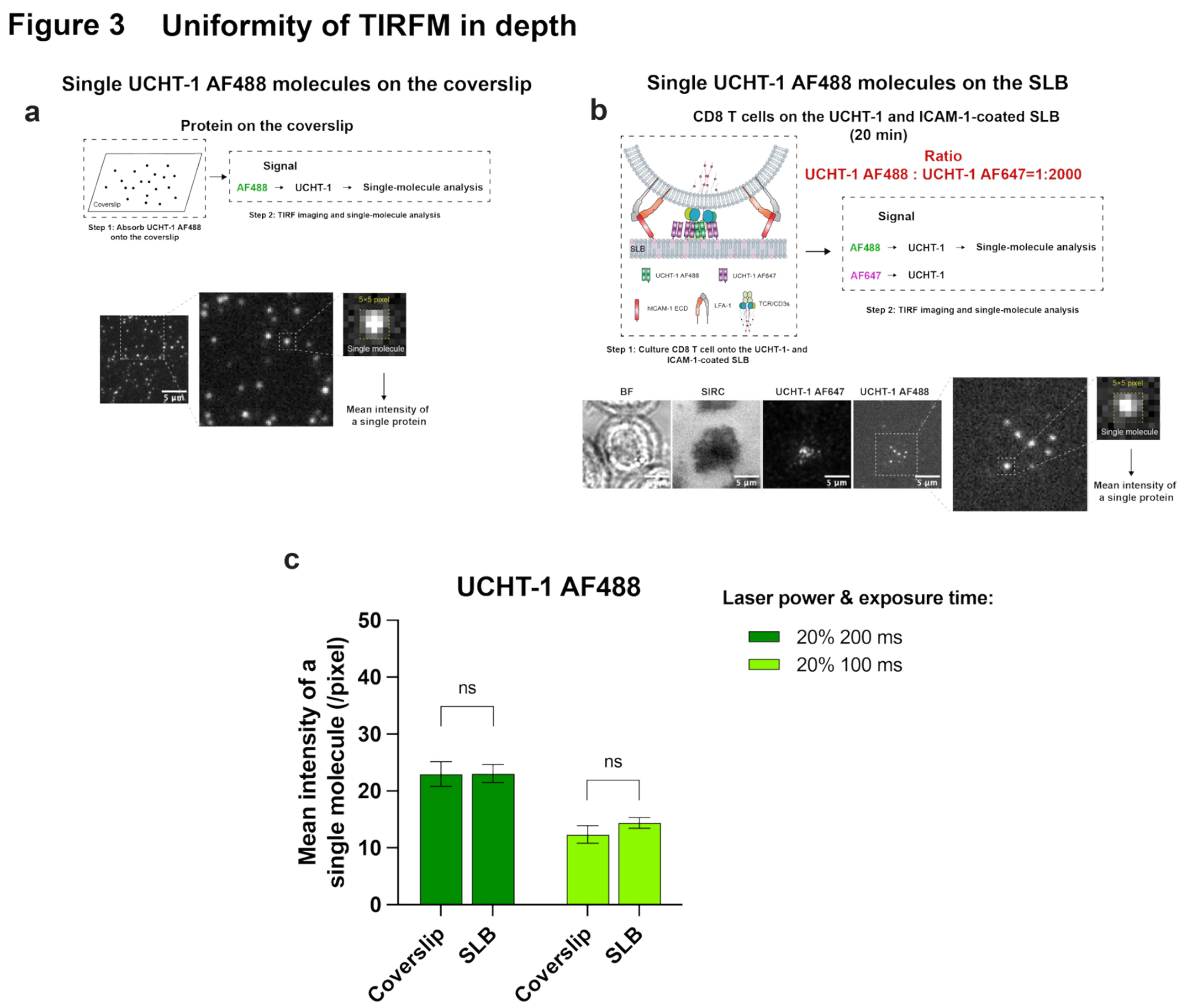

Fig. 3 | Axial uniformity of TIRFM. a, Measurement of the mean fluorescence intensity of single UCHT-1 Fab AF488 molecules adsorbed on the coverslip surface. **b**, Measurement of the mean fluorescence intensity of single UCHT-1 Fab AF488 molecules positioned on the supported lipid bilayer (SLB). **c**, Comparison of the mean fluorescence intensities of single UCHT-1 Fab AF488 molecules at the two depths. Data represent mean ± SEM from 16 individual single molecules. Statistical significance was assessed using an unpaired two-tailed Student's *t*-test: ns indicates no significant difference.

We also examined TIRFM uniformity across a greater depth range. In this experiment, we compared the mean fluorescence intensity of goat anti-rabbit secondary Fab AF647 molecules located either on the coverslip surface or inside cells near the cell–SLB interface.

For the coverslip controls, we adsorbed the secondary Fab AF647 molecules onto the coverslip and quantified single-molecule intensities (Fig. 4a, c). To measure intensities inside cells, we prepared SLBs containing UCHT-1 Fab AF488 (36 molecules/μm^2) and ICAM-1 (177 molecules/μm^2), allowing TCRs on CD8$^+$ T cells to cluster UCHT-1 at the center of the interface. We then fixed and permeabilized the cells, stained them with anti-Zap70 primary antibodies, and subsequently labeled them with diluted goat anti-rabbit secondary Fab AF647. Upon TCR activation, Zap70 is recruited to the membrane-proximal region, placing a substantial fraction of Zap70—and thus the secondary Fab–AF647 probes—within ~20–40 nm of the coverslip (including the thicknesses of the plasma membrane and the SLB). Due to the low concentration of Fab AF647 used, we again resolved single molecules and quantified their fluorescence intensities (Fig. 4b, c). A comparison of the mean intensities of single Fab AF647 molecules on the coverslip versus those located within cells near the interface revealed no significant differences (Fig. 4c).

Together, these data demonstrate that TIRFM provides homogeneous fluorescence measurements over axial depths ranging from 0 to 40 nm.

Figure 4 Uniformity of TIRFM in depth

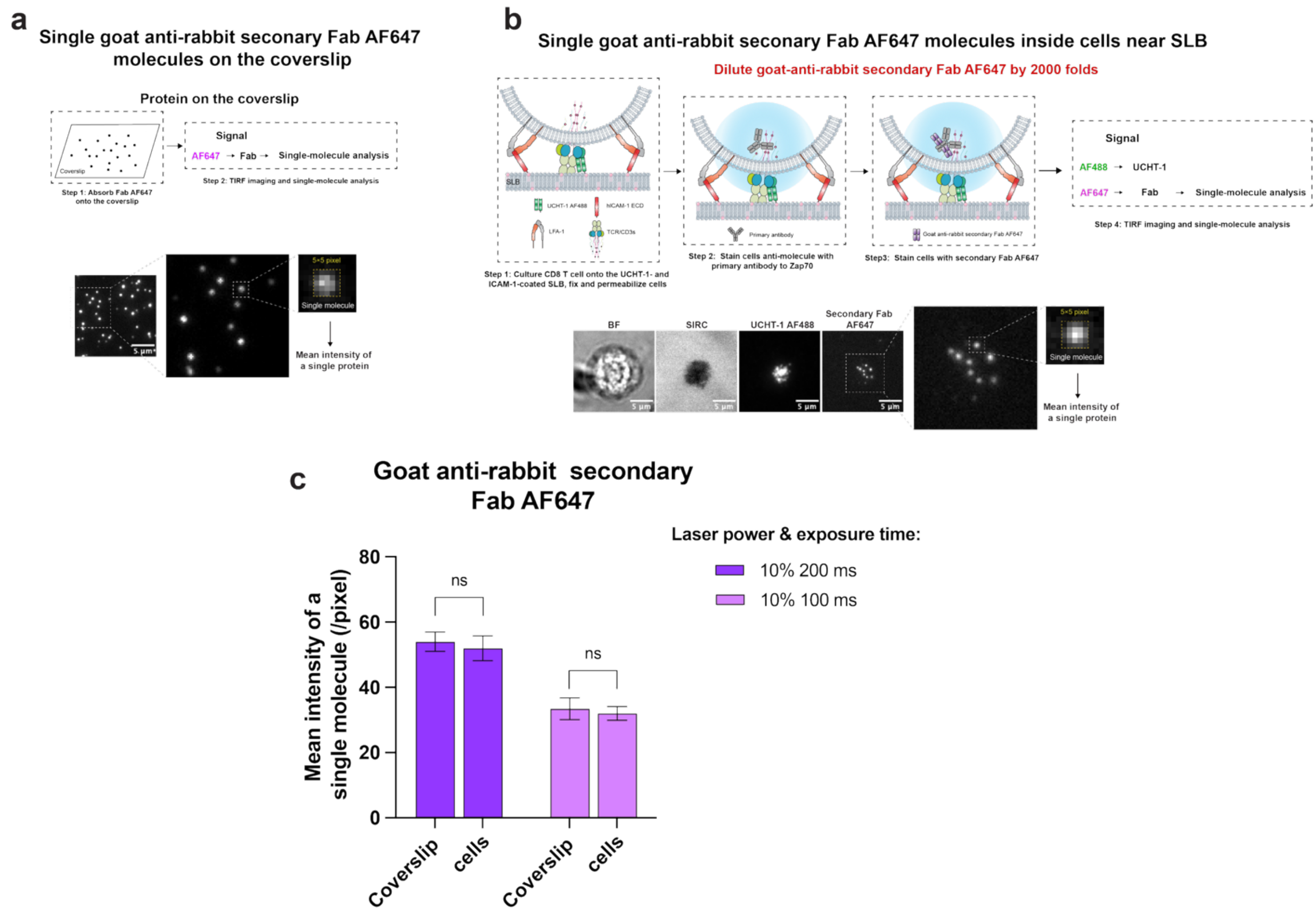

Fig. 4 | Axial uniformity of TIRFM over an extended depth range. a, Measurement of the mean fluorescence intensity of single goat anti-rabbit secondary Fab AF647 molecules adsorbed on the coverslip surface. **b**, Measurement of the mean fluorescence intensity of single goat anti-rabbit secondary Fab AF647 molecules located inside cells near the cell–SLB interface. **c**, Comparison of

the mean fluorescence intensities of single goat anti-rabbit secondary Fab AF647 molecules at the two depths. Data represent mean ± SEM from 16 individual single molecules. Statistical significance was assessed using an unpaired two-tailed Student's *t*-test: ns indicates no significant difference.

These data on uniformity of TIRF are provided here for the reviewers and are available in a biorxiv preprint (2026.03.28.715001v1.) in a preliminary form and currently under peer review.

Finally, regarding concerns about data normalization and cross-experiment comparability of fluorescence in arbitrary units (AU): for each donor, all experimental conditions (all coverslips/SLBs) were imaged on the same day using identical acquisition parameters. Images were processed using the same pipeline and background subtraction rules, and quantitative values were normalized to the control condition from the same donor prior to pooling across biological replicates (see figure legends for details). Statistical tests were therefore performed on normalized values, and all comparisons reflect within-donor differences obtained under matched acquisition sessions. These procedures minimize variability due to day-to-day or slide-to-slide differences and ensure that the reported effects reflect biological differences between conditions rather than imaging artefacts.

4. What is the nature of the IL2 in the SLBs?
Many cytokines (see e.g. Alton-Bonnet for IFNg) and chemokines immobilize to membranes. Is there IL2 already on the SLB at the time of this biology? The setup for the SLB in Figure 1 needs a bit more detail to understand this. Text line ~127 is quite silent on this issue as is the Figure legend. Given the topic of the paper, providing this information here is an important omission (they do so later but don't address whether cytokine might be presented on the SLB. The authors have some recognition as per Figure S1A but that text surrounding that is not supported by the figures, which actually shows no difference between IL2 and no IL2: line 142: "The centralized (focal and annular) phenotypes of IL2 receptor subunits were more frequent when the IS was established in the presence of IL2 (Fig. S1A)."

We thank the reviewer for highlighting the need for clarification regarding the experimental setup.

Both naive and memory $CD8^+$ T cells were cultured without IL2 prior to the experiment, whereas blast $CD8^+$ T cells were maintained in 50 U/mL IL2 for 7 days as part of the standard activation (blasting) protocol. Following mRNA electroporation (18–24 h before imaging), blast $CD8^+$ T cells were kept in 25 U/mL IL2, while naive and memory T cells were maintained without IL2. On the day of the experiment, all cells were washed and subsequently incubated in medium containing or lacking 50 U/mL IL2, as indicated (+/– IL-2). When IL2 was added, it was provided only in soluble form in the culture medium for the duration of the experiment (typically 10–15 min), as specified in each figure legend.

Regarding the experiments presented in Figure 1, as described in the corresponding legend (line 721), immune synapses were formed in the presence of 50 U/mL IL2 in the culture

medium for 10 min for all T cell differentiation states. In the case of Fig. S1 (new S2), synapses formed in the presence or absence of soluble IL2 were compared.

Regarding adsorption of IL2 to the SLB, we have no evidence for this. We found earlier that chemokines could be adsorbed to SLB at only up to 20 molecules/μm^2, but this binding was seen when the bilayers were pre-exposed to pure chemokine solutions and was blocked by pre-treatment of the SLBs with proteins like bovine serum albumin.

To ensure the experimental workflow is fully clear to readers, we have revised the Results section (lines 134) and Materials and Methods (lines 515). Furthermore, we have added a new Supplementary Figure S1, which includes a schematic representation of the experimental workflow to visually clarify these conditions.

5. Receptors being dissociated versus never associated and TIRF depth issues:
Figure 1I is interesting insofar as different patterns are seen for different subunits. Can the authors convince us/themselves that the gamma (being apparently more inside) isn't just a consequence of the fluorophore being used (either being brighter overall or being of a wavelength that results in the TIRF field being deeper?). The discussion provides a precedence for this happening and for differential localizations of various subunits but still would be nice to have the technical explanation invalidated.

We thank the reviewer for this thoughtful comment and the opportunity to clarify the imaging approach used in Figure 1I. The images presented in this panel were acquired using an Airyscan microscope, not by total internal reflection fluorescence microscopy (TIRFM); therefore, potential differences in fluorophore excitation depth associated with TIRF imaging do not apply here.

The "bottom view" represents the lowest XY optical plane, corresponding to the region closest to the cell–SLB interface. The "side view" shows an XZ reconstruction of a cell forming a synapse with the SLB, where the SLB is located at the bottom of the image. This configuration allows visualization of receptor distribution throughout the cell depth, independent of TIRF field penetration or wavelength-related depth effects.

To improve clarity, we have updated the Figure 1I legend accordingly.

Figure 3B/C convincingly shows the main biological point; that pSTAT5 co-localizes with ZAP70 in microvillar contacts in early times, though segregates later. This is enhanced by addition of IL-2 but again does not require it. The text around this appears consistent with the data.

6. Small item of interpretation:
Line 206 the IS activated pSTAT5: since we don't know that STAT5 doesn't activate elsewhere (or is pre-activated) and trafficked to the synapse, phrasing might better be: pSTAT5was localized in the active IS

We thank the reviewer for this helpful suggestion. The text has been revised accordingly to more accurately describe the observation (new line 217).

Just as a note:

The second figure relating to the release of EVs feels less central to the primary focus on IL2R cross talk with TCR that the other figures and story is framed around. While it is definitely interesting to consider the receptor subunit organization in these EVs, these findings may be better suited in extended/supplementary figures so the central manuscript focuses on IL2 receptor localization on the membrane and synapse to set up the justification for exploring how ISTs impact T cells (and why they may be superior to past IL2 trials).

We thank the reviewer for this thoughtful comment and agree that the EV-related findings, while interesting, are peripheral to the main focus on IL2 receptor organization and its cross talk with TCR signaling at the synapse. Therefore, following the reviewer's suggestion, we have moved the previous Figure 2 to the supplementary materials (new Fig. S3). This adjustment keeps the EV data accessible to interested readers while ensuring that the main manuscript remains focused and clear.

And for discussion, perhaps:

Have the authors considered how other cytokines may also feed into this type of synergistic pathway with TCRs? The discussion brings up the trans presentation of IL-15 by APCs but given the sharing of IL2RG across various cytokines that shape T cell differentiation trajectories, there may be subtle differences induced by other cytokines that may be interesting to explore in the discussion. However, adding extra experiments for this would be unnecessary for publication and out of the scope of this paper.

We thank the reviewer for this insightful suggestion. We agree that other common γ-chain cytokines may also interact with TCR signaling at the immunological synapse and potentially generate distinct outcomes due to differences in receptor composition, affinity and downstream STAT usage. Following the reviewer's suggestion, we have expanded the Discussion to briefly address the potential similarities and differences among γc-family cytokines in shaping synaptic signaling and T cell differentiation (line 450-455).

RESPONSE TO REVIEWERS' COMMENTS

Reviewer #1 (Remarks to the Author):

No new comments

Reviewer #4 (Remarks to the Author):

The authors have carefully addressed each of the remarks raised by Reviewer 2.

I just want to point to a couple of issues with the new data added to the revised manuscript:

Related to point 1 raised by Reviewer 2:

New Fig3M: the numbers at the bottom of the figure seem to refer to the number of replicates. This is not needed since the individual replicates can be seen from the dots on the figure (actually 6 and not 5 replicates for the bead SLB condition). What should however be indicated in the legend is what those replicates are (technical replicates? Individual donors? other?).

Thank you for the suggestion. We have clarified in the legend that each data point represents an individual donor. We have retained the n values in the figure legend to comply with the journal's requirements.

New Fig3M: the grey labels of the representative histograms are a bit dim. Maybe black labels would ease the reading.

Thank you for the suggestion. We have changed the histogram labels from grey to black to improve readability.

Related to point 2 raised by Reviewer 2:

New FigS1B: blasts start expressing CD45RO while maintaining CD45RA. Is that expected? CD45RA signal seems to be aberrantly high (actually still very positive signal in memory cells). Can the authors review their flow cytometry analysis and ajust it if applicable?

Thank you for this comment. We have carefully reviewed the flow cytometry analysis and included additional histograms showing CD45RA and CD45RO expression across all $CD8^+$ T cell subsets for clarity. As shown, CD45RA expression is progressively reduced from naive to blast and memory cells, while CD45RO is upregulated upon activation. Thus, the observed phenotype reflects a transitional activation state rather than aberrantly high CD45RA expression.

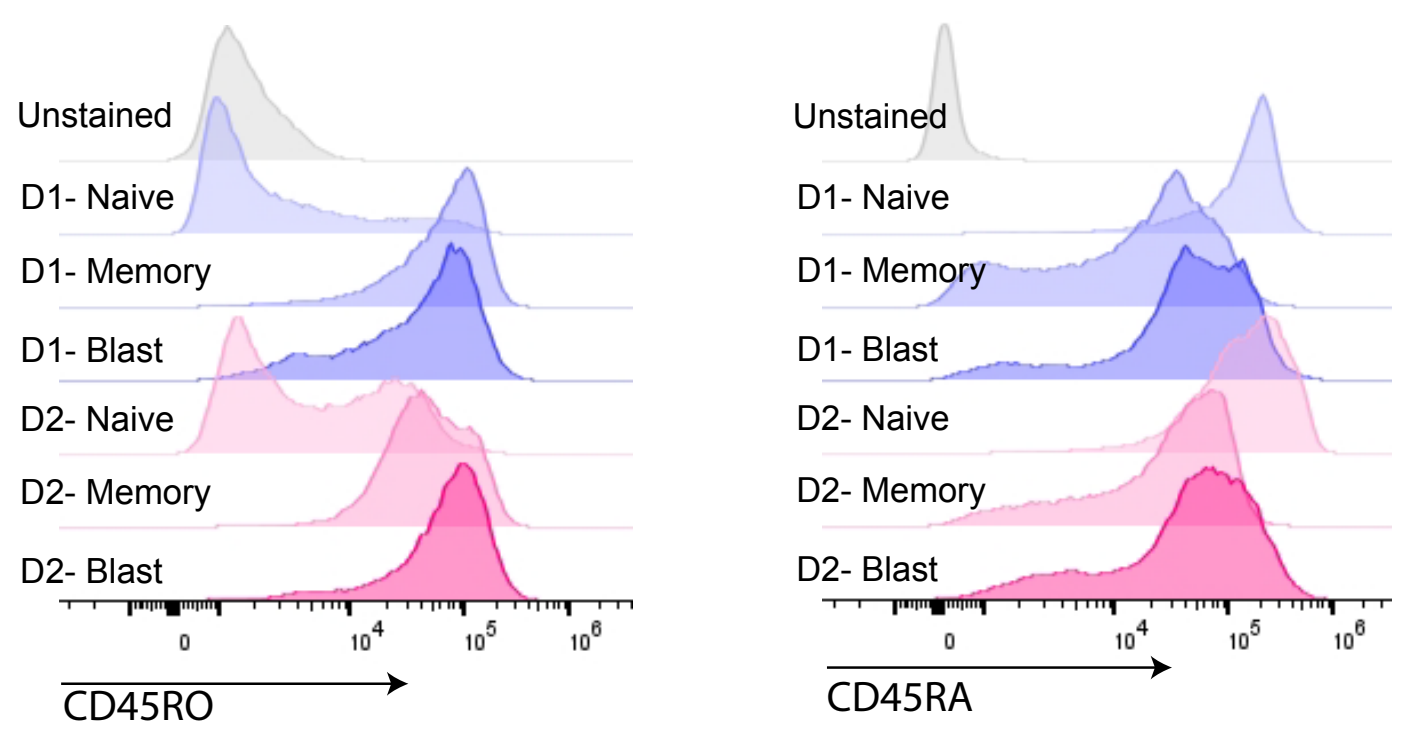

Supplement: Supplementary file 2 [file 41467_2026_77110_MOESM2_ESM.pdf]
