# Supplementary material for: Spatiotemporal co-regulation of IL2 and TCR receptors reveals early antigen-driven synaptic synergy in human CD8+ T cells
Source: Nat Commun. 2026 Oct 7;17:11431. doi: 10.1038/s41467-026-77110-1 (PMC13737045; doi:10.1038/s41467-026-77110-1)
Supplement: Supplementary file 3 [file 41467_2026_77110_MOESM3_ESM.pdf]

nature portfolio

Corresponding author(s): Anish Suri, Michael Dustin

Last updated by author(s): 14/07/26

Reporting Summary

Nature Portfolio wishes to improve the reproducibility of the work that we publish. This form provides structure for consistency and transparency in reporting. For further information on Nature Portfolio policies, see our Editorial Policies and the Editorial Policy Checklist.

Statistics

For all statistical analyses, confirm that the following items are present in the figure legend, table legend, main text, or Methods section.

n/a	Confirmed	
☐	☒	The exact sample size (*n*) for each experimental group/condition, given as a discrete number and unit of measurement
☐	☒	A statement on whether measurements were taken from distinct samples or whether the same sample was measured repeatedly
☐	☒	The statistical test(s) used AND whether they are one- or two-sided<br>*Only common tests should be described solely by name; describe more complex techniques in the Methods section.*
☒	☐	A description of all covariates tested
☐	☒	A description of any assumptions or corrections, such as tests of normality and adjustment for multiple comparisons
☐	☒	A full description of the statistical parameters including central tendency (e.g. means) or other basic estimates (e.g. regression coefficient) AND variation (e.g. standard deviation) or associated estimates of uncertainty (e.g. confidence intervals)
☐	☒	For null hypothesis testing, the test statistic (e.g. F, t, r) with confidence intervals, effect sizes, degrees of freedom and P value noted<br>*Give P values as exact values whenever suitable.*
☒	☐	For Bayesian analysis, information on the choice of priors and Markov chain Monte Carlo settings
☒	☐	For hierarchical and complex designs, identification of the appropriate level for tests and full reporting of outcomes
☐	☒	Estimates of effect sizes (e.g. Cohen's *d*, Pearson's *r*), indicating how they were calculated

Our web collection on statistics for biologists contains articles on many of the points above.

Software and code

Policy information about availability of computer code

Data collection	TIRFM, Olympus IX83. Aryscan, Zeiss 980 LSM. Flow cytometry, LSR II BD Biosciences and Cytek Biosciences. NanoFlow cytometry, NanoFCM. Imaging Flow Cytoemtry, ImageStream MK II (Amnis). Proteomics, AKTA GE Healthcare.
Data analysis	Image analysis and visualisation: ZenBlue 3.3.89.0000 and Fiji 2.14.0. Flow cytometry: FlowJo 10.7.1. scRNAseq analysis: Cell Ranger v3.1.0, Seurat v4.0. Proteomic analysis: MacQuant v1.6.10.43. Imagestream: IDEAS v6.2. Statistical analysis: GraphPad Prism 9.

For manuscripts utilizing custom algorithms or software that are central to the research but not yet described in published literature, software must be made available to editors and reviewers. We strongly encourage code deposition in a community repository (e.g. GitHub). See the Nature Portfolio guidelines for submitting code & software for further information.

Data

Policy information about availability of data

All manuscripts must include a data availability statement. This statement should provide the following information, where applicable:

- Accession codes, unique identifiers, or web links for publicly available datasets
- A description of any restrictions on data availability
- For clinical datasets or third party data, please ensure that the statement adheres to our policy

The mass spectrometry proteomics data generated in this study have been deposited in the PRIDE partner repository of the ProteomeXchange Consortium under accession code PXD070470 [https://www.ebi.ac.uk/pride/archive/projects/PXD070470]. The raw scRNA-seq data were downloaded from the Gene Expression Omnibus (GEO) under accession number GSE126030 [https://www.ncbi.nlm.nih.gov/geo/query/acc.cgi?acc=GSE126030]. The imaging data are available under

restricted access due to their large file size; access can be obtained by contacting the authors.

Research involving human participants, their data, or biological material

Policy information about studies with human participants or human data. See also policy information about sex, gender (identity/presentation), and sexual orientation and race, ethnicity and racism.

Reporting on sex and gender	Human samples were fully anonymized prior to acquisition; accordingly, these variables were not included in the study design.
Reporting on race, ethnicity, or other socially relevant groupings	Human samples were fully anonymized prior to acquisition; accordingly, these variables were not included in the study design.
Population characteristics	Human samples were fully anonymized prior to acquisition; accordingly, these variables were not included in the study design.
Recruitment	Primary human T cells were isolated from leukoreduction system (LRS) chambers, which were supplied by the UK National Health Service Blood and Transplant and sourced from healthy, anonymous donors.
Ethics oversight	REC reference 11/H0711/7, IRAS project ID 66954<br>Committee: NHS Health Research Authority, National Research Ethics Service (NRES)

Note that full information on the approval of the study protocol must also be provided in the manuscript.

Field-specific reporting

Please select the one below that is the best fit for your research. If you are not sure, read the appropriate sections before making your selection.

☒ Life sciences ☐ Behavioural & social sciences ☐ Ecological, evolutionary & environmental sciences

For a reference copy of the document with all sections, see nature.com/documents/nr-reporting-summary-flat.pdf

Life sciences study design

All studies must disclose on these points even when the disclosure is negative.

Sample size	Sample sizes were determined based on commonly accepted standards in the field and previous related studies. We typically used three biological replicates to ensure reproducibility and statistical robustness.
Data exclusions	No data was excluded from studies.
Replication	We confirmed that all repeated attempts were successful. Experiment repeat numbers are reported in Figures.
Randomization	Randomisation was not applicable, as all conditions to be compared originated from the same cell preparation (same donor) and were processed in parallel to control for variability.
Blinding	Blinding was not performed, as all conditions were processed in parallel from the same cell preparation by the same researcher and experimental readouts required direct identification of sample conditions during acquisition.

Reporting for specific materials, systems and methods

We require information from authors about some types of materials, experimental systems and methods used in many studies. Here, indicate whether each material, system or method listed is relevant to your study. If you are not sure if a list item applies to your research, read the appropriate section before selecting a response.

Materials & experimental systems

n/a	Involved in the study	
☐	☒	Antibodies
☒	☐	Eukaryotic cell lines
☒	☐	Palaeontology and archaeology
☒	☐	Animals and other organisms
☒	☐	Clinical data
☒	☐	Dual use research of concern
☒	☐	Plants

Methods

n/a	Involved in the study	
☒	☐	ChIP-seq
☐	☒	Flow cytometry
☒	☐	MRI-based neuroimaging

Antibodies

Antibodies used	anti-pSTAT5 AF647, BD Biosciences, RRID: AB_399882, dilution 1:5<br>anti-pZAP70 PE BD Biosciences, RRID: AB_396919, dilution 1:5<br>anti-IL2RA FITC clone 2A3 BD Biosciences, RRID: AB_400334, dilution 1:5<br>anti-IL2RB APC clone Mik-β3 BD Biosciences, RRID: AB_2869796, dilution 1:5<br>anti-IL2RG, ThermoFisher, RRID: AB_2856707, dilution 1:100<br>anti-CD3 PE, BioLegend, RRID: AB_2562047, dilution 1:20<br>anti-IL2, clone MQ1-17H12, Biolegend, RRID: AB_315088, dilution 1:20<br>anti-CD3 PE, clone UCHT1, Biolegend, RRID: AB_2562047, dilution 1:100<br>anti-CD19-AF594, Biolegend, RRID: AB_2563233, dilution 1:20<br>anti-CD14-BV421, Biolegend, RRID: AB_2561342 , dilution 1:20<br>anti-CD8-AF700, Biolegend, RRID: AB_2562789, dilution 1:20<br>anti-CD4-APC/Fire750, Biolegend, RRID: AB_2716182, dilution 1:20<br>anti-CD56-APC/Fire750, Biolegend, RRID: AB_2572104, dilution 1:20<br>anti-CD64, clone 10.1 Biolegend, RRID: AB_314486, dilution 1:20
Validation	All antibodies were validated by the supplier as stated below:<br>anti-pSTAT5 AF647, BD Biosciences; https://www.bdbiosciences.com/en-at/products/reagents/flow-cytometry-reagents/research-reagents/single-color-antibodies-ruo/alexa-fluor-647-mouse-anti-stat5-py694.612599?tab=product_details<br>anti-pZAP70 PE BD Biosciences, https://www.bdbiosciences.com/en-au/products/reagents/flow-cytometry-reagents/research-reagents/single-color-antibodies-ruo/PE-Mouse-Anti-Zap70-(Y319)-Syk-(Y352).557881?tab=product_details<br>anti-IL2RA FITC clone 2A3 BD Biosciences, https://www.bdbiosciences.com/en-nz/products/reagents/flow-cytometry-reagents/clinical-discovery-research/single-color-antibodies-ruo-gmp/fitc-mouse-anti-human-cd25.347643?tab=product_details<br>anti-IL2RB APC clone Mik-β3 BD Biosciences, https://www.bdbiosciences.com/en-br/products/reagents/flow-cytometry-reagents/research-reagents/single-color-antibodies-ruo/apc-mouse-anti-human-cd122.566620?tab=product_details<br>anti-IL2RG, ThermoFisher, RRID: AB_2856707, https://www.thermofisher.com/antibody/product/IL2RG-Antibody-Polyclonal/PA5-111297<br>anti-CD3 PE, BioLegend, RRID: AB_2562047, https://www.biolegend.com/fr-lu/products/pe-anti-human-cd3-antibody-865<br>anti-IL2, clone MQ1-17H12, Biolegend, RRID: AB_315088, https://www.biolegend.com/en-dk/products/purified-anti-human-il-2-antibody-1352?pdf=true&displayInline=true&leftRightMargin=15&topBottomMargin=15&filename=Purified%20anti-human%20IL-2%20Antibody.pdf&v=20250724033835<br>anti-CD19-AF594, Biolegend, RRID: AB_2563233, https://www.biolegend.com/fr-ch/products/alexa-fluor-594-anti-human-cd19-antibody-9669<br>anti-CD14-BV421, Biolegend, RRID: AB_2561342, https://www.biolegend.com/fr-ch/products/brilliant-violet-421-anti-human-cd14-antibody-7203<br>anti-CD8-AF700, Biolegend, RRID: AB_2562789, https://www.biolegend.com/de-at/products/alexa-fluor-700-anti-human-cd8-antibody-9062<br>anti-CD4-APC/Fire750, Biolegend, RRID: AB_2716182, https://www.biolegend.com/fr-ch/products/apcfire-750-anti-human-cd4-antibody-14905<br>anti-CD56-APC/Fire750, Biolegend, RRID: AB_2572104, https://www.biolegend.com/fr-fr/products/apc-fire-750-anti-human-cd56-ncam-antibody-13034<br>anti-CD64, clone 10.1 Biolegend, RRID: AB_314486, https://www.biolegend.com/fr-ch/products/purified-anti-human-cd64-antibody-538

Plants

Seed stocks	NA
Novel plant genotypes	NA
Authentication	NA

Flow Cytometry

Plots

Confirm that:

- [x] The axis labels state the marker and fluorochrome used (e.g. CD4-FITC).
- [x] The axis scales are clearly visible. Include numbers along axes only for bottom left plot of group (a 'group' is an analysis of identical markers).
- [x] All plots are contour plots with outliers or pseudocolor plots.
- [x] A numerical value for number of cells or percentage (with statistics) is provided.

Methodology

Sample preparation	Primary human T cells were isolated from leukoreduction system (LRS) chambers. CD8+ T cells were purified using the negative selection kits RosetteSep™ Human CD8+ T Cell Enrichment Cocktail (StemCell Technologies), EasySep™ Human Naive CD8+ T Cell or EasySep™ Human Memory CD8+ T Cell Isolation Kits (StemCell Technologies)
Instrument	LSR II Flow Cytometer (BD Biosciences), Flow NanoAnalyzer (NanoFCM Co., LTD) and ImageStream MK II (Amnis)
Software	FlowJo version 10.7.1 and ImageStream INSPIRE V4 and IDEAS 6.2
Cell population abundance	The target cell population represented close to 100% of the analysed sample.
Gating strategy	Flow cell: Cells were first gated on FSC/SSC. Singlet cells were gated using FSC-H and FSC-A. ImageStream: In-focus images were used to gate doublets based on the area and the aspect ratio of the bright field images. Subsequent gating was carried out using the specific marker staining.

- [x] Tick this box to confirm that a figure exemplifying the gating strategy is provided in the Supplementary Information.
